# Functional and clinical studies reveal pathophysiological complexity of *CLCN4*-related neurodevelopmental condition

**DOI:** 10.1038/s41380-022-01852-9

**Published:** 2022-11-16

**Authors:** Elizabeth E. Palmer, Michael Pusch, Alessandra Picollo, Caitlin Forwood, Matthew H. Nguyen, Vanessa Suckow, Jessica Gibbons, Alva Hoff, Lisa Sigfrid, Andre Megarbane, Mathilde Nizon, Benjamin Cogné, Claire Beneteau, Fowzan S. Alkuraya, Aziza Chedrawi, Mais O. Hashem, Hannah Stamberger, Sarah Weckhuysen, Arnaud Vanlander, Berten Ceulemans, Sulekha Rajagopalan, Kenneth Nunn, Stéphanie Arpin, Martine Raynaud, Constance S. Motter, Catherine Ward-Melver, Katrien Janssens, Marije Meuwissen, Diane Beysen, Nicola Dikow, Mona Grimmel, Tobias B. Haack, Emma Clement, Amy McTague, David Hunt, Sharron Townshend, Michelle Ward, Linda J. Richards, Cas Simons, Gregory Costain, Lucie Dupuis, Roberto Mendoza-Londono, Tracy Dudding-Byth, Jackie Boyle, Carol Saunders, Emily Fleming, Salima El Chehadeh, Marie-Aude Spitz, Amelie Piton, Bénédicte Gerard, Marie-Thérèse Abi Warde, Gillian Rea, Caoimhe McKenna, Sofia Douzgou, Siddharth Banka, Cigdem Akman, Jennifer M. Bain, Tristan T. Sands, Golder N. Wilson, Erin J. Silvertooth, Lauren Miller, Damien Lederer, Rani Sachdev, Rebecca Macintosh, Olivier Monestier, Deniz Karadurmus, Felicity Collins, Melissa Carter, Luis Rohena, Marjolein H. Willemsen, Charlotte W. Ockeloen, Rolph Pfundt, Sanne D. Kroft, Michael Field, Francisco E. R. Laranjeira, Ana M. Fortuna, Ana R. Soares, Vincent Michaud, Sophie Naudion, Sailaja Golla, David D. Weaver, Lynne M. Bird, Jennifer Friedman, Virginia Clowes, Shelagh Joss, Laura Pölsler, Philippe M. Campeau, Maria Blazo, Emilia K. Bijlsma, Jill A. Rosenfeld, Christian Beetz, Zöe Powis, Kirsty McWalter, Tracy Brandt, Erin Torti, Mikaël Mathot, Shekeeb S. Mohammad, Ruth Armstrong, Vera M. Kalscheuer

**Affiliations:** 1 Centre for Clinical Genetics, Sydney Children’s Hospital Network, Randwick, NSW Australia; 2 Discipline of Paediatrics and Child Health, Faculty of Medicine and Health, University of New South Wales, Randwick, NSW Australia; 3 Istituto di Biofisica, CNR, Genova, Italy; 4 Department of Clinical Genetics, Liverpool Hospital, Liverpool, NSW Australia; 5 Max Planck Institute for Molecular Genetics, Group Development and Disease, Berlin, Germany; 6 Department of Biomedical and Clinical Sciences, Linköping University, Linköping, 581 83 Sweden; 7 Department of Human Genetics, Gilbert and Rose-Marie Chagoury School of Medicine, Lebanese American University, Byblos, Lebanon; 8 Institut Jerome Lejeune, Paris, France; 9 Service de Génétique Médicale, CHU de Nantes, Nantes Université, Nantes, France; 10 Nantes Université, CNRS, INSERM, l’Institut du Thorax, Nantes, France; 11 Department of Translational Genomics, Center for Genomic Medicine, King Faisal Specialist Hospital and Research Center, Riyadh, Saudi Arabia; 12 Department of Neurosciences, King Faisal Specialist Hospital and Research Center, Riyadh, Saudi Arabia; 13 Applied and Translational Neurogenomics Group, VIB Center for Molecular Neurology, VIB, Antwerp, Belgium; 14 Neurology Department, Antwerp University Hospital, Antwerp, Belgium; 15 Translational Neurosciences, Faculty of Medicine and Health Science, University of Antwerp, Antwerp, Belgium; 16 Department of Child Neurology & Metabolism, Ghent University Hospital, Ghent, Belgium; 17 Department of Pediatric Neurology, Antwerp University Hospital, University of Antwerp, Antwerp, Belgium; 18 Children’s Hospital at Westmead, Sydney Children’s Hospitals Network, Sydney, Australia; 19 Service de Génétique Clinique, Centre Hospitalier Régional Universitaire de Tours, Tours, France; 20 Genetic Center, Akron Children’s Hospital, Akron, OH USA; 21 Center of Medical Genetics, University Hospital Antwerp/University of Antwerp, Edegem, Belgium; 22 Department of Pediatric Neurology, University Hospital Antwerp/University of Antwerp, Edegem, Belgium; 23 Institute of Human Genetics, Heidelberg University, Heidelberg, Germany; 24 Institute of Medical Genetics and Applied Genomics, University of Tuebingen, Tuebingen, Germany; 25 Department of Clinical Genetics, Great Ormond Street Hospital for Children, London, UK; 26 Developmental Neurosciences, UCL Great Ormond Street Institute of Child Health, London, UK; 27 Department of Neurology, Great Ormond Street Hospital, London, UK; 28 Wessex Clinical Genetics Service, Princess Anne Hospital, Southampton, UK; 29 Genetic Services of WA, King Edward Memorial Hospital, Subiaco, WA Australia; 30 Department of Neuroscience, Washington University in St Louis School of Medicine, St Louis, MI USA; 31 The University of Queensland, Queensland Brain Institute, St Lucia, QLD Australia; 32 Centre for Population Genomics, Murdoch Children’s Research Institute, Melbourne, Australia; 33 Garvan Institute of Medical Research, UNSW, Sydney, NSW Australia; 34 Division of Clinical and Metabolic Genetics, The Hospital for Sick Children, Toronto, ON Canada; 35 Genetics of Learning Disability Service, Newcastle, NSW Australia; 36 University of Newcastle Grow Up Well Priority Research Centre, Newcastle, NSW Australia; 37 Department of Pathology and Laboratory Medicine, Children’s Mercy Hospital and Clinics, MI Kansas City, USA; 38 Kansas City School of Medicine, University of Missouri, Kansas City, MI USA; 39 Division of Clinical Genetics, Children’s Mercy Hospital and Clinics, Kansas City, MI USA; 40 Service de Génétique Médicale, Institut de Génétique Médicale d’Alsace (IGMA), Hôpitaux Universitaires de Strasbourg, Strasbourg, France; 41 Laboratoire de Génétique Médicale, UMRS_1112, Institut de Génétique Médicale d’Alsace (IGMA), Université de Strasbourg et INSERM, Strasbourg, France; 42 Service de Pédiatrie, Hôpital de Hautepierre, Hôpitaux Universitaires de Strasbourg, Strasbourg, France; 43 Laboratoires de Diagnostic Génétique, Institut de Génétique Médicale d’Alsace (IGMA), Hôpitaux Universitaires de Strasbourg, Nouvel Hôpital Civil, Strasbourg, France; 44 Pediatric Neurology Department, CHU de Strasbourg, Strasbourg, France; 45 Northern Ireland Regional Genetics Service, Belfast, Northern Ireland; 46 Department of Medical Genetics, Haukeland University Hospital, Bergen, Norway; 47 Division of Evolution, Infection and Genomics, School of Biological Sciences, Faculty of Biology, Medicine and Health, University of Manchester, Manchester, UK; 48 Manchester Centre for Genomic Medicine, Saint Mary’s Hospital, Manchester University NHS Foundation Trust, Manchester, UK; 49 Department of Neurology, Division of Child Neurology, Columbia University Irving Medical Center, New York, USA; 50 Texas Tech Health Sciences Center Lubbock and KinderGenome Medical Genetics, Dallas, TX USA; 51 Texas Sports Psychiatry and Integrative Health, Austin, TX USA; 52 Hillcrest Internal Medicine, Waco, TX USA; 53 Centre de Génétique Humaine, Institut de Pathologie et de Génétique ASBL, Gosselies, Belgium; 54 Department of Medical Genomics/Clinical Genetics, Royal Prince Alfred Hospital, Camperdown, Sydney, NSW Australia; 55 Department of Genetics, Children’s Hospital of Eastern Ontario, Ottawa, ON Canada; 56 Division of Medical Genetics, Department of Pediatrics, San Antonio Military Medical Center, San Antonio, TX USA; 57 Department of Pediatrics, Long School of Medicine-UT Health San Antonio, San Antonio, TX USA; 58 Department of Human Genetics, Radboud University Medical Center, Nijmegen, The Netherlands; 59 Pluryn, Residential Care Setting, Groesbeek, The Netherlands; 60 Centro de Genética Médica Jacinto Magalhães, Centro Hospitalar Universitário do Porto, Porto, Portugal; 61 Unit for Multidisciplinary Research in Biomedicine, School of Medicine and Biomedical Sciences, Porto University, Porto, Portugal; 62 Service de Génétique Médicale, CHU Bordeaux, Bordeaux, France; 63 INSERM U1211, Laboratoire Maladies Rares: Génétique et Métabolisme, Bordeaux, Univ., Bordeaux, France; 64 Child Neurology and Neurodevelopmental Medicine Thompson Autism Center, CHOC Hospital, Orange County, CA USA; 65 Indiana University School of Medicine, Indianapolis, USA; 66 University of California, San Diego, Rady Children’s Hospital San Diego, San Diego, CA USA; 67 North West Thames Regional Genetics Service, London North West University Healthcare NHS Trust, Harrow, London, UK; 68 Imperial College London, London, UK; 69 West of Scotland Centre for Genomic Medicine, Queen Elizabeth University Hospital, Glasgow, UK; 70 Centrum Medische Genetica, Universitair Ziekenhuis Brussel, Vrije Universiteit Brussel (VUB), Brussels, Belgium; 71 CHU Sainte-Justine Research Center, University of Montreal, Montreal, QC Canada; 72 Division Clinical Genetics Texas A&M University Health Science Center, College Station, TX USA; 73 Department of Clinical Genetics, Leiden University Medical Centre, Leiden, The Netherlands; 74 Molecular and Human Genetics, Baylor College of Medicine, Houston, TX USA; 75 Baylor Genetics Laboratories, Houston, TX USA; 76 Centogene GmbH, Rostock, Germany; 77 Clinical Genomics, Ambry Genetics, Aliso Viejo, CA USA; 78 GeneDx LLC, Gaithersburg, MA USA; 79 Neuropediatric Unit, CHU UCL-Namur, Namur, Belgium; 80 Children’s Hospital at Westmead, Sydney Children’s Hospitals Network, Sydney, NSW Australia; 81 East Anglian Medical Genetics Service, Clinical Genetics, Addenbrooke’s Treatment Centre, Addenbrooke’s Hospital, Cambridge, UK

**Keywords:** Autism spectrum disorders, Genetics

## Abstract

Missense and truncating variants in the X-chromosome-linked *CLCN4* gene, resulting in reduced or complete loss-of-function (LOF) of the encoded chloride/proton exchanger ClC-4, were recently demonstrated to cause a neurocognitive phenotype in both males and females. Through international clinical matchmaking and interrogation of public variant databases we assembled a database of 90 rare *CLCN4* missense variants in 90 families: 41 unique and 18 recurrent variants in 49 families. For 43 families, including 22 males and 33 females, we collated detailed clinical and segregation data. To confirm causality of variants and to obtain insight into disease mechanisms, we investigated the effect on electrophysiological properties of 59 of the variants in *Xenopus* oocytes using extended voltage and pH ranges. Detailed analyses revealed new pathophysiological mechanisms: 25% (15/59) of variants demonstrated LOF, characterized by a “shift” of the voltage-dependent activation to more positive voltages, and nine variants resulted in a toxic gain-of-function, associated with a disrupted gate allowing inward transport at negative voltages. Functional results were not always in line with in silico pathogenicity scores, highlighting the complexity of pathogenicity assessment for accurate genetic counselling. The complex neurocognitive and psychiatric manifestations of this condition, and hitherto under-recognized impacts on growth, gastrointestinal function, and motor control are discussed. Including published cases, we summarize features in 122 individuals from 67 families with *CLCN4*-related neurodevelopmental condition and suggest future research directions with the aim of improving the integrated care for individuals with this diagnosis.

## Introduction

*CLCN4* encodes the intracellularly located chloride/proton ion-exchanger ClC-4, and is located on the human X chromosome at Xp22.2. Rare inherited or de novo missense and truncating variants are identified in a growing number of males and females with a range of neurodevelopmental and psychiatric complications. However, the establishment of the pathogenicity of previously unreported rare missense variants remains challenging. As of 22nd May 2022, from the 153 missense *CLCN4* variants listed in the publicly available database ClinVar, 73% (111) were classified to be of uncertain significance. Without clear establishment of pathogenicity, families remain on a diagnostic odyssey, cannot make fully informed reproductive choices, or benefit from advances in condition-specific management guidelines or targeted therapies.

The first *CLCN4* variant was reported in an infant male with developmental and epileptic encephalopathy and suggested *CLCN4* as a novel candidate disease gene [1]. Three years later, as part of an X chromosomal exome sequencing study, our group demonstrated that truncating and missense variants were associated with a neurocognitive phenotype in males in five unrelated families [2]. Two families had linkage intervals including Xp22: A two generation French family with five affected males with severe to profound intellectual disability (ID) and variable behavioral difficulties was reported by Raynaud et al., in 1996 [3] and a Belgian family with five males spanning two generations with ID, challenging behaviors and autistic features described by Claes et al. [4]. Heterozygous females in those families were neurotypical or had a mild neurocognitive/psychiatric phenotype. Therefore, a phenotypic entity of X-linked recessive ID (Raynaud-Claes Syndrome) was proposed (MIM *300114).

Subsequently, we reported 10 additional families consisting of 29 hemizygous males and 23 heterozygous females [5]. We clarified that all males had a core phenotype of mild to severe ID, with considerable intrafamilial heterogeneity. For the first time, we reported the phenotype in females with *de novo* variants, which overlapped in severity with that of males. Other common clinical features included epilepsy, subtle white matter changes on neuroimaging, autism spectrum disorder, challenging behaviors, and mental health complications including bipolar disorder, depression, and anxiety. More recently, an additional six males with *CLCN4*-related neurodevelopmental condition were reported confirming the core feature of ID and common comorbidities of epilepsy and challenging behaviors [2, 6, 7]. Xu et al., reported on a female with ID, autistic features and brain abnormalities, with a maternally inherited *CLCN4* missense variant where the mother had mild ID [8]. We recently summarized the published genotypic and phenotypic spectrum [9], noting that, to date, all *CLCN4* variants studied in the *Xenopus* expression system demonstrated partial or complete loss-of-function (LOF) [1, 10, 11].

ClC-4 is one of the nine members of the CLC gene family encoding anion-transporting membrane proteins [12]. CLC proteins are divided into two groups: four members (ClC-1, ClC-2, ClC-Ka, and ClC-Kb) are Cl^−^ channels localized in the plasma membrane, while the remaining CLCs (ClC-3 to -7) are secondary active Cl^−^/H^+^ antiporters physiologically localized in intracellular endo-/lysosomal membranes; the latter are also called vesicular CLCs (vCLCs). Among the vCLCs, ClC-3 to -5 are highly homologous and are localized to endosomes, while the more distantly related ClC-6 and ClC-7 are localized to late endosomes and lysosomes, respectively [12]. The vesicular Cl^−^/H^+^ antiport activity is important for ionic homeostasis of endo-/lysosomes by assisting in vesicular acidification and increasing luminal Cl^−^ concentration. The function of ClC-4 critically depends on the highly related ClC-3 transporter, with which it forms heterodimers [13, 14]. While most CLCs are physiologically homodimeric, ClC-4 appears to preferentially associate with ClC-3, whereas ClC-4 homodimers are biochemically relatively unstable [13, 14].

ClC-4, and other members of this protein family, ClC-3, ClC-6, ClC-7, and Ostm1, an obligatory subunit of ClC-7, are implicated in neurological disorders [2, 5, 12, 15, 16]. This could be postulated to be related to the postmitotic nature of neurons and their heavy reliance on vesicular trafficking. For example, mice lacking late endosomal ClC-6 transporters show signs of lipofuscin accumulation [17], and lacking lysosomal ClC-7 exhibit a severe lysosomal storage phenotype, respectively [18]. Recently a recurrent gain-of-function (GOF) variant reported in *CLCN6* caused the severe neurodegenerative disease CONRIBA (Neurodegeneration, childhood-onset, hypotonia, respiratory insufficiency and brain imaging abnormalities CONRIBA; MIM 619173) [15] while a variant found in a patient with clinical features of late-onset neuronal ceroid lipofuscinosis [17] was found to have greatly reduced functional activity [19]. LOF of ClC-3 in mice leads to neurodegeneration [20] and both GOF and LOF *CLCN3* variants in humans cause severe global developmental delay [16]. Conversely, knock-out mouse models of ClC-4 have no overt phenotype [21], implying a complex causative mechanism that requires further exploration to understand the pathophysiological basis of *CLCN4*-related neurodevelopmental condition.

Understanding the pathogenicity of missense variation in *CLCN4* both clinically and functionally is therefore the next step [6]. We firstly undertook a collaborative study aiming to further characterize the genotypic and phenotypic spectrum of *CLCN4*-related neurodevelopmental condition in both males and females. Secondly, we studied the functional impact of novel and previously reported missense variants in heterologously expressing *Xenopus* oocytes by employing electrophysiological measurements using extended voltage-protocols.

## Subjects and methods

### Subjects

We collected de-identified detailed clinical data on 55 individuals from 43 previously unreported families with (presumed) *CLCN4*-related neurodevelopmental condition, including individuals from three families where the proband had a blended clinical phenotype with a second genetic diagnosis. Data were obtained through an international collaborative process wherein clinicians and diagnostic laboratories with variants identified in *CLCN4* contacted our team, and we also contacted the laboratory or clinician who had deposited variants in *CLCN4* in the public databases DECIPHER, ClinVar, and LOVD [22–24]. In each participating center, written informed consent was obtained from the individual’s legal guardians before genetic testing as approved by relevant local ethical committees. Clinical information was obtained by review of medical records and examination of affected individuals. Written informed consent for the publication of clinical data and photographs was also obtained from the participants’ legal guardians.

### Expression construct

The human ClC-4 cDNA was cloned in the pTLN expression vector [25], in which the disease-associated variants were introduced using standard restriction-free mutagenesis. All constructs were verified by Sanger sequencing.

### Expression in oocytes

RNA was transcribed using the SP6 mMessageMachine kit (Thermofisher, Milan, Italy) after linearization with *Mlu*I. *Xenopus laevis* oocytes were injected with ~6 ng of RNA and incubated at 18 °C for 2–5 days prior to measurements as described previously [26].

### Two electrode voltage clamp recordings

Recording pipettes were filled with 3 M KCl (resistance about 0.6 MOhm) and currents were recorded using a TEC03 two electrode voltage clamp amplifier (npi electronics, Tamm, Germany). Ground electrodes were connected to the bath via agar bridges. The standard extracellular solution contained 100 mM NaCl, 5 mM MgSO_4_, 10 mM HEPES (pH 7.3). For solutions at pH 6.3 and 5.3, HEPES buffer was replaced by MES (2-(n-morpholino) ethanesulfonic acid) buffer. pH was adjusted with NaOH. Currents were acquired using the custom GePulse acquisition program and an itc-16 interface (Instrutech, Colorado, USA), filtering at 5 kHz and sampling at 50 kHz. Two types of stimulation protocols were applied from a holding potential of −30 mV. The first consisted of 10 ms pulses to voltages ranging from +160 to −120 mV (in 20 mV steps) without leak-subtraction. The second protocol consisted of steps ranging from +170 to −10 mV (in 10 mV steps), applying linear leak and capacity subtraction using a ‘P/4’ leak subtraction protocol from the holding potential −30 mV. For this procedure 4 pulses of ¼ of the regular amplitude were applied towards negative voltages, their response was averaged, adequately scaled, and subtracted. This procedure approximately eliminates linear capacitive currents and ‘leak’, assuming that ClC-4 is inactive at negative voltages.

### Data analysis

To evaluate the relative expression levels of mutant compared to wild-type (WT) ClC-4, currents were measured for >=6 oocytes for each batch of injection of each construct, and the average current-voltage relationship was obtained using the P/4 subtracted protocol. Average currents from > =6 non-injected oocytes from the same batch were subtracted. For the average IV curves, currents were normalized to the current measured for WT from the same batch at 170 mV, and data from at least four injections for each construct were averaged. For the average ratios of mutant versus WT currents at a given voltage, data, currents were normalized to the respective current measured for WT. This procedure highlights possible alterations of the voltage-dependence. A voltage-independent reduction (or increase) in current size would result in a voltage-independent ratio. We interpret alterations of the voltage-dependent rectification as a change of a gating process that depends on both subunits. Such a gating process is clearly present in ClC-6 and ClC-7 transporters [19, 27] and similarly most likely underlies the extreme rectification of ClC-3, ClC-4, and ClC-5 [28]. In agreement with this hypothesis, practically all variants found here that lead to an apparent shift of the voltage-dependence to more positive voltages are located close to the dimer interface.

For data analysis of currents measured at various external pH values, the following leak-subtraction was performed. For each oocyte, currents measured at pH 7.3 were fitted in the range −120 mV <= V <= 0 mV with a straight line. The line was extrapolated to all voltages and subtracted from the current-voltage relationships (IVs) measured in the various conditions, and normalized to the current at pH 7.3, 160 mV. This is because for WT ClC-4 and for most variants, at pH 7.3, currents recorded at voltages V <= 0 mV are very small and indistinguishable from currents in un-injected oocytes and represent a mixture of leak and endogenous currents. Similar to the “voltage-shifted” variants, we interpret the emergence of inward currents at acidic as a partial disruption of the gating process that in WT keeps the transporter inactive at negative voltages, similar to what described for *CLCN3* variants [16]. Error bars in all figures represent SEM. Statistical significance was assessed by Student’s unpaired two-tailed *t*-test. Variance is similar between all groups because the same batches of oocytes were utilized for WT and variant measurements.

## Results

**Fig. 1:**
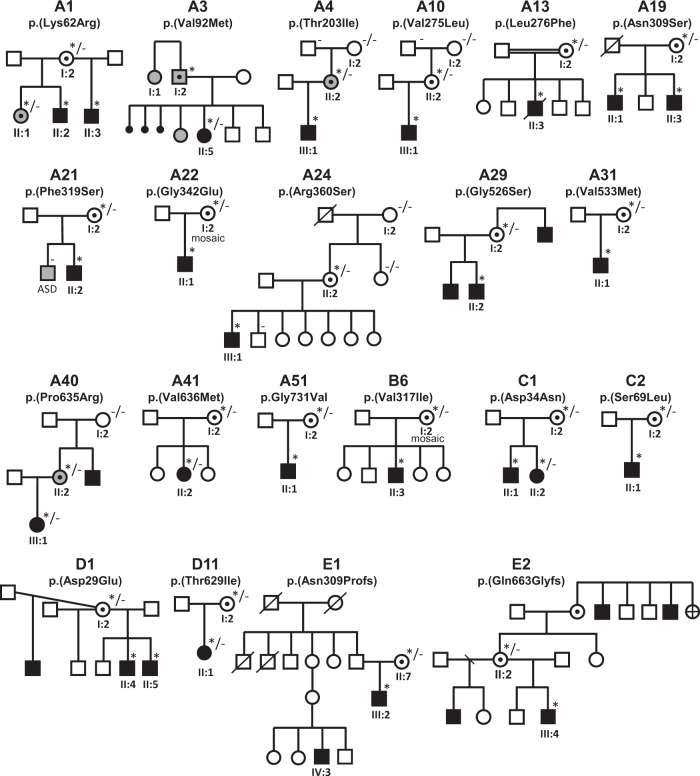
Pedigrees of all previously unreported families with inherited *CLCN4* variants. Filled square/circle = affected individual, lightly shaded circle/square = mildly affected individual, *familial *CLCN4 variant present in affected males, − familial *CLCN4* variant absent in male, */− familial *CLCN4* variant present in female, −/− familial *CLCN4* variant absent in female. Pedigrees of families with a *de novo* variant are not shown.

**Fig. 2:**
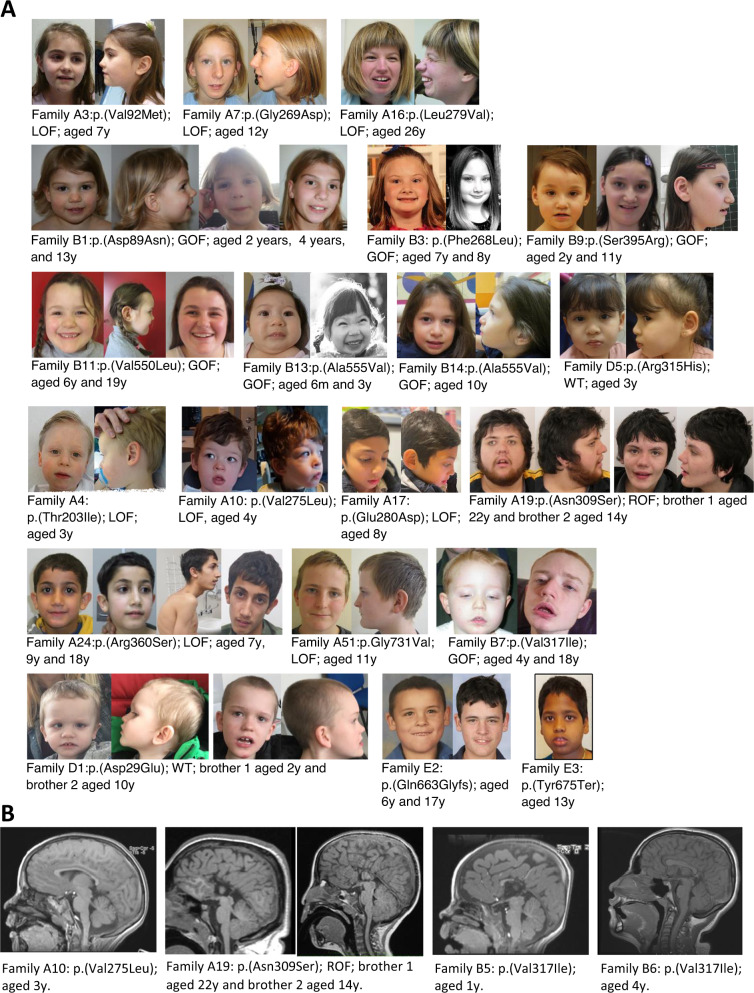
Clinical photographs of individuals with previously unreported variants in *CLCN4*, and representative neuroimaging. **A** Clinical photographs demonstrate that some males and females have progressive lengthening of their face and ‘squaring’ of the jaw with age. LOF loss-of-function, GOF gain-of-function, ROF reduction of function, m months, y years. **B** Neuroimaging (T1 mid-sagittal view) from affected probands. In all individuals there are abnormalities of the corpus callosum. The proband of Family A10 has a dysplastic corpus callosum: it is of normal length but globally hypoplastic. Family A19: two affected brothers both display complete agenesis of the corpus callosum with colpocephaly. Family B5: the proband has partial agenesis of the corpus callosum (affecting the posterior part and splenium), colpocephaly and mild dilatation of the 3rd ventricle. Family B6: the proband has a dysplastic corpus callosum, and mildly small optic chiasm and optic nerves bilaterally.

**Fig. 3:**
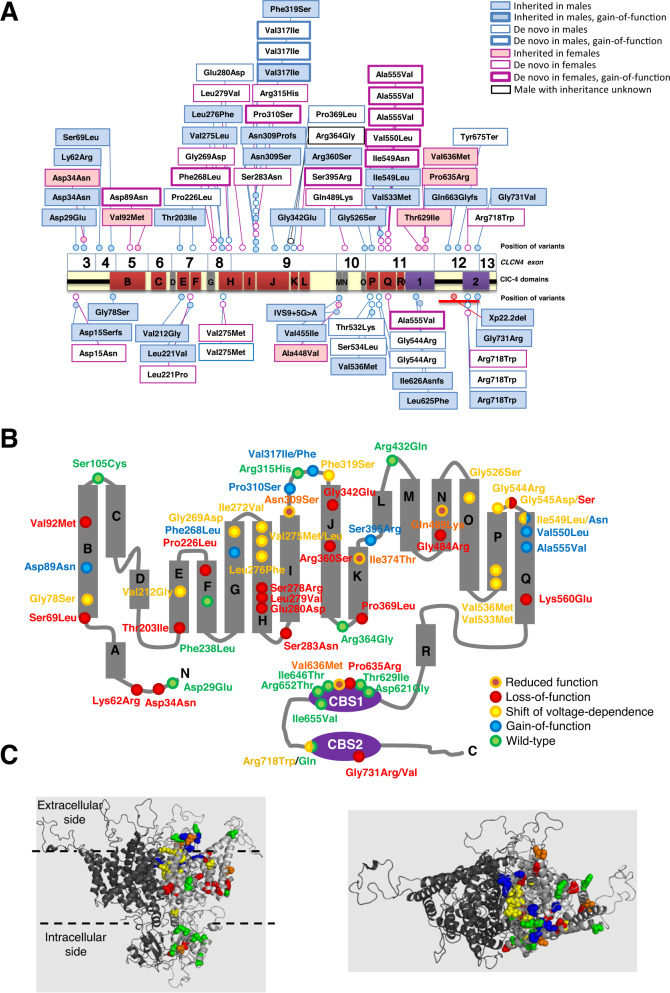
Mapping of all *CLCN4* variants functionally investigated in this study. **A** Schematic of the *CLCN4* gene and ClC4-protein with position of variants from newly identified families with clearly affected males and females depicted above the schematic, and position of variants published to date shown below the schematic. **B** Position of the investigated missense variants in a CLC topology model. Altered residues are shown as circles and functional effects are color-coded as indicated in the figure. **C** Three-dimensional homology model of the human ClC-4 protein based on the structure of the CmClC homodimer (Protein Data Bank: 3ORG). The view from within the membrane delimited by dashed lines. The two subunits forming the homodimer are shown in dark and light grey. Mutated residues are shown as spheres colored as in B. Right 3D model viewed from the extracellular site.

Detailed clinical data were analyzed on 55 previously unreported individuals, 22 hemizygous males and 33 female heterozygotes, from 43 previously unreported families, as well as updated clinical information on one previously reported female who was now recognized to have a recurrent variant [5]. The 44 families were divided into five groups (A-E). This includes families with missense variants, who were divided into groups A-D based on the functional results obtained in the *Xenopus laevis* oocyte model for the *CLCN4* missense variants as described below, as well as three additional patients with novel truncating variants (Group E). Demographic details of these affected individuals, the *CLCN4* variants, their frequency in the gnomAD database, in silico pathogenicity predictors, and results of in vitro functional studies in *Xenopus* oocytes are presented in Table 1. Table 1 also includes the details of variants from the public database ClinVar which we investigated with in vitro functional studies in *Xenopus* oocytes but where we were unable to obtain consent to publish clinical data, as well as variants from ClinVar (as of 25^th^ May 2022) and publications which were recurrent with our investigated variants. New ClinVar accession numbers were obtained for any variant with functional data not already listed in ClinVar and added to Table 1. Figure 1 shows the pedigrees of the unreported families with a novel inherited *CLCN4* variant previously unpublished in the peer-reviewed medical literature. Figure 2 shows clinical photographs and MRI brain images. Figure 3A is a schematic drawing of the *CLNC4* gene and ClC-4 protein with all variants of clearly affected males and females with clinical information available (this study and published). More details on the clinical presentation of these previously unreported families are detailed in Supplementary Table 1 and the case reports, and Supplementary Fig. 3 of individuals with blended phenotypes.

**Table 1:**
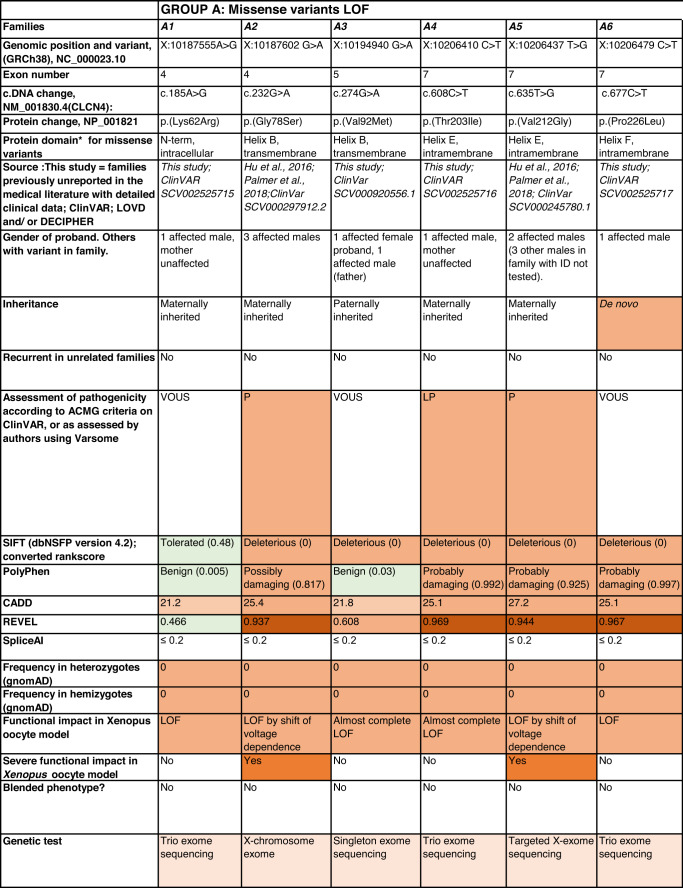

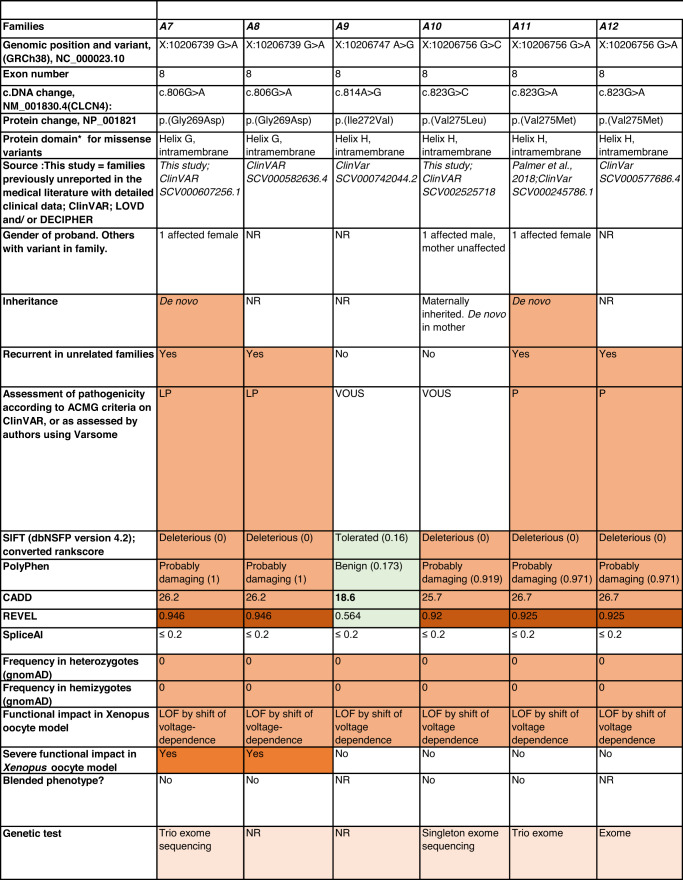

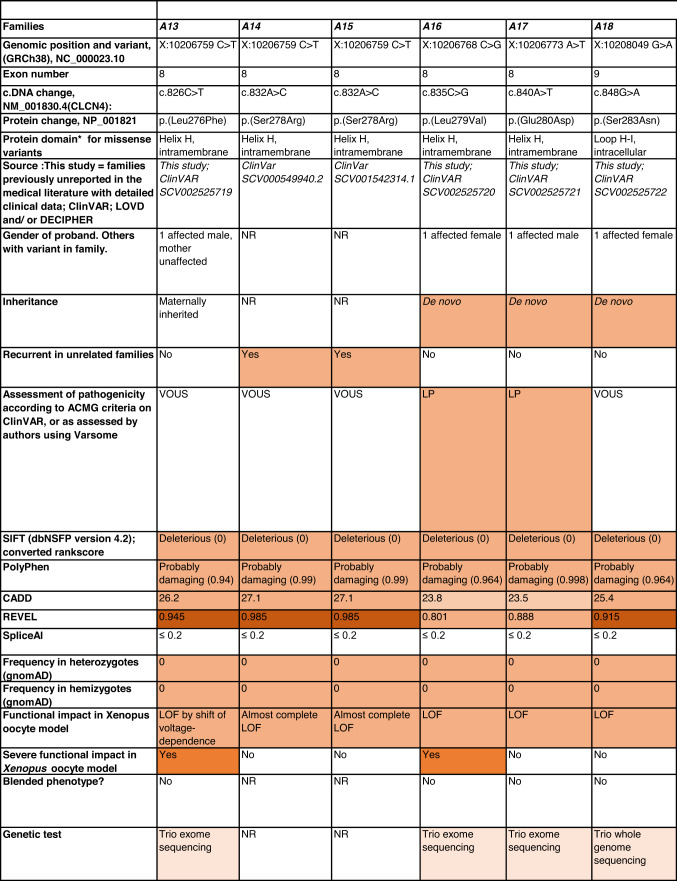

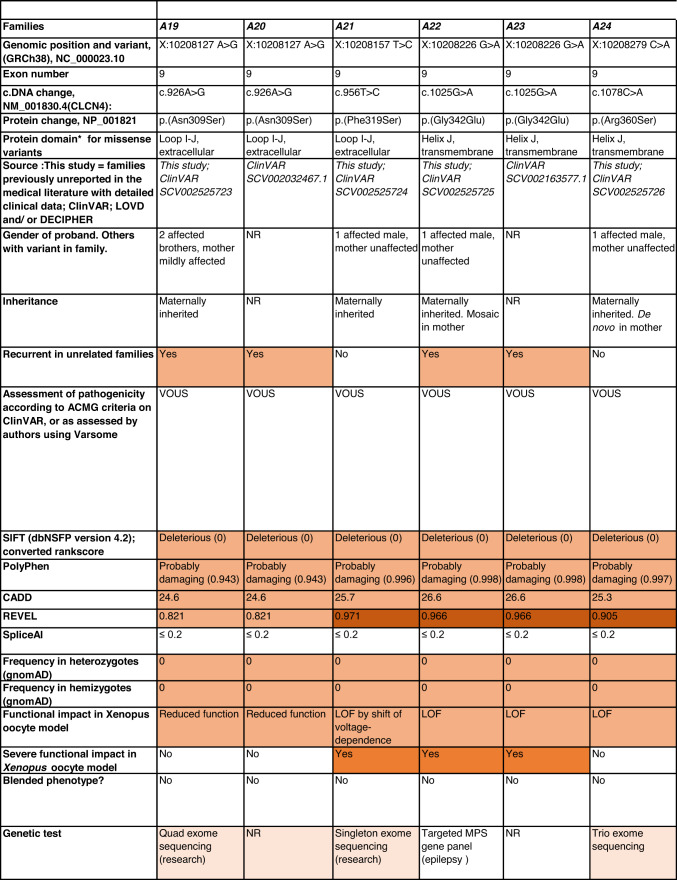

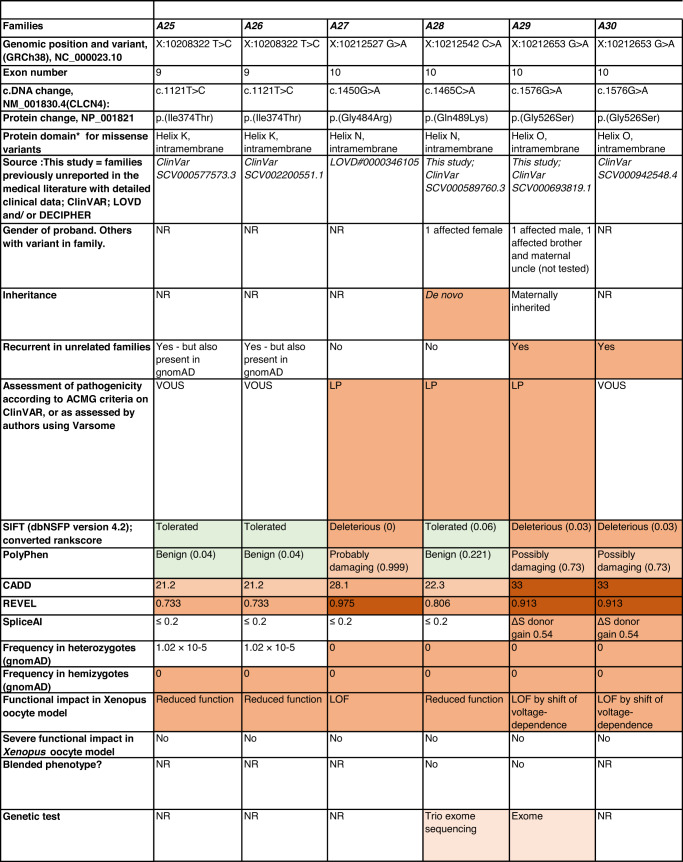

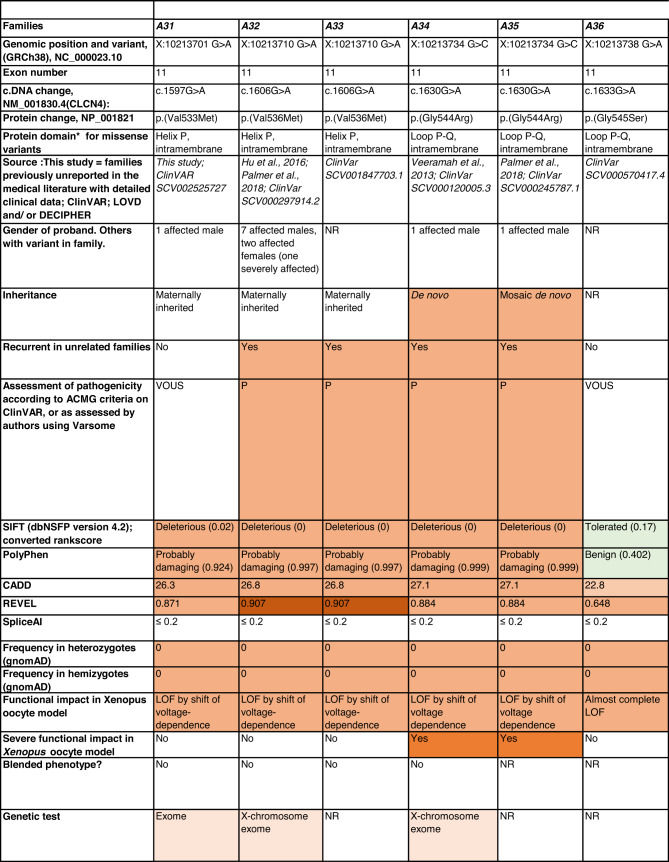

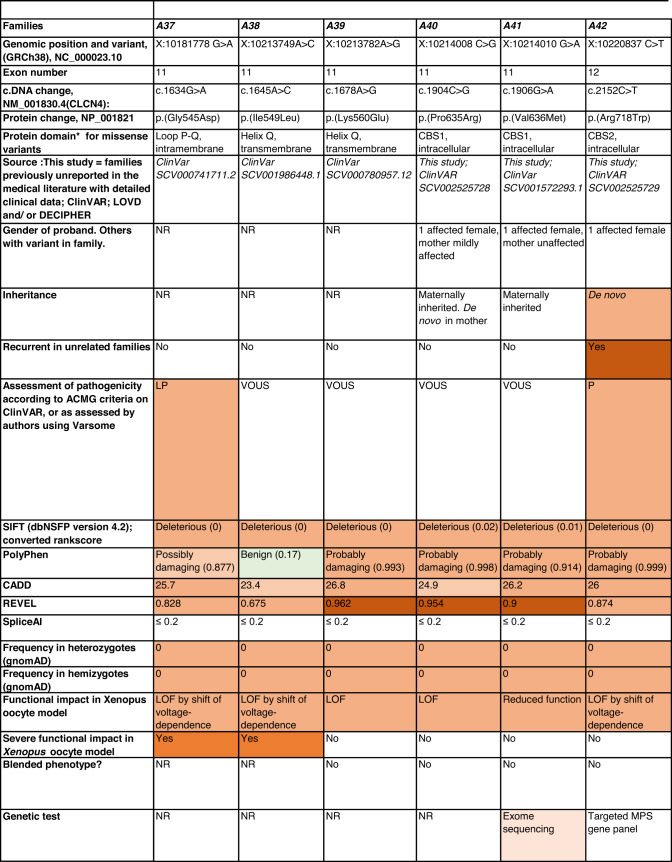

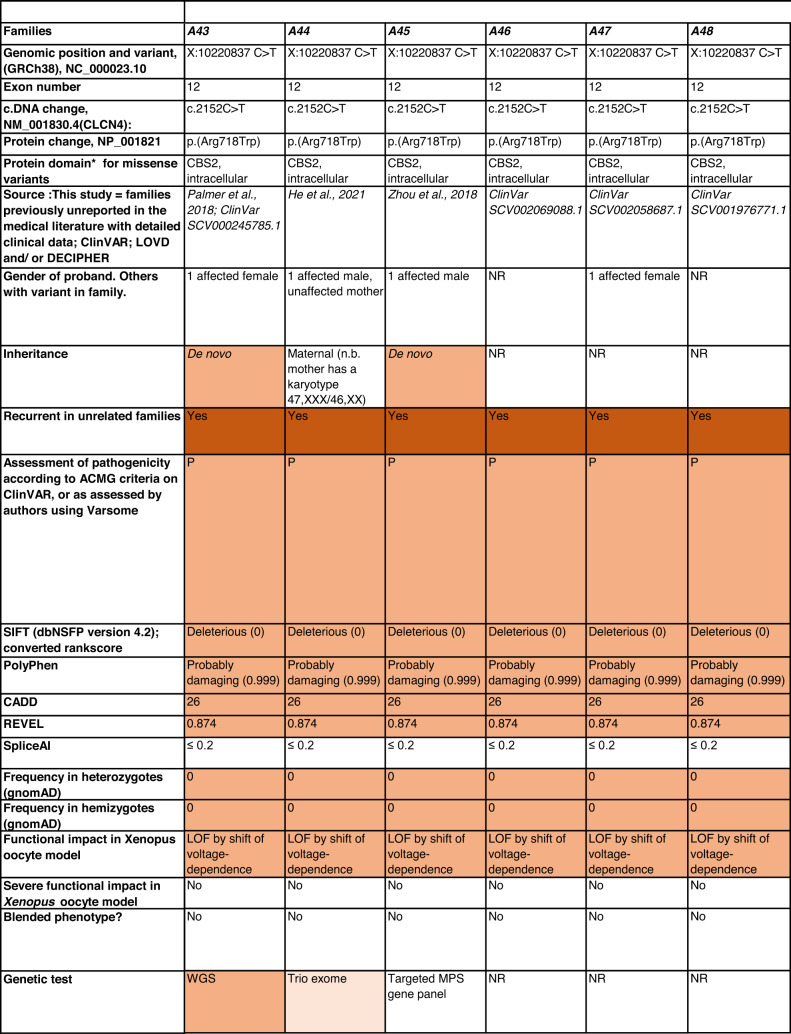

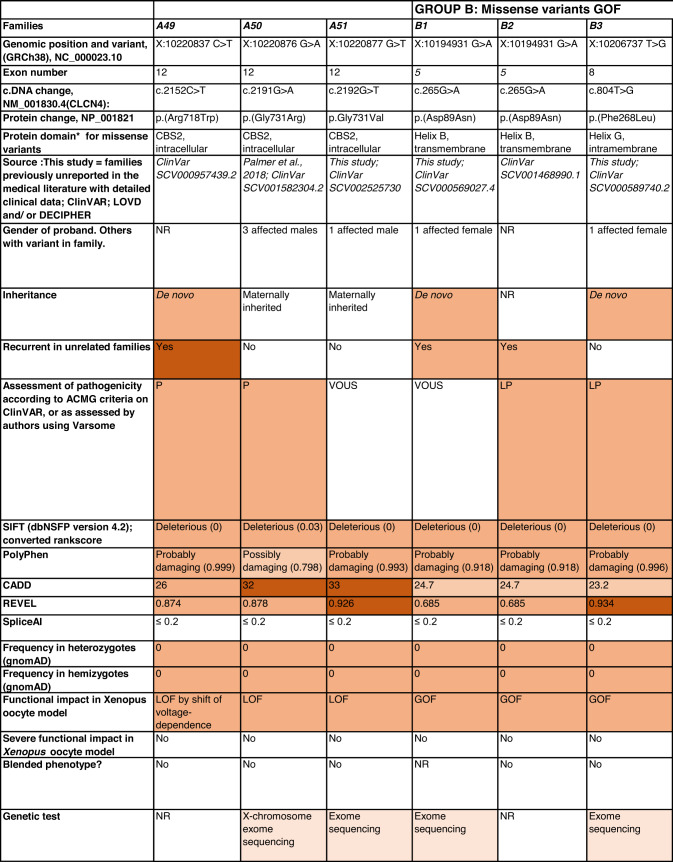

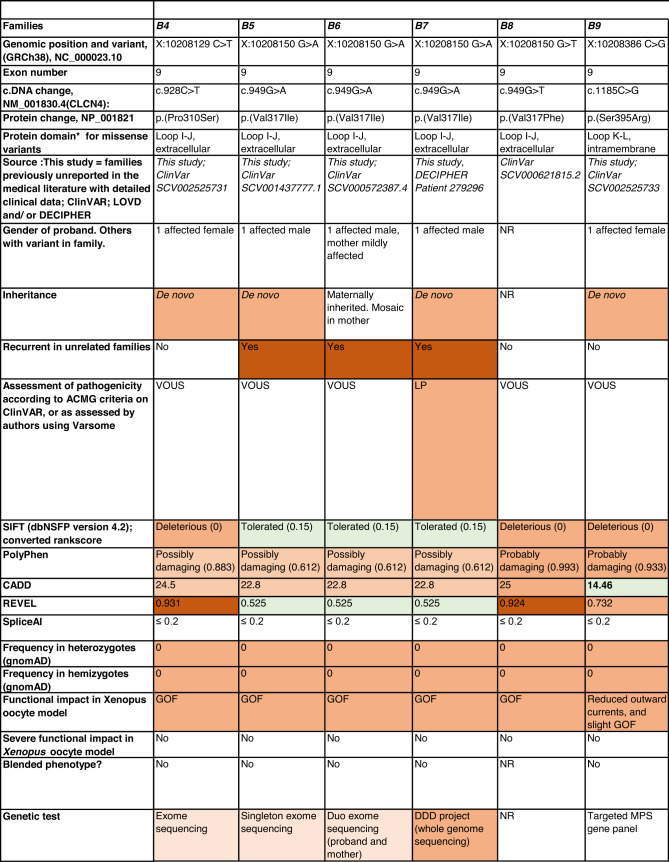

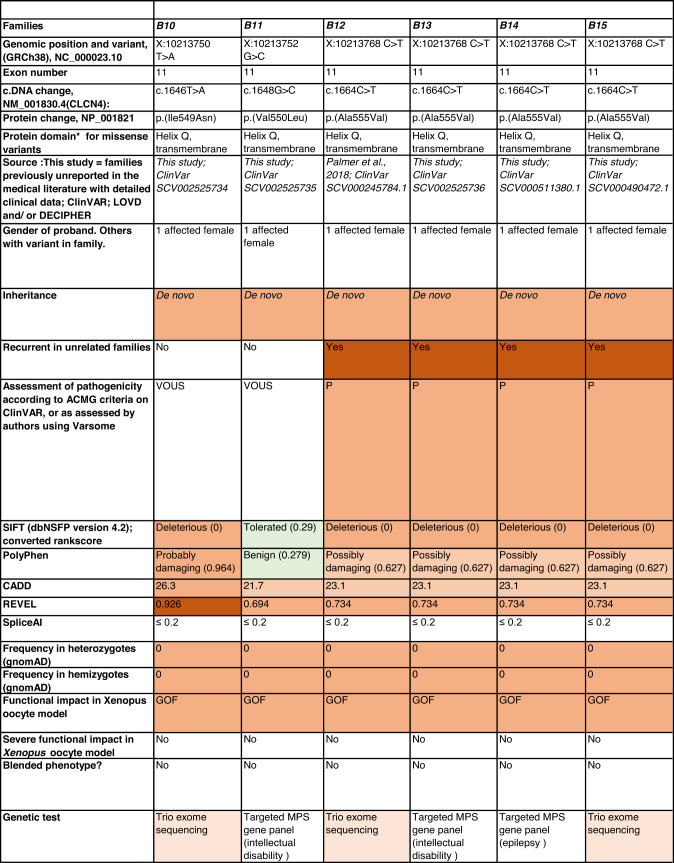

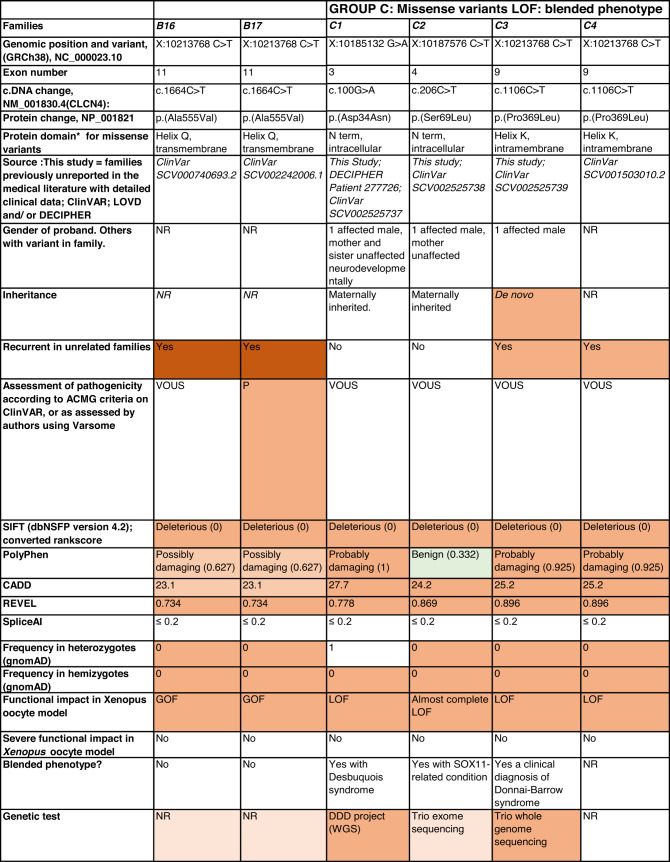

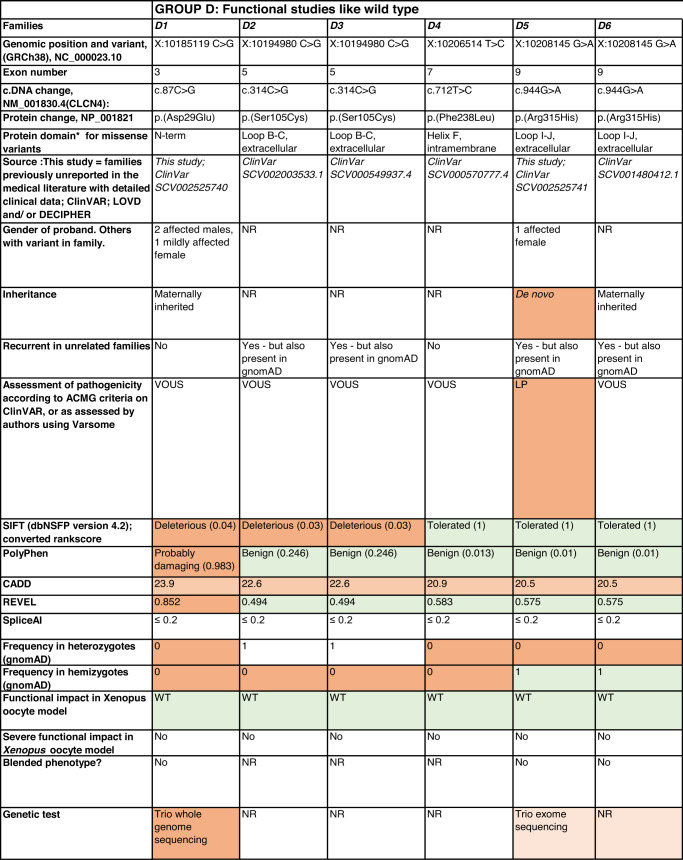

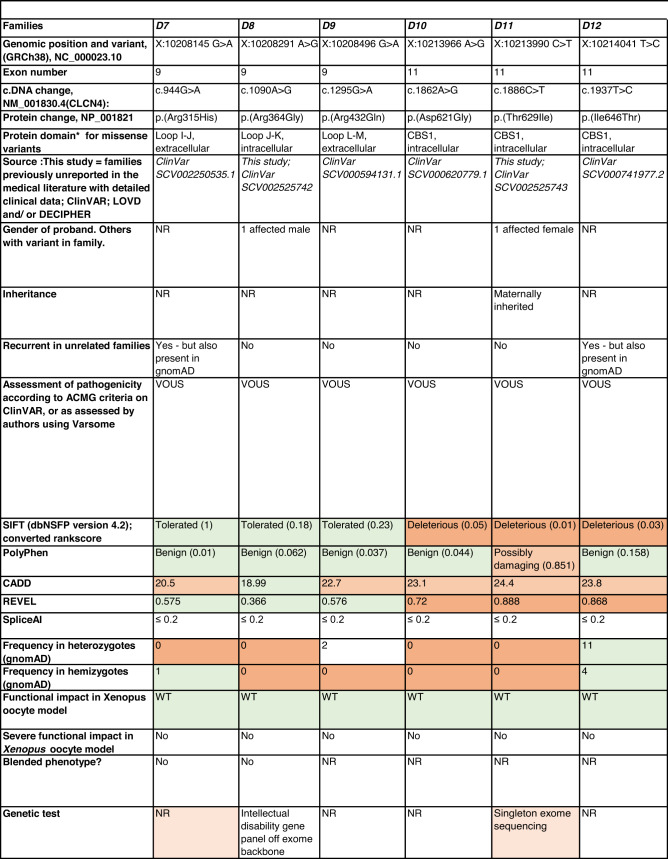

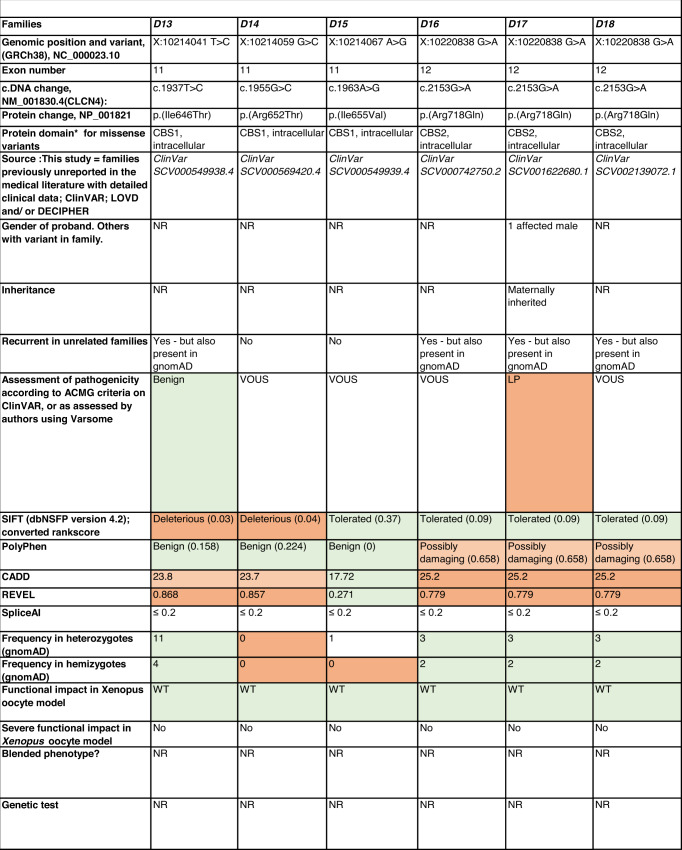

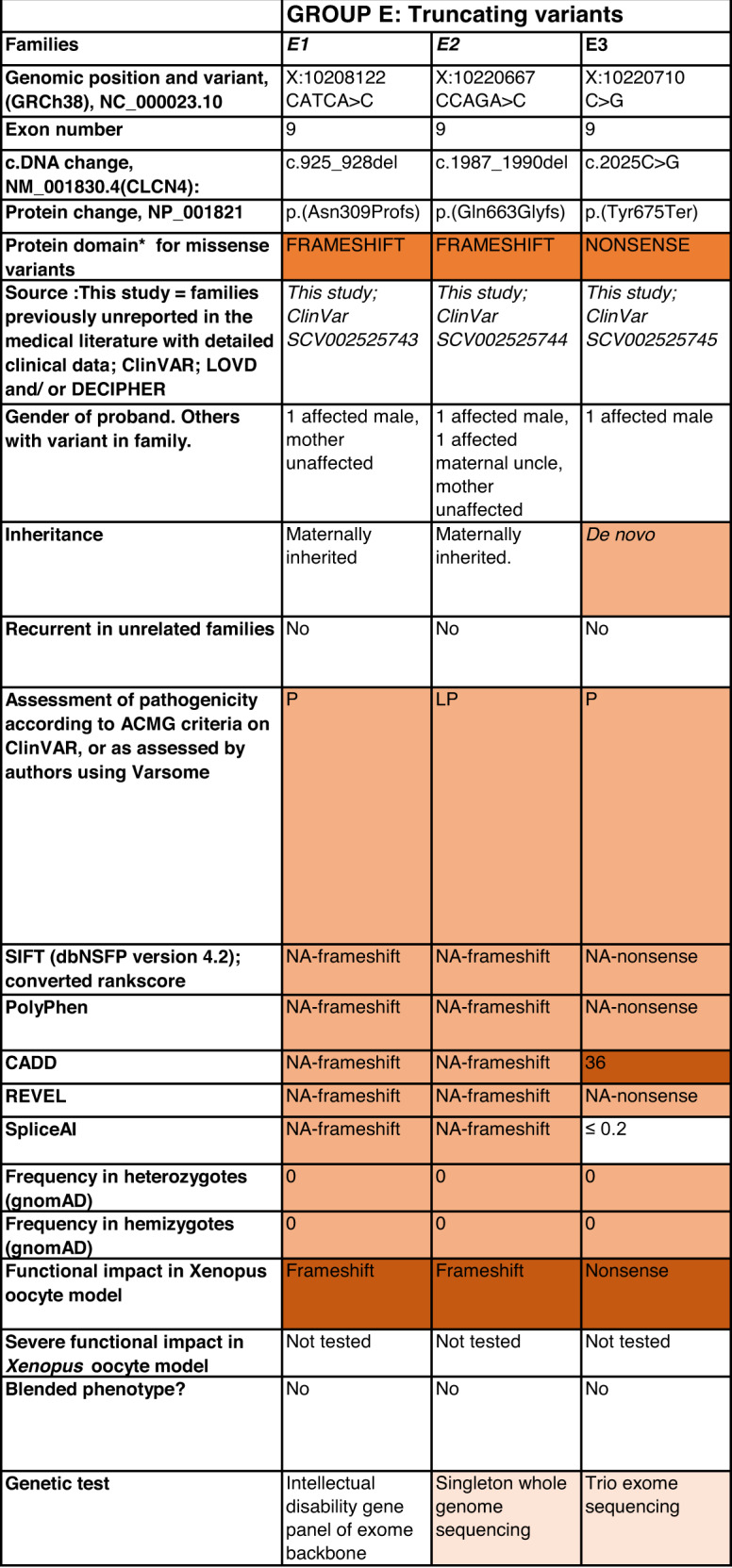
Summary of rare *CLCN4* variants reported in this study and, if recurrent, in previous literature or public databases.

Data presented include genomic coordinates, reporting laboratory assessment of pathogenicity using ACMG criteria as reported in ClinVar or determined by the authors using VARSOME prior to functional studies were conducted, demographic details, inheritance, recurrence within families, recurrence across families, including public databases as of 25th May 2022, selected in silico pathogenicity scores, frequency in gnomAD database, functional impact in *Xenopus* oocyte model, and if the individual has more than one genetic diagnosis (blended phenotype). Data which are supportive of pathogenicity is color-coded orange (with darker orange for most supportive data), data which are not supportive of pathogenicity are coded green. *ACMG* American College of Medical Genetics and Genomics, *CBS* cystathionine β-synthase, *NA* not applicable, *NR* not reported; *N-term* N terminus, *GOF* gain of function, *LOF* loss-of-function, *LOVD* Leiden Open Variation Database, *MPS* massively parallel sequencing, *WT* wild type. In silico scores include PolyPhen, CADD, REVEL and SpliceAI.

**Fig. 4:**
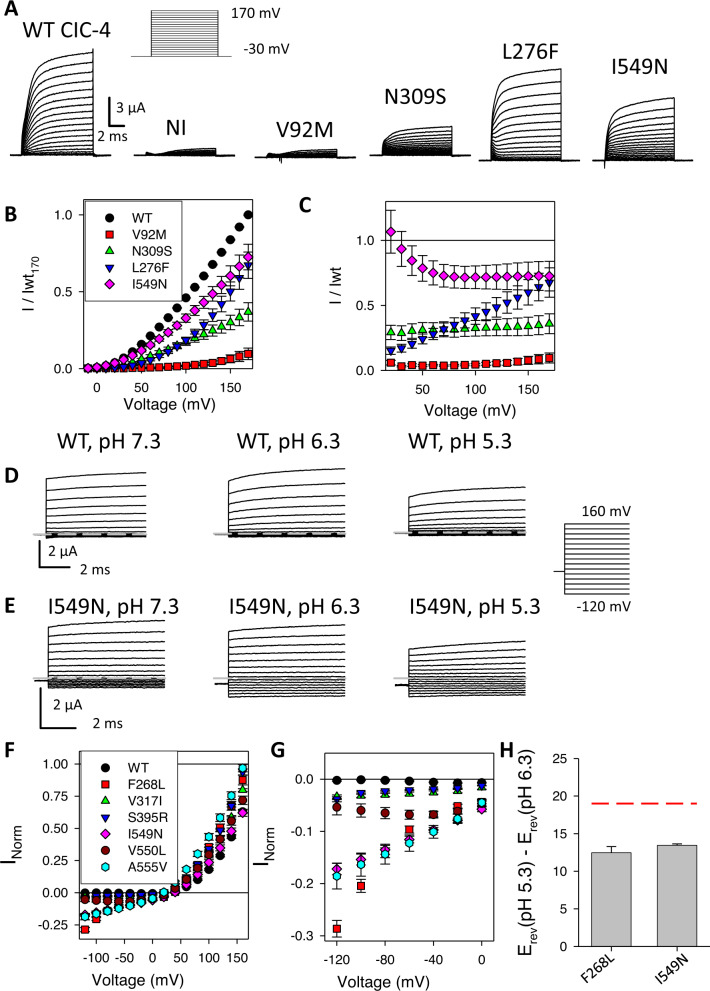
Expression of *CLCN4* variants in *Xenopus* oocytes. Panel **A** shows example recordings of the indicated constructs evoked by the voltage-clamp protocol indicated in the inset and using a “P/4” leak subtraction protocol (see Methods). Scale bars apply to all constructs. **B** shows average normalized IV relationships of the same variants. Currents are normalized to that of WT at 170 mV as described in Methods. In **C**, currents are normalized to the current of WT at the same voltage (see Methods). Data points are significantly different at practically all voltages from the value of 1 (i.e., WT) for all indicated variants (*p* < 0.05). Panel **D** shows typical current traces recorded without leak subtraction of WT ClC-4 in the presence of neutral and acidic extracellular pH, with outward currents being inhibited and inward currents remaining at a negligible level [29]. **E** illustrates the pH response of variant p.(Ile549Asn), which shows the activation of relatively large inward currents at acidic pH. **F, G** quantitative analysis of pH dependence of indicated GOF variants. Currents recorded at pH 5.3 were normalized to values measured at pH 7.3 as described in methods. The GOF effect of variants p.(Val317Ile) and p.(Ser395Arg) becomes apparent in panel **G** that shows the same data as panel F at a magnified scale. Data points are significantly different at voltages <= −40 mV from WT for all indicated variants (*p* < 0.05). Panel **H** shows differences in reversal potential measured at pH 6.3 and pH 5.3 for variants p.(Phe268Leu) and p.(Ile549Asn). The red line indicates the value expected for a stoichiometrically coupled 2 Cl^−^/1 H^+^ antiporter.

To test for possible impact of variants on the electrophysiological properties of the ClC-4 Cl^−^/H^+^ antiporter, missense changes as present in the affected individuals were introduced in ClC-4 expression constructs and studied by the 2-electrode voltage-clamp recording method in *Xenopus* oocytes. Example recordings are shown in Fig. 4A and all results in Table 1 and Supplementary Fig. 1. WT ClC-4 shows typical outwardly rectifying currents as described [29, 30]. An overview of all variants investigated functionally in this study are shown in a topology model and in a three-dimensional model in Fig. 3B, C.

Group A consists of 21 previously unreported families with detailed clinical data (see Supplementry Table 1) whose functional studies are part of this study and demonstrated a LOF, reduced function or shift of voltage dependence, as detailed below. These families included 13 with a male proband and eight with a female proband. In Table 1, Group A also includes seven families that we previously reported on with loss or reduced function [5]. It also includes data from 23 families whose variant was recurrent with one in our cohort, for whom only limited data was available in public databases (ClinVar, DECIPHER, LOVD) as of the 25th May 2022, or from publications from other groups [7, 8, 11, 31]. We included these families as further evidence of the pathogenicity of these recurrent variants.

Some variants, for example p.(Val92Met), showed current levels that were barely above those seen in un-injected oocytes (Fig. 4A–C). A similar near complete loss or reduced function was observed for other variants e.g., p.(Lys62Arg), p.(Ser278Arg), p.(Gly342Glu), and p.(Gly484Arg) (for full list see Table 1, Supplementary Fig. 1). As the variant p.Gly731Val affected the last amino acid of exon 12, we analyzed if this variant impacted splicing (Supplementary Fig. 2), but this could not be demonstrated. Little mechanistic insight can be obtained from these LOF variants as we did not analyze for example if protein stability was affected.

Other variants, for example p.(Asn309Ser), showed a reduced expression level, but no sign of altered voltage-dependence (Fig. 4A–C). The lack of altered voltage-dependence is highlighted in Fig. 4C, which shows that the ratio of currents mediated by variant p.(Asn309Ser) and currents of WT ClC-4 has a practically voltage-independent value of ~0.25. A similar, partially reduced function was observed for variants p.(Ile374Thr) and p.(Gln489Lys) (Table 1, Supplementary Fig. 1).

In contrast to the voltage-independent reduction seen in the variants described above, several other variants, including p.(Leu276Phe), showed a “right-shifted” voltage dependence. This is difficult to appreciate by just comparing the raw current traces (Fig. 4A) or the average current-voltage relationship (Fig. 4B) but is clear in Fig. 4C. For p.(Leu276Phe), the ratio of currents compared to WT is small for V ~ 20 mV but progressively enlarged at more positive voltages. The reduction of currents at “physiological” voltages is overcome by sufficiently large positive voltages. This essential LOF phenotype likely reflects an effect on the gating process of ClC-4, as detailed in Subjects and methods. Similar LOF by apparently right-shifted gating was observed to various degrees for variants p.(Gly78Ser), p.(Val212Gly), p.(Gly269Asp), p.(Ile272Val), p.(Val275Leu), p.(Val275Met), p.(Phe319Ser), and p.(Arg718Trp) (Table 1, Supplementary Fig. 1).

Group B. This group includes nine missense *CLCN4* variants from 17 independent families: 14 were previously unreported, one, a female with the *de novo* variant p.(Ala555Val), was previously reported by our group [5], and four families with the same variants or amino acid mutated, that were included in public databases but for whom we could not obtain detailed clinical data. These variants were grouped, as they showed compelling clinical evidence for pathogenicity (rarity, *de novo* status, matching clinical phenotype, and recurrence across unrelated families), without gross effect at the regular recording conditions at pH 7.3. However, p.(Ile549Asn) and some other variants exhibited a characteristic alteration (Fig. 4C): the ratio of currents compared to WT became progressively larger towards more negative voltages. This behavior is reminiscent of a GOF effect described for *CLCN3* variants [16]. Indeed, closer inspection of this and other variants revealed a dramatic GOF that is apparent particularly at acidic extracellular pH (Fig. 4E–G). While outward currents of WT ClC-4 were slightly inhibited at acidic pH and inward currents remained undetectable, comparably large inward currents became visible at pH 6.3 and 5.3 for variant p.(Ile549Asn) (Fig. 4D, E). A quantitative analysis revealed that similarly large inward currents were seen for variants p.(Phe268Leu) and p.(Ala555Val) (Fig. 4B, C). Smaller, but highly significant inward currents were also detected for variants p.(Asp89Asn), p.(Pro310Ser), p.(Val317Ile), p.(Val317Phe), p.(Ser395Arg), and p.(Ala550Leu) (Fig. 4F, G and Supplementary Fig. 1). Evidently, these variants partially disrupted the gating process of ClC-4 that normally prevents inward currents even at very acidic pH. For variants p.(Phe268Leu) and p.(Ile549Asn) inward currents were large enough to estimate reversal potentials at pH 6.3 and pH 5.3. The fact that the reversal potential in these conditions differed by about 12.5 mV for both variants (Fig. 4H) demonstrates that the inward currents carried by these variants are at least partially mediated by H^+^ transport. However, the difference falls short of the expected value of ~20 mV for a coupled 2Cl^−^/1H^+^ antiporter [32], suggesting that currents mediated by the variants are at least partially uncoupled. More detailed studies will however be needed to determine precise transport stoichiometry for these variants as well as for WT ClC-4.

Group C consisted of families with variants p.(Asp34Asn), p.(Ser69Leu) and p.(Pro369Leu). Although these variants all showed a functional LOF similar to those in Group A (Supplementary Fig. 1), the affected individuals had more complex clinical presentations (Supplementary Fig. 3) and an additional genetic condition was proven or strongly suspected, consistent with a blended phenotype. Consequently, they were separated from the other groups, and not included in the clinical summary in Table 2.

**Table 2:** Summarized clinical features, presented with HPO (Human Phenotype Ontology) nomenclature, of all individuals with a *CLCN4*-related neurodevelopmental condition from this study and from previous reports (in the case that detailed clinical data were available).

	Feature	HPO term	Males			Females							Males and females total
			All variants			*De novo* variants			Inherited or inheritance unknown variants			All variants	All variants
			Previously reported positive informative/total informative	This study positive informative/total informative	Total positive/ total informative	Previously reported positive informative/total informative	This study positive informative/total informative	Total positive/ total informative	Previously reported positive informative/total informative	This study positive informative/total informative	Total positive/ total informative	Total positive/ total informative	Total positive/ total informative
	**Unaffected**	HP:0032321	0/36 (0%)	0/22 (0%)	**0/58 (0%)**	0/5 (0%)	2/15 (13.3%)	**2/20 (10%)**	22/25 (88%)	13/19 (68.4%)	**35/44 (79.5%)**	**37/64 (57.8%)**	**37/122 (30.3%)**
**Neurodevelopment**	Borderline intellectual disability	HP:0006889	1/36 (2.8%)	0/22 (0%)	**1/58 (1.7%)**	1/5 (20%)	0/15 (0%)	**1/20 (5%)**	0/25 (0%)	0/19 (0%)	**0/44 (0%)**	**1/64 (1.6%)**	**2/122 (1.6%)**
	Mild intellectual disability	HP:0001256	9/36 (25%)	4/22 (18.2%)	**13/58 (22.4%)**	0/5 (0%)	6/15 (40%)	**6/20 (30%)**	1/25 (4%)	5/19 (26.3%)	**6/44 (13.6%)**	**12/64 (18.8%)**	**25/122 (20.5%)**
	Moderate intellectual disability	HP:0002342	9/36 (25%)	9/22 (41%)	**18/58 (31%)**	2/5 (40%)	5/15(33.3%)	**7/20 (35%)**	0/25 (0%)	1/19* (5.3%)	**1/44 (2.3%)**	**8/64 (12.5%)**	**26/122 (21.3%)**
	Severe/profound intellectual disability	HP:0010864	17/36 (47.2%)	7/22 (31.8%)	**24/58 (41.4%)**	2/5 (40%)	2/15 (13.3%)	**4/20 (20%)**	2/25 (8%)	0/19 (0%)	**2/44 (4.5%)**	**6/64 (9.4%)**	**30/122 (24.6%)**
	Specific learning disability	HP:0001328	0/36 (0%)	2/22 (9.1%)	**2/58 (3.4%)**	0/5 (0%)	1/15 (6.7%)	**1/20 (5%)**	0/25 (0%)	0/19 (0%)	**0/44 (0%)**	**1/64 (1.6%)**	**3/122 (2.5%)**
	Delayed speech and language	HP:0000750	36/36 (100%)	22/22 (100%)	**58/58 (100%)**	5/5 (100%)	14/15 (93.3%)	**19/20 (95%)**	NA	6/19 (31.6%)	**6/44 (13.6%)**	**25/64 (39%)**	**83/122 (68%)**
**Neurology**	Epilepsy	HP:0001250	22/36 (61.1%)	13/22 (59.1%)*	**35/58 (60.3%)**	2/5 (40%)	3/15* (20%)	**5/20 (25%)**	1/25 (4%)	1/19 (5.3%)	**2/44 (4.5%)**	**7/64 (10.9%)**	**42/122 (34.4%)**
	Well-controlled epilepsy	NA	8/22 (36.4%)	10/13 (77%)	**18/35 (51.4%)**	1/2 (50%)	3/3 (100%)	**4/5 (80%)**	0/1 (0%)	1/1 (100%)	**1/2 (50%)**	**5/7 (71.4%)**	**23/42 (54.8%)**
	Treatment-resistant epilepsy	NA	12/22 (54.5%)	3/13 (23.1%)	**15/35 (42.9%)**	1/2 (50%)	0/3 (0%)	**1/5 (20%)**	1/1 (100%)	0/1 (0%)	**1/2 (50%)**	**2/7 (28.6%)**	**17/42 (40.5%)**
	Information about seizure control not available	NA	2/22 (9.1%)	0/13 (0%)	**2/35 (5.7%)**	0/2 (0%)	0/3 (0%)	**0/5 (0%)**	0/1 (0%)	0/1 (0%)	**0/2 (0%)**	**0/7 (0%)**	**2/42 (4.8%)**
	Infantile hypotonia/ neonatal hypotonia	HP:0001252	11/36 (31%)	12/22 (54.5%)	**23/58 (39.6%)**	3/5 (60%)	8/15 (53.3%)	**11/20 (55%)**	1/18 (5.6%)	2/19 (10.5%)	**3/37 (8.1%)**	**14/57 (24.6%)**	**37/115 (32.2%)**
	Progressive neurological manifestations	HP:0001251; HP:0002191; HP:0001252; HP:0004305; ORPHA:279882	8/36 (22.2%)	7/22 (31.8%)	**15/58 (25.9%)**	1/5 (20%)	6/15 (40%)	**7/20 (35%)**	2/18 (11.1%)	1/19 (5.3%)	**3/37 (8.1%)**	**10/57 (17.5%)**	**25/115 (21.7%)**
	Abnormality of the brain	HP:0002363	14/18 (77.8%)	10/17 (58.8%)	**24/35 (68.6%)**	2/4 (50%)	8/13 (61.5%)	**10/17 (58.8%)**	1/1 (100%)	2/2 (100%)	**3/3 (100%)**	**13/20 (65%)**	**37/55 (67.3%)**
	Abnormality of white matter (e.g., white matter hyperintensities/ periventricular leukomalacia/ delayed or abnormal myelination)	HP:0002500	11/18 (61.1%)	9/17 (52.9%)	**20/35 (57.1%)**	2/4 (50%)	3/13 (23.1%)	**5/17 (29.4%)**	1/1 (100%)	0/2 (0%)	**1/3 (33.3%)**	**6/20 (30%)**	**26/55 (47.3%)**
	Abnormality of the corpus callosum	HP:0001273	6/18 (33%)	8/17 (47%)	**14/35 (40%)**	1/4 (25%)	3/13 (23.1%)	**4/17 (23.5%)**	1/1 (100%)	0/2 (0%)	**1/3 (33.3%)**	**5/20 (25%)**	**19/55 (34.5%)**
	Cerebral and/ or cerebellar atrophy	HP:0002059; HP:0007360	6/18 (33%)	2/17 (11.8%)	**8/35 (22.8%)**	2/4 (50%)	5/13 (38.5%)	**7/17 (41.2%)**	1/1 (100%)	0/2 (0%)	**1/3 (33.3%)**	**8/20 (40%)**	**16/55 (29.1%)**
	Other abnormality of the brain, e.g., Cortical dysplasia/ sclerosis/ cortical hyperintensities	HP:0002539	1/18 (5.5%)	1/17 (5.9%)	**2/35 (5.7%)**	1/4 (25%)	3/13 (23.1%)	**4/17 (23.5%)**	0/1 (0%)	2/2 (100%)	**2/3 (66.7%)**	**6/20 (30%)**	**8/55 (14.5%)**
**Psychiatry**	Autism spectrum disorder or autistic behavior	HP:0000729	2/36 (5.5%)	12/22 (54.5%)	**14/58 (24.1%)**	0/5 (0%)	6/15 (40%)	**6/20 (30%)**	1/18 (5.5%)	4/19 (21%)	**5/37 (13.5%)**	**11/57 (19.3%)**	**25/115 (21.7%)**
	Depression/ bipolar disorder	HP:0000716	4/36 (11.1%)	1/22 (4.5%)	**5/58 (8.6%)**	0/5 (0%)	1/15 (6.7%)	**1/20 (5%)**	1/18 (5.5%)	2/19 (10.5%)	**3/37 (8.1%)**	**4/57 (7%)**	**9/115 (7.8%)**
	Anxiety	HP:0000739	4/36 (11.1%)	6/22 (27.3%)	**10/58 (17.2%)**	1/5 (20%)	8/15 (53.3%)	**9/20 (45%)**	1/18 (5.5%)	2/19 (10.5%)	**3/37 (8.1%)**	**12/57 (21.1%)**	**22/115 (19.1%)**
	Obsessive and or compulsive behaviors	HP:0000722	2/36 (5.6%)	4/22 (18.2%)	**6/58 (10.3%)**	0/5 (0%)	2/15 (13.3%)	**2/20 (10%)**	0/18 (0%)	1/19 (5.3%)	**1/37 (2.7%)**	**3/57 (5.3%)**	**9/115 (7.8%)**
	Attention Deficit Hyperactivity Disorder/ or significant hyperactivity/ restlessness/ impulsivity	HP:0007018	4/36 (11.1%)	13/22 (59.1%)	**17/58 (29.3%)**	0/5 (0%)	7/15 (46.7%)	**7/20 (35%)**	0/18 (0%)	3/19 (15.8%)	**3/37 (8.1%)**	**10/57 (17.5%)**	**27/115 (23.5%)**
	Psychotic disorder	HP:0000709	0/36 (0%)	1/22 (4.5%)	**1/58 (1.7%)**	0/5 (0%)	0/15 (0%)	**0/20 (0%)**	0/18 (0%)	0/19 (0%)	**0/37 (0%)**	**0/57 (0%)**	**2/115 (1.7%)**
	Anger outbursts/ aggressive behavior	HP:0000718	8/36 (22.2%)	8/22 (36.4%)	**16/58 (27.6%)**	2/5 (40%)	4/15 (26.7%)	**6/20 (30%)**	0/18 (0%)	1/19 (5.3%)	**1/37 (2.7%)**	**7/57 (12.3%)**	**23/115 (20%)**
**Gastrointestinal and growth**	Gastroesophageal reflux	HP:0002020	1/36* (2.8%)	8/22 (36.4%)	**9/58 (15.5%)**	0/5 (0%)	5/15 (33.3%)	**5/20 (25%)**	0/18 (0%)	1/19 (5.3%)	**1/37 (2.7%)**	**6/57 (10.5%)**	**15/115 (13%)**
	Constipation	HP:0002019	0/36 (0%)	8/22 (36.4%)	**8/58 (13.8%)**	1/5 (20%)	6/15 (40%)	**7/20 (35%)**	0/18 (0%)	1/19 (5.3%)	**1/37 (2.7%)**	**8/57 (14%)**	**16/115 (13.9%)**
	Feeding difficulties	HP:0011968	2/36 (5.6%)	8/22 (36.4%)	**10/58 (17.2%)**	3/5 (60%)	8/15 (53.3%)	**11/20 (55%)**	0/18 (0%)	3/19 (15.8%)	**3/37 (8.1%)**	**14/57 (24.6%)**	**24/115 (20.9%)**
	Secondary microcephaly	HP:0005484	5/28 (17.8%)	4/20 (20%)	**9/48 (18.7%)**	2/5 (40%)	9/11 (81.8%)	**11/16 (68.7%)**	0/18 (0%)	1/19 (5.3%)	**1/37 (2.7%)**	**12/53 (22.6%)**	**21/115 (18.3%)**
	Failure to thrive	HP:0001508	2/27 (7.4%)	4/18 (22.2%)	**6/45 (13.3%)**	0/5 (0%)	5/13 (38.5%)	**5/18 (27.8%)**	0/18 (0%)	1/19 (5.3%)	**1/37 (2.7%)**	**6/55 (10.9%)**	**12/100 (12%)**
	Short stature	HP:0004322	1/27 (3.7%)	6/22 (27.2%)	**7/49 (14.2%)**	1/4 (20%)	4/13 (30.7%)	**5/17 (29.4%)**	0/18 (0%)	1/19 (5.3%)	**1/37 (2.7%)**	**6/54 (11.1%)**	**13/103 (12.6%)**
**Other**	Sleep disturbance	HP:0002360	0/36 (0%)	2/22 (9.1%)	**2/58 (3.4%)**	1/5 (20%)	3/13 (23.1%)	**4/18 (22.2%)**	0/18 (0%)	1/19 (5.3%)	**1/37 (2.7%)**	**5/55 (9.1%)**	**7/113 (6.2%)**
	Scoliosis/ kyphosis	HP: 0010674	3/36 (8.3%)	1/22 (4.5%)	**4/58 (6.9%)**	1/5 (20%)	0/13 (0%)	**1/18 (5.6%)**	0/18(0%)	0/19 (0%)	**0/37 (0%)**	**1/55 (1.8%)**	**5/113 (4.4%)**
	Other skeletal/ joint abnormalities	HP:0001763; HP:0030084; HP:0002829; HP:0001382; HP:0003298	1/36 (2.8%)	7/22 (31.8%)	**8/58 (13.8%)**	2/5 (40%)	1/13 (7.7%)	**3/18 (16.7%)**	0/18 (0%)	0/19 (0%)	**0/37 (0%)**	**3/55 (5.5%)**	**11/113 (9.7%)**
	Hearing impairment	HP:0000403; HP:0000407	0/36 (0%)	6/22 (27.3%)	**6/58 (10.3%)**	0/5 (0%)	2/15 (13.3%)	**2/18 (11.1%)**	0/18 (0%)	1/19 (5.3%)	**0/37 (0%)**	**2/55 (3.6%)**	**8/113 (7.1%)**
	Vision Impairment	HP:0000486; HP:0008058; HP:0000545; HP:0000505	0/36 (0%)	6/22 (27.3%)	**6/58 (10.3%)**	0/5 (0%)	3/18 (16.7%)	**3/18 (16.7%)**	0/18 (0%)	2/19 (10.5%)	**0/37 (0%)**	**3/55 (5.5%)**	**9/113 (8%)**

This table excludes patients with rare missense variants from Group D, for whom the functional studies were similar to wild type, and patients from Group C that had a more severe phenotype due to an additional monogenic condition.

Group D consisted of 18 families with rare missense variants with supportive in silico pathogenicity scores and/or clinical features suggestive of *CLCN4*-related condition, but for which no functional impact in the *Xenopus* expression system could be demonstrated. This group included variants p.(Asp29Glu), p.(Ser105Cys), p.(Phe238Leu), p.(Arg315His), p.(Arg364Gly), p.(Arg432Gln), as well as variants located in the intracellular CBS1 and CBS2 domains: the variants p.(Asp621Gly), p.(Thr629Ile), p.(Ile646Thr), p.(Arg652Thr), p.(Ile655Val), and p.(Arg718Gln) (Table 1, Supplementary Fig. 1). It is plausible that these variants are pathogenic by a mechanism not modelled in our cellular system, but given the lack of evidence on their pathogenicity, the families with Group D variants were not included in the summary Table 2.

Group E consisted of three individuals with a frameshift or nonsense variant in *CLCN4* for whom detailed clinical data were available.

This study thus brings the total number of individuals with (likely) pathogenic variants in *CLCN4* to a total of 122: 58 males and 64 females. For 20 of the females, parental studies demonstrated the variant to be *de novo*, while the other 44 females were identified as being heterozygous for a *CLCN4* variant only after a relative (usually a son, but on two occasions a daughter) was identified in their family to have *CLCN4*-related condition [1, 2, 5, 7, 11, 31]. The clinical features of this expanded cohort are summarized in Table 2.

## Discussion

Our study addresses the interpretation of novel missense variants, a common clinical conundrum across clinical genetic practice [33]. We robustly demonstrate a much wider range of functional impacts of *CLCN4* variants in the *Xenopus* oocyte model than had been previously demonstrated. In addition, we provide new insights into the common clinical features of *CLCN4-*related neurodevelopmental condition, which have enabled us to provide updated clinical management advice to clinicians [9] and improved patient and family education via the patient advocacy group CureCLCN4.

We confirm that cognitive disability is the most common clinical feature in males, most commonly in the moderate or severe/profound range (Table 2 and Supplementary Table 1). For the first time, however, we report a male with a verbal IQ in the normal range. This 12-year-old male (Family A21; p.(Phe319Ser)mat; functional studies: LOF by shifted voltage dependence) had a verbal IQ of 90 on formal psychometric testing (WISC-II-NL) but did have a lower performance IQ (61: within the mild ID range) and significant comorbidities with delayed language acquisition, articulation difficulties, severe treatment-resistant epilepsy, autistic features, and hyperactivity. We also report the first observation of a male with *CLCN4*-related condition and mild ID who has had a family (Family A3; p.(Val92Met); functional studies: almost complete LOF). He has two daughters who are obligate heterozygotes, one with mild ID and the other with specific learning disabilities.

Phenotypic prediction of cognitive function in females with *CLCN4*-related condition is very difficult with a wide spectrum of severity of neurodevelopmental and medical issues, including about half of heterozygous female carriers being apparently completely unaffected (Table 2). In general, females with a *de novo* variant had a more severe phenotype than those with an inherited variant. However, this observation is far from absolute, as evidenced by several female individuals in the cohort. For example, in families A10 and A24, the mother of a severely affected male had a *de novo* variant and yet was completely unaffected. On the other hand, we report females with inherited variants who have severe phenotypes: for example, the proband in Family A40 had moderate ID and a missense variant (p.(Pro635Arg); functional studies: LOF), which she inherited from a mother with mild ID. The proband had no additional genetic condition identified by WGS, and there was no evidence of mosaicism in the unaffected mother. We have previously reported that X-inactivation status does not correspond to clinical severity [5], and, as demonstrated across this and previous studies [8, 31], female-to-female inheritance from a very mildly, or even apparently non-affected mother does not ensure a mild phenotype in the daughter. Clinically, were a *de novo* missense *CLCN4* variant to be detected on a prenatal exome in a female embryo, there would remain a degree of uncertainty whether there would be a neurodevelopmental phenotype postnatally. From our previous study, it was apparent that females with a *CLCN4* frameshift or nonsense variant or a small intragenic chromosomal deletion of *CLCN4* are typically unaffected [5]. This was confirmed in the current study: both female carriers in the two families with inherited truncating variants in Group E were unaffected (Family E1 and E2). This observation may signify that the impact of missense variants in females could lead to a toxic gain-of function or a LOF that could be at least partially imparted also on ClC-3/ClC-4 heterodimers.

Behavioral and mental health disorders are the next most common clinical features. The four most common conditions were attention deficit hyperactivity disorder (ADHD) or significant hyperactivity, impulsiveness, or restlessness affecting 59% of all males and 46.7% of females with *de novo* variants; autism spectrum disorder (or autistic behavior) affecting 54.5% of all males and 40% of females with *de novo* variants; angry outbursts or challenging behaviors, affecting 36.4% of males and 26.7% of females, and lastly anxiety, affecting 27.2% of all males, 53% of females with *de novo* variants and 10.5% of females with inherited variants or variants with unknown inheritance. The mental health conditions were reported to significantly impact the affected individual’s ability to learn and their quality of life. Less frequent mental health disorders included obsessive compulsive disorder and depression/ bipolar disorder, which commonly had onset in late teenage years or early adulthood and caused a significant deterioration in quality of life. This highlights the need for close monitoring of all individuals for psychiatric complications, with appropriate referral to a psychiatrist skilled in the management of individuals with neurodevelopmental conditions.

Epilepsy is also confirmed as a significant feature of *CLCN4*-related neurodevelopmental condition, affecting 59% of all males and 20% of females. Most individuals with epilepsy had seizure onset within the first three years of life, although two were diagnosed at age 13, highlighting the need for ongoing seizure surveillance beyond childhood. Seizure semiologies were broad, including generalized absence and tonic-clonic seizures and focal onset seizures, as evidenced by EEG showing focal onset in some and generalized onset in others. Epilepsy can be severe, consistent with a developmental and epileptic encephalopathy as highlighted in recent reports [1, 11]. The severity of epilepsy, however, does not necessarily correlate with the severity of cognitive impairment. Due to *CLCN4* being an antiporter of protons and chloride, which may be important in acid-base balance, acetazolamide has been trialed without any clear evidence of improvement in seizure control. Indeed, no specific anti-seizure medications have been demonstrated to best correlate with epilepsy control.

Neuroimaging showed abnormalities in 58.8%% of males and 61.5% of females, most commonly of the white matter. Two brothers and one female had complete agenesis of the corpus callosum. This suggests that *CLCN4* should be added to panels of genes interrogated in individuals with corpus callosum abnormalities [34, 35].

Infantile hypotonia was reported in about half of all males and of females with *de novo* variants in this cohort. Progressive microcephaly was more common in females with *de novo* variants (80.8%) compared to males (20%). 31.8% of males and 40% of females with *de novo* variants had later onset neurological symptoms including tremor, ataxia, hyperkinesis or stereotypical movements, changes in gait such as walking with a stooped posture, or progressive spasticity.

Functional gastrointestinal symptoms, such as gastroesophageal reflux and constipation, were common in females with *de novo* variants (33.3% had gastroesophageal reflux and 40% had constipation) and impacted also a significant proportion of males (36.4% had gastroesophageal reflux and 36.4% had constipation). A small proportion of individuals, particularly those with GOF variants, have a striking growth phenotype. All four females with the recurrent *de novo* p.(Ala555Val) variant, for whom clinical data were available (Families B12-B15) had severe symmetrical growth restriction and feeding difficulties, two requiring gastrostomy feeds. The female proband from Family B13 was investigated by a pediatric endocrinologist, without evidence of growth hormone deficiency. The cause of this growth restriction requires further study but may reflect roles of the ClC-4 protein in fundamental growth processes or impact on enteric neurological function. Our findings underscore the importance of involving neurogastroenterology specialists in the comprehensive management of children with neurodevelopmental conditions, due to the significant impact on quality of life of underrecognized and untreated functional gastrointestinal comorbidities [36].

Other, less commonly noted clinical features include scoliosis, pes planus and/or lax joints, sleep disorders, otitis media with effusions, and strabismus. However, to date, our and other studies suggest that non-neurological congenital anomalies outside of the neurological system are not core features of *CLCN4*-related condition. With age, as previously described, there is a progressive lengthening of the face in males and females, with some males having a relatively ‘square’ jaw [5] (Fig. 2). Facial features in infancy and childhood are variable, without a recognizable ‘gestalt’.

With a larger cohort now functionally characterized, we examined whether distinct functional impacts of the ClC-4 variants correlated with phenotypic features. Some early observations could be made. Firstly, the GOF variants (Group B) were commonly associated with a severe growth, feeding and/or functional gastrointestinal component. Secondly, they had a higher female: male ratio; 73% of the affected individuals in Group A (LOF) were male, compared to only 41% in Group B (GOF). Thirdly, all three males with GOF variants had the same variant (p.(Val317Ile)): in two of these families the variant was *de novo*, in one maternally inherited. These males had similar clinical phenotypes including moderate to severe global developmental delay or ID, visual impairment (two were proven to have optic atrophy) and abnormalities of the corpus callosum. The functional impact of this variant was milder than that of the other GOF variants present in females. A possibility is that a severe GOF variant may not be compatible with life in a hemizygous male.

We cannot yet discount the pathogenicity of variants which performed like WT in our cellular model, as this is far from a complete model of the complexity of ClC-4 in animals in vivo, and, more specifically in the developing human. For example, variants that behaved like WT included the rare p.(Arg315His) *de novo* variant in a female (Family D5), who had clinical features entirely consistent with the spectrum seen in *CLCN4-*associated neurodevelopmental condition: however, this variant has also been reported in two other unrelated families in gnomAD. We also could not demonstrate a functional impact for several variants in the distal CBS domain, although it is possible that these variants may impact protein sorting or other mechanisms unable to be evaluated with the current *Xenopus* oocyte model.

In a structural model of a homodimeric ClC-4 protein, most variants characterized by a LOF with “rightward shifted voltage dependence” are localized at or near the dimer interface. This observation agrees with the hypothesis that voltage-dependent gating of ClC-4 is associated with a rearrangement of the dimer interface, as has been proposed for gating of the lysosomal ClC-7 [37]. Similarly, most GOF variants cluster at the dimer interface, mostly close to the luminal side. These mutants appear to partially destabilize the gate of the transporter that evidently must be tightly closed at negative voltages for proper function in endosomes. Interestingly, the isoleucine mutated in variant p.(Ile549Asn) (Family B10, severely affected female) that shows a particularly large GOF corresponds with Ile607 in the highly homologous ClC-3 protein; a variant at this position in *CLCN3* (p.(Ile607Thr)) similarly caused a dramatic GOF and the affected individual died within the first month of life. It is important to note that ClC-4 most likely forms heterodimeric complexes with ClC-3 [13]. Overall, the disease phenotypes caused by *CLCN3* and *CLCN4* variants are quite different, demonstrating that the two genes have overlapping but not identical functions. Our previous investigations on *CLCN4* missense variants which were found in heterozygous females did not support a potential dominant negative effect when equal amounts of WT and mutant ClC-4 were co-expressed in *Xenopus* oocytes [5]. However, the effect of voltage-gated shifted variants as well as GOF variants in heterodimeric ClC-3/ClC-4 complexes remains to be investigated [14]. Interestingly, the recurrent GOF variant p.(Tyr553Cys) in the late-endosomal ClC-6 causes a marked leftward-shift of the gating process [19, 38]. The corresponding tyrosine residue in ClC-4 is located just one residue away from Ile549. Both residues are in the linker connecting helices P and Q. The dramatic functional alterations of these variants provide additional evidence for a critical role of the linker P-Q in CLC transporter gating and corroborate the hypothesis that the GOF variants of vesicular CLCs are associated with a disrupted gating process.

We attempted to look at the possible impact of mosaicism on the phenotypic severity of *CLCN4* variants, but data are too scarce to robustly conclude that mosaicism for a *CLCN4* variant is predictive of phenotypic expression in females or males. This may be due to the lack of knowledge between the level of mosaicism in blood to that in the brain. For example, the variant p.(Arg718Trp), in the CBS2 domain, has now been reported *de novo* in both males and females with a severe phenotype (Table 1, Supplementary information), as well as in one unaffected mother, reported by He et al. [11]. However, we do note that this unaffected mother had a mosaic karyotype (47,XXX/46,XX) and it is possible that the ‘extra’ X chromosome may have somewhat moderated her phenotypic expression, as we considered for the unaffected male with Klinefelter syndrome, with an inherited *CLCN4* variant which resulted in a severe phenotype in his male relatives [5].

We report on four individuals (C1-C4) with a *de novo* or inherited missense *CLCN4* variant and supportive functional studies, but a more complex clinical phenotype, which we could attribute to a likely, or confirmed, blended genotype due to two monogenic conditions. For example, the male proband in Family C1 (p.(Asp34Asn)); whose functional studies were consistent with a LOF of ClC-4, has short stature and distinctive skeletal and facial features consistent with a diagnosis of Desbuquois dysplasia (*XYLT1*-related) that he shares with his sister. However, he has significant ID, epilepsy, and autism spectrum disorder, which are atypical for Desbuquois syndrome, and thus most likely has a blended phenotype of Desbuquois dysplasia and *CLCN4*-related neurodevelopmental condition. The finding of four patients with a blended phenotype due to suspected or proven multi-locus pathogenic variation in a total cohort of 122 individuals with *CLCN4* variants (4/122: 3.3%) is consistent with other studies estimating this phenomenon occurs in about 5% of individuals with an identified diagnosis after unbiased sequencing [39]. It emphasizes that for affected individuals, whose clinical features are not entirely consistent with their diagnosed monogenic condition, broadening the scope of genomic sequencing to an unbiased exome or whole genome sequencing approach may be appropriate to look for additional pathogenic findings

In summary, our study considerably expands our knowledge of the range of phenotypic and genotypic variation in *CLCN4*-related condition and for the first time robustly demonstrates a range of functional impacts, including gain of function. Variant classification still remains a nuanced art, rather than a precise science [40]. Fully informed genetic counselling is required to guide families through the diagnostic limitations and uncertainties inherent in genetic testing for neurodevelopmental conditions [41]. Several research priorities remain. We need to better ascertain the causality of all rare missense variants to elucidate targeted treatments. Establishment of a robust animal model is an urgent priority. This could potentially be a rat model, given that *Clcn4* is on the X chromosome in the rat, as opposed to in the mouse where it is autosomal [42]. A high throughput functional and therapeutic assay system, such as neuronal micro-electrode assays, which have been successfully applied in other neurodevelopmental conditions [43], would also be very helpful. With recent inclusion of *CLCN4* in the SFARI gene project [44] scientists and clinicians are working together to better understand and manage this condition.

## Supplementary information

Supplemental

Supplemental Table 1
