# Supplementary material for: Functional and clinical studies reveal pathophysiological complexity of CLCN4-related neurodevelopmental condition
Source: Mol Psychiatry. 2022 Nov 16;28(2):668–97. doi: 10.1038/s41380-022-01852-9 (PMC9908558; doi:10.1038/s41380-022-01852-9)
Supplement: Supplementary file 1 — Supplemental [file 41380_2022_1852_MOESM1_ESM.docx]

**Functional and clinical studies reveal pathophysiological complexity of *CLCN4*-related neurodevelopmental condition**

Elizabeth E Palmer^1,2,82^, Michael Pusch^3,82^, Alessandra Picollo^3^, Caitlin Forwood^1^, Matthew H Nguyen^2,4^, Vanessa Suckow^5^, Jessica Gibbons^5^, Alva Hoff^3,6^, Lisa Sigfrid^3,6^, Andre Megarbane^7,8^, Mathilde Nizon^9,10^, Benjamin Cogné^9,10^, Claire Beneteau^9^, Fowzan S Alkuraya^11^, Aziza Chedrawi^12^, Mais O Hashem^11^, Hannah Stamberger^13,14^, Sarah Weckhuysen^13,14,15^, Arnaud Vanlander^16^, Berten Ceulemans^17^, Sulekha Rajagopalan^4^, Kenneth Nunn^18^, Stéphanie Arpin^19^, Martine Raynaud^19^, Constance S Motter^20^, Catherine Ward-Melver^20^, Katrien Janssens^21^, Marije Meuwissen^21^, Diane Beysen^22^, Nicola Dikow^23^, Mona Grimmel^24^, Tobias B Haack^24^, Emma Clement^25^, Amy McTague^26,27^, David Hunt^28^, Sharron Townshend^29^, Michelle Ward^29^, Linda J Richards^30,31^, Cas Simons^32,33^, Gregory Costain^34^, Lucie Dupuis^34^, Roberto Mendoza-Londono^34^, Tracy Dudding-Byth^35,36^, Jackie Boyle^35^, Carol Saunders^37,38^, Emily Fleming^39^, Salima El Chehadeh^40,41^, Marie-Aude Spitz^42^, Amelie Piton^43^, Bénédicte Gerard^43^, Marie-Thérèse Abi Warde^42,44,^ Gillian Rea^45^, Caoimhe McKenna^45^, Sofia Douzgou^46,47^, Siddharth Banka^47,48^, Cigdem Akman^49^, Jennifer M Bain^49^, Tristan T Sands^49^, Golder N Wilson^50^, Erin J Silvertooth^51^, Lauren Miller^52^, Damien Lederer^53^, Rani Sachdev^1,2^, Rebecca Macintosh^1,2^, Olivier Monestier^53^, Deniz Karadurmus^53^, Felicity Collins^54^, Melissa Carter^55^, Luis Rohena^56,57^, Marjolein H Willemsen^58^, Charlotte W Ockeloen^58^, Rolph Pfundt^58^, Sanne D Kroft^59^, Michael Field^35^, Francisco ER Laranjeira^60^, Ana M Fortuna^61^, Ana R Soares^61^, Vincent Michaud^62,63^, Sophie Naudion^62^, Sailaja Golla^64^, David D Weaver^65^, Lynne M Bird^66^, Jennifer Friedman^66^, Virginia Clowes^67,68^, Shelagh Joss^69^, Laura Pölsler^70^, Philippe M Campeau^71^, Maria Blazo^72^, Emilia K Bijlsma^73^, Jill A Rosenfeld^74,75^, Christian Beetz^76^, Zöe Powis^77^, Kirsty McWalter^78^, Tracy Brandt^78^, Erin Torti^78^, [Mikaël](https://pubmed.ncbi.nlm.nih.gov/?sort=date&term=Mathot+M&cauthor_id=30716475) Mathot^79^, Shekeeb S Mohammad^18,80^, Ruth Armstrong^81^, Vera M Kalscheuer^5^

# Contents

[Contents 2](#_Toc104997880)

[Supplementary data: Case Descriptions 3](#_Toc104997881)

[Group A: (Likely) pathogenic: rare missense variants with functional studies in *Xenopus* oocytes consistent with a loss-of-function, and no clear-cut alternative genetic diagnosis. 3](#_Toc104997882)

[Group B: (Likely) pathogenic: rare missense variants with functional studies in *Xenopus* oocytes consistent with a gain-of-function, and no clear-cut alternative genetic diagnosis. 16](#_Toc104997883)

[Group C: Rare missense variant resulting in a loss-of-function on functional studies, with an additional confirmed or likely genetic diagnosis (blended phenotype) 24](#_Toc104997884)

[Group D Phenotype and *in silico* features suggestive of *CLCN4* encephalopathy but unable to confirm a functional impact. 27](#_Toc104997885)

[Group E: Truncating variants 30](#_Toc104997886)

[Supplementary Figure 1: Summary of electrophysiological results for all tested variants. 32](#_Toc104997888)

[Supplementary Figure 2: The novel single nucleotide variant present in proband and his mother from family A52 very likely does not affect splicing of *CLCN4* transcripts. 47](#_Toc104997889)

[Supplementary Figure 3: Clinical photographs of one affected male and one brother and sister from Group C with blended phenotype likely caused by another monogenic condition in addition to *CLCN4* missense change. 48](#_Toc104997890)

[REFERENCES 49](#_Toc104997891)

# Supplementary data: Case Descriptions

## Group A: (Likely) pathogenic: rare missense variants with functional studies in *Xenopus* oocytes consistent with a loss-of-function, and no clear-cut alternative genetic diagnosis.

**Family A1: NM_001830.4(*CLCN4*): c.185A>G; p.(Lys62Arg) maternally inherited**

This is a 14-year-old male born to Dutch parents. The mother is clinically unaffected but has two other children (half siblings to the proband) who both carry the *CLCN4* variant and are affected, a boy with severe autism and a girl with mild autism. Mother was 40 and the father 42 when the child was conceived. The pregnancy was complicated by maternal gestational diabetes controlled with insulin. Delivery was via induction of labor at 36 weeks with no perinatal complications. Apgar scores were 4 and 5. Birth weight was 3000 g (74^th^ centile). There was a one-week admission to the neonatal intensive care unit.

In infancy there was speech delay and walking occurred toward the end of the normal range at 18 months. Other clinical features include feeding difficulties, gastro-esophageal reflux, and constipation. He was diagnosed with epilepsy aged 2: mixed seizure semiology including absences, eye blinking, tonic-clonic seizures, and episodes can be associated with nausea, vomiting and tremor. Seizures occur more than 2 times a month and are not controlled with the current medication of ethosuximide. His electroencephalogram (EEG) showed focal discharges.

He has mild intellectual disability (FSIQ just above 70). He was diagnosed with autism spectrum disorder and ADHD. He has symptoms of hypokinesis and impulsiveness and is treated with pipamperone. There is no history of mania nor psychotic episodes.

He can feed orally. He can run independently but has difficulties with cycling due to difficulties with his balance. He can speak in sentences. He can dress and feed himself. Schooling is in a special class with allied health support including occupational therapy. He has had no developmental regression.

Growth measurements at age 13 showed head circumference 53.2cm (2^nd^-50^th^ centile), weight 31kg (0.7th centile; Z -2.41) length 139.5cm (1.5^th^ centile; Z-2.17). Other features on examination include high palate, macrodontia of the central incisors and restricted extension at the elbows.

Previously non diagnostic investigations included chromosomal microarray, urine metabolic screen, very long chain fatty acids, lysosomal enzymes, skin biopsy electron microscopy to consider for neuronal ceroid lipofuscinosis, mitochondrial DNA for common mutations and deletions/duplications, CSF lactate, amino acids, and neurotransmitters, *CDKL5* and *MECP2* sequencing and methylation at the Angelman locus.

Trio exome analysis identified a maternally inherited missense variant NM_001830.4(*CLCN4*):c.185A>G; p.(Lys62Arg).

**Family A3: NM_001830.4(*CLCN4*):c.274G>A; p.(Val92Met) paternally inherited**

The proband in this family is a 9-year-old girl, one of four children born to a French couple. She has a sister who is five years older and two brothers.

There were no prenatal complications. Delivery was at 40 weeks gestation with a birth weight of 2.355 kg (<10t^h^ centile) and length 46 cm (< 10^th^ centile). There were no postnatal complications.

There was an early onset of both gross and fine motor delay and speech delay. She walked independently at 15 months. Her first words were at 18 to 24 months. She can run currently. She receives speech and educational therapy and attends a special school. There has not been a regression of her skills.

She has a formal diagnosis of intellectual disability with features of hyperactivity but not a formal diagnosis of ADHD. Other features include impulsiveness, rigidity, and anger outbursts. There is no history of seizures, ataxia nor hypotonia. She has mild feeding difficulties and gastro-esophageal reflux.

At age seven her height was 1.135 m (- 1SD), weight 24.6 kg (0 SD), head circumference 49 cm (-1.5 SD). On examination she has synophrys and posteriorly rotated ears.

Her sister is five years older, currently aged 14 and has mild learning difficulties including dyslexia. She attends school with additional support and a modified educational program. Their father, who is 34 years old, had developmental delay and walked after 18 months. He has basic conversational communication skills and a formal diagnosis of mild intellectual disability. He attended special school. He has no formal qualifications although he does work in specialized employment. He can read and write with difficulties. Other medical issues in the father include a hand tremor and scoliosis. He is noted to have a long face, with squared chin. He is 182 cm tall and has a head circumference of 56 cm. There is a paternal aunt who has mild intellectual disability and has not had genetic investigations.

Singleton exome sequencing was done in the proband which found the following variant NM_001830.4(*CLCN4)*:c.274G>A; p.(Val92Met). On segregation this was found to be paternally inherited. The sister and aunt have not been tested. Other investigations with negative results included chromosomal microarray and *FMR1* repeat analysis.

**Family A4: NM_001830.4(*CLCN4*): c.608C>T; p. (Thr203Ile) maternally inherited**

This is a 3-year-old male child born to Caucasian parents. He was born at 38 gestational weeks after an uncomplicated pregnancy with a birth weight of 2.64 kg. After birth, difficulties establishing suck feeds was noted. There were no other postnatal complications. At the age of 4 months, physiotherapy was started because of hypotonia. He was described as a ‘passive’ baby. His motor development was delayed with sitting at 10.5 months and walking at 26 months. His speech development was also delayed. At the age of 3 years, there was no expressive language. At 4 years and 4 months, he speaks some words and is learning to use sign language.

At 20 months a Bailey’s assessment was consistent with him having a cognitive developmental level at 7 months (developmental index of 55). He has been diagnosed with a mild intellectual disability. He has poor concentration skills; he has difficulties to focus when playing with toys. There are no signs of epilepsy.

He was also diagnosed with autism spectrum disorder with repetitive movements at the age of (approximately) 2 years.

On clinical examination at the age of 2 years and 8 months he had a generalized hypotonia and a broad-based gait. Growth parameters were of height 91.0 cm (3-10^th^ centile); weight 10.3 kg (<3^rd^ centile) and head circumference 45.6 cm (<3^rd^ centile). Other examination findings include mild malar flatness, a long philtrum, and a left-sided single palmar crease.

Brain MRI shows corpus callosum hypoplasia but otherwise no abnormalities.

His mother had a slightly delayed motor development, walking at 19 months. She was diagnosed with autism spectrum disorder. She received speech therapy and physiotherapy as a child. She had learning difficulties and needed special education. As an adult she has been diagnosed with depressive illness twice. She can function independently. Neither of her parents have developmental or learning difficulties.

Exome sequencing detected a maternally inherited missense variant: NM_001830.4(*CLCN4)*:c.608C>T; p.(Thr203Ile). The variant is absent in the maternal grandparents of the patient, demonstrating *de novo* occurrence in the mother of our patient.

Chromosome microarray [Hg19] identified a small (360 kb) 3q25.32 deletion that was paternally inherited and was classified as a VOUS. The deletion encompasses *GFM1,* pathogenic variants in which are associated with autosomal recessively inherited Combined Oxidative Phosphorylation Deficiency 1. No second variant was identified in *GFM1* on exome sequencing.

**Family A6: NM_001830.4(*CLCN4*):c.677C>T; p.(Pro226Leu) *de novo***

This is a 20-year-old Turkish male with moderate intellectual disability and speech delay. His father was 41 and mother 34 years old at conception. He has autism spectrum disorder and aggressive behaviors. He has some degree of motor ‘clumsiness’ and a tremor exacerbated by anxiety. He also has vesico-ureteric reflux and allergies.

He was born at term after an uncomplicated pregnancy, but delivery was complicated by meconium aspiration. He required observation for one day in the special care nursery. It is not known if he had infantile hypotonia, but there was global developmental delay: he walked at 3.5 years and his first words were at 54 months. As an adult he can speak in sentences, sing children’s songs and can do simple reading and writing, as well as dress and feed himself. He has been assessed as having a moderate intellectual disability with a FSIQ of 35-40. There are concerns that he had stagnation or regression of his development at the age of 16 years, which does not clearly relate to the onset of seizures. He attended a special educational unit with care provided in a tertiary epilepsy center. He has a formal diagnosis of autism.

He has a severe drug-resistant epilepsy, with frequency of seizures that can vary from 3 a day to once every 3 months. First seizures were at age 9 years and were generalized tonic-clonic in nature. He has also had absence seizures. Sometimes his seizures have been provoked by fevers. Multiple antiepileptic drugs have been trialed and he is currently treated with phenytoin, clobazam and oxcarbazepine. It is possible that sodium valproate may have exacerbated his seizures, this was discontinued. He currently has a vagal nerve stimulator. The EEG features show multifocal epileptiform discharges. MRI brain has been normal. There are no mental health diagnoses, but he occasionally can be aggressive, possibly as a side-effect of his antiepileptic medication. He has a degree of motor ‘clumsiness’ and has a tremor when he is nervous. Other medical complications include vesicoureteral reflux, and allergies. He feeds orally.

At 18 years of age his height was 178 cm (50^th^-75^th^ centile). There was no evident dysmorphism.

Exome sequencing identified a *de novo* missense variant NM_001830.4(*CLCN4)*:c.677C>T; p.(Pro226Leu). A variant of uncertain significance in *SCN8A* was previously detected on an epilepsy gene panel (c.3272A>G; p.(Asn1091Ser), which was paternally inherited. There is no paternal history of epilepsy, and this was not thought to be causative.

**Family A7: NM_001830.4(*CLCN4)*:c.806G>A, p.(Gly269Asp) *de novo***

The proband is a 15-year-old Caucasian Canadian female.

There was a high-risk antenatal screen for trisomy 21 (1:35). Birth weight was 2.95 kg at 41 weeks (3-10^th^ centile). She had infantile hypotonia.

There was global developmental delay. She walked at 13 months. Her first words were at 2.5 years. She has no current mobility restrictions but mild coordination difficulties. She can speak in sentences. Her reading ability is at a grade 1 level. There has been no formal psychometric assessment, but she functions at a level consistent with mild intellectual disability. She attends a special education class. She has no behavioral issues. She does have mild anxiety.

She has a spina bifida occulta at the L5 vertebral level. She had obstructive sleep apnea requiring adenotonsillectomy.

Feeding was established orally but she has required a gastrostomy tube for feeding difficulties and reflux from the age 3.5years.

On most recent examination at 12 years of age she was symmetrically small: her height was just above the first centile at 135.5 cm (Z= -2.4), weight at 25.4 kg significantly below the 3^rd^ centile (Z= -2.7) and head circumference significantly below the 2^nd^ centile at 47.4 cm (Z = -4). She has subtle dysmorphic features including an elongated and narrow face, sloping forehead, prominent nose with high nasal bridge, long philtrum, thin upper lip vermillion, micrognathia, high arched palate, dental crowding, and bilateral 5th finger clinodactyly.

MRI brain at age 2 years showed mild prominence of the lateral ventricles with septation through the right lateral ventricle at the base of the frontal horn.

Investigations include a normal chromosome microarray age 11 and primary microcephaly multi-gene panel (17 genes) age 9 which were non diagnostic. Exome sequencing identified a *de novo variant* NM_001830.4(*CLCN4*):c.806G>A; p.(Gly269Asp).

**Family A10: NM_001830.4(*CLCN4*):c.823G>C; p.(Val275Leu) maternally inherited**

The proband is a 4 year 9-month-old male of Caucasian heritage. There is no family history of intellectual disability.

He was born at term after an uncomplicated pregnancy. Birth weight was 3.82kg (50-85^th^ centile); length 51.5cm (85^th^ centile) and head circumference 35 cm (50^th^ centile) Apgar scores were 10 at 1 and 5 minutes. There were concerns with his development in the first year of life with gross and fine motor delay, speech delay. He had significant early and global hypotonia. He sat independently at 14 months. He walked independently at 4.5 years. He still has no verbal communication other than ‘yes’. He is starting to communicate with his hands a little. There were concerns with significant developmental regression at 4 months. He has not received a formal diagnosis of intellectual disability but clinically is judged to function in the severe range of intellectual disability. He has a short attention span. He does not have a diagnosis on the autism spectrum or any mental health diagnoses.

He has epilepsy with first seizures being absences at the age of 4 months, followed by infantile spasms at the age of 6 months, evolving to focal epilepsy. He has been seizure free since the age of 2.5 years on a single agent: levetiracetam. Previously trialed antiepileptics include hydrocortisone, sodium valproate, vigabatrin and topiramate.

He is orally fed and has no significant gastrointestinal symptoms. On examination his most recent height (4 years 9 months) was 108cm (90^th^ centile), weight 19.3kg (90^th^ centile) and head circumference 51 cm (75^th^ centile). He has an elongated face, facial hypotonia with an open mouth, full cheeks and micrognathia.

MRI brain showed a completely myelinated corpus callosum, of normal length but globally hypoplastic consistent with dysgenesis of the corpus callosum

Other genetic investigations included a normal chromosomal microarray and gene panel. Exome sequencing demonstrated a maternally inherited variant NM_001830.4(*CLCN4*):c.823G>C; p.(Val275Leu). Further studies demonstrated that this variant was *de novo* in the mother.

**Family A13: NM_001830.4(*CLCN4*):c.826C>T; p.(Leu276Phe) maternally inherited**

The proband was a 3-year-5-month-old male, the 3rd child born to consanguineous Arabic parents.

His birth weight was 2.4kg (other measurements not known). There were no immediate postnatal complications however he had profound global developmental delay. He was non-verbal with delayed motor skills at a 6-month level (he was not able to roll or sit unsupported). Formal psychometric assessment had not been attempted. There is no history of ADHD or autism.

Seizures developed at age 2 months and were generalized tonic-clonic in nature. They occurred weekly and were of short duration (less than 1 minute). Trialed medications included clobazam, levetiracetam and phenobarbitone. EEG demonstrated diffuse suppression consistent with generalized nonspecific cerebral dysfunction, without epileptiform activity. MRI scan of the brain showed severe cerebral and cerebellar atrophy with thinning of the corpus callosum, mild atrophy of bilateral thalami, abnormal cerebral white matter signal and mild bifrontal cerebral collections.

At 3 years of age his height was 90.5cm (10^th^ centile), weight 13.1kg (10-25^th^ centile) and head circumference 42cm (<< 2^nd^ centile). He was dysmorphic with central hypotonia and peripheral spasticity. He has 2 large hyperpigmented lesions on the trunk. Ophthalmological exam was normal.

Other medical issues included constipation and gastro-esophageal reflux. He was fed via gastrostomy tube with bolus feeds. There is no history of cardiac, renal, or pulmonary disease.

Other previous investigations include a normal chromosomal microarray, metabolic screen and leukodystrophy panel.

On exome sequencing he had a maternally inherited NM_001830.4(*CLCN4*):c.826C>T; p.(Leu276Phe) variant identified.

He died at the age of 4 years following an infection.

**Family A16: NM_001830.4(*CLCN4*):c.835C>G;** **p.(Leu279Val)** ***de novo***

The proband is a 39-year-old female with a moderate developmental delay with autistic features and significant anxiety. She is the third of four siblings to non-consanguineous parents. There was no history of other family members with delayed or abnormal development. She was born at term via a vaginal delivery after an uncomplicated pregnancy. Her birth weight was 3.6 kg and other birth parameters were recalled as being normal. She fed well in the newborn period and developed normally in the first year of life.

Concerns were raised about delayed speech development at 20 months of age. Her speech was limited into adolescence, and she now communicates in short sentences. She had recurrent atypical and recurrent tonic- clonic convulsions associated with fevers from 13 months to 7 years of age. These were treated with carbamazepine. She is now seizure free on no anti-epileptic medications.

She has repetitive motor mannerism, restricted interests, perseverative speech, and hyperactivity and was diagnosed with an autism spectrum disorder. Her anxiety features now manifest during prolonged separation from her parents with short lived catatonic episodes where she is unable to sleep, has language regression and becomes incontinent of urine and feces. Her anxiety has been generally resistant to medical management but helped with a behavioral support plan. She works in supported employment and lives in a group home during the week.

Examination in adulthood revealed an OFC of 53.5cm (10^th^ centile) height 166cm (75^th^ centile) and weight 63kg (50^th^ centile). She had a broad mouth and short philtrum with minimal micrognathia. Her facial features were similar to her parents and siblings. Neurological examination revealed generally increased tone and brisk reflexes but no other specific features of note.

Investigations have included a normal fragile X, methylation for Angelman syndrome and *MECP2* testing. CT brain scan in childhood was reported as normal. Review in the clinic and retesting in the last 12 months confirmed a normal array, but *de novo* variant NM_001830.4(*CLCN4*):c.826C>G; p.(Leu279Val) which was reported by the laboratory as likely pathogenic.

**Family A17: NM_001830.4(*CLCN4*):c.840A>T; p.(Glu280Asp) *de novo***

This is an 8-year-old male born to non-consanguineous parents of Italian and Asian heritage. The pregnancy and delivery were uncomplicated.

In infancy there was global developmental delay and infantile hypotonia. He has a moderate intellectual disability diagnosed with psychometric testing at the age of 4. Autism spectrum disorder was diagnosed due to features of inattention, impulsiveness, restricted interests, and stereotypies. However, he is empathetic with good eye contact and is sociable. He occasionally has challenging behaviors triggered by a change in routine, for example moving from a classroom to the library, or when the environment is particularly noisy. He has excellent visuospatial skills and memory. He is strong, can climb and run independently. He can communicate using 4–5-word sentences. He has never had developmental regression. He attends a special class within a mainstream school with allied health support including occupational therapy and speech pathology and behavioral support.

He developed generalized tonic-clonic seizures at age 8 months, initially controlled with Levetiracetam, until the age of 6.5 years when he developed more frequent tonic-clonic and focal seizures, occurring every two weeks. Seizures were both associated with fevers and not. There was no response to the addition of Tegretol. Sodium valproate was introduced with good seizure control. He has now been seizure free for 7 months. His electroencephalogram previously showed multifocal epileptiform discharges, with prominence in the left parietal region.

He has mild constipation but no other significant gastrointestinal symptoms and eats well. Other clinical features include atopy with eczema, asthma, and allergic rhinitis.

Measurements at age 8 years were consistent with a weight of 23.6kg (tracking on 25^th^ centile), height 129cm (10^th^-25^th^ centile), head circumference 51.3cm (10^th^-25^th^ centile). He has a single cafe au lait macule, and his examination was otherwise normal. He tends to walk on his toes and jumps when he is walking. He walks with a slightly unusual, stooped posture.

Previously non-diagnostic investigations included chromosomal microarray, Fragile X PCR, urine metabolic screen, very long chain fatty acids, lysosomal enzymes, and CSF studies. MRI brain was normal.

Trio exome analysis identified a *de novo* variant NM_001830.4(*CLCN4*):c.840A>T; p.(Glu280Asp) classified by the laboratory as likely pathogenic.

**Family A18: NM_001830.4(*CLCN4*):c.848G>A, p.(Ser283Asn) *de novo***

This is a 37-year-old female proband of Caucasian (white British) heritage with moderate intellectual disability, autism, and obsessive behavior.

The birth was uncomplicated with delivery at term. Birth weight was 3.29kg (10^th^-50^th^ centile). In the neonatal period there were feeding difficulties including poor latch and hypotonia. She first sat at 11 months and walked at 2 years. Progressive spastic diplegia was diagnosed at age 2.

There was no regression, but autistic traits were noted from 3 to 4 years of age. Poor attention was also noted by a community Pediatrician in early childhood, but no formal diagnosis of ADHD was made. Speech as an adult is in full sentences. Schooling was in a special needs school, and she is now employed in a sheltered environment. She was referred to a psychiatrist aged 33 regarding explosive outbursts, obsessive shopping behaviors and overeating. She is treated with sertraline.

There have been issues with weight gain, and she has been trialed on metformin. Due to cushingoid features on examination it was recommended that she have investigations for Cushing syndrome, which were normal (not consistent with Cushing syndrome). She was recently diagnosed with obstructive sleep apnea, requiring CPAP. She also has gastroesophageal reflux, treated with omeprazole.

Examination findings include cushingoid features (round face and dorsocervical fat pad), crowded facial features, a small mouth, and relatively small hands. Most recent measurements were of height 168cm (75^th^ centile) and weight 110kg (>> 97^th^ centile): BMI is 39, in the obese range.

Chromosome microarray identified a 4q13.1q13.2 deletion, which was maternally inherited. Prader-Willi syndrome methylation and dosage studies were normal.

Further investigation through the 100,000 Genomes Project found a *de novo* variant NM_001830.4(*CLCN4*):c.848G>A; p.(Ser283Asn).

**Family A19: NM_001830.4(*CLCN4*):c.926A>G; p.(Asn309Ser) maternally inherited**

The proband is a 13-year-old male, the second born of two affected sons of non-consanguineous Caucasian parents.

There were no prenatal complications. Delivery was at 39 weeks: his birth weight was 4.59kg (>97^th^ centile), birth length 51cm (48^th^ centile). He had global developmental delay and infantile hypotonia. By way of motor milestones, he sat at 7-8 months and independent walking was achieved at age 23 months. He now speaks in sentences and has a broad base, ataxic gait.

He has a moderate to severe intellectual disability confirmed on psychometric testing and a diagnosis of autism spectrum disorder and anxiety before the age of 5 years. He has a short attention span but no formal diagnosis of ADHD. He attends an educational support school with speech therapy, occupational therapy, and physiotherapy. He is independent in dressing himself and can do simple food preparation.

On examination aged 13 his head circumference was 59cm (98^th^ centile), weight 52kg (75^th^ centile) and height 178 cm (90^th^ centile). On most recent examination he is noted to have a long face with square chin and upslanting palpebral fissures. He has long fingers, pes planus, and joint hyperextensibility. He has no gastrointestinal symptoms.

He has complete agenesis of the corpus callosum on MRI. Although not formally diagnosed with epilepsy he is currently being investigated by EEG for possible absence seizures.

His brother, aged 21, has intellectual disability, complete agenesis of the corpus callosum, ataxia and seizures. On psychometric testing he has moderate-severe intellectual disability. He completed schooling at an educational support school.

He has epilepsy: seizures commenced at age 13 years. Seizures were originally of focal onset (clonic in right upper limb) with secondary generalization. Initial EEG aged 13 years demonstrated epileptiform activity arising maximally from the left mid temporal region with secondary spread through the left hemisphere, with post ictal slowing in the left hemisphere. Seizures are currently reasonably well controlled on monotherapy with Tegretol, with a seizure frequency of less than one seizure per year.

He has obesity and early onset of puberty. He has recurrent abdominal pain, constipation, and ‘heartburn’.

On examination at age 21 his height was 179.2cm (>97th centile), weight 155kg (>>97^th^ centile) and head circumference 66cm (> 97^th^ centile). Clinical features include hypotonia, camptodactyly and bilateral strabismus. Like his brother he has a long face with square chin, long fingers, pes planus, and joint hyperextensibility. He also has a distinct philtrum and tented upper lip.

Their mother is aged 47. She completed school with remedial mathematics and English. She also has hyperextensibility and impaired coordination.

Exome sequencing identified a maternally inherited variant NM_001830.4(*CLCN4*):c.926A>G; p.(Asn309Ser) in both sons.

**Family A21: NM_001830.4(*CLCN4*):c.956T>C; p.(Phe319Ser) maternally inherited**

This is a 12-year-old male born to non-consanguineous Belgian/Caucasian parents with specific learning disabilities and pharmaco-resistant epilepsy.

Pregnancy and delivery were uneventful. His initial developmental milestones were within the normal ranges although slightly slower than his age matched peers. He had infantile hypotonia for which physiotherapy was initiated for a short period. He had an unstable gait and speech articulation difficulties. He does have a mild disharmonic intellectual disability with mainly a non-verbal learning disability based on spatial disability. His WISC-II-NL scores were: Verbal IQ 90; Performance IQ 61; VS 75. He attends mainstream school. He has hyperkinesis and a short attention span.

At age 9 months he developed generalized tonic-clonic seizures. These were often associated with febrile illnesses. From age 2-3 years there were absence seizures associated with eyelid myoclonus. The frequency of tonic-clonic seizures were a few times a year; absence events could occur multiple times per day. At last follow up, the tonic clonic seizures have resolved but absence seizures continue. Multiple EEGs have shown a normal background with frequent epileptiform activity consistent with generalized as well as focal (bilateral parietal) epileptic activity, (polyspike, spike-waves, but no typical 3Hz spike-waves). Of note, epileptic activity on EEG was much more prominent with eyes closed. He is therapy resistant. Anti-seizure drugs targeting generalized as well as focal seizures were tried, however without success (valproic acid, levetiracetam, lamotrigine, clobazam, zonisamide, perampanel, ethosuximide, topiramate, brivaracetam, felbamate, benzodiazepines (nitrazepam, ethylloflazepate), vigabatrin, oxcarbazepine, lacosamide). Ketogenic diet as well as corticotherapy did not improve seizure control. Considering the therapeutic potential of acetazolamide for myotonia congenita due to loss-of-function variants in *CLCN1,* this therapy was trialed too, but didn’t show clear reduction in seizure frequency. MRI brain scans were normal at age 2, 6.5 and 11 years. Lastly a vagal nerve stimulator was implanted, but it is too early to evaluate its impact on seizures.

The family history includes one older brother with autism spectrum disorder, a paternal grandfather with adult-onset epilepsy as well as maternal aunts with a history of febrile convulsions.

His last examination was at 12 years. His height was 152.8 cm (50^th^ centile), weight 24.8 kg (60^th^ centile), head circumference 55.4 cm (90^th^ centile). There was joint hypermobility noted, but no dysmorphic signs. Besides clumsiness and weak dynamic balance, the neurological examination was normal.

Other investigations include normal chromosomal microarray, MPS epilepsy panel, *SCN1A* sequencing. Exome sequencing on a research basis identified a maternally inherited *CLCN4* variant NM_001830.4(*CLCN4*):c.956T>C; p.(Phe319Ser). The mother is reportedly unaffected. Maternal X-chromosome skewing could not be tested due to same repeat region on both X-chromosomes. His unaffected brother did not carry the p.(Phe319Ser) variant.

**Family A22: NM_001830.4(*CLCN4*): c.1025G>A; p.(Gly342Glu) maternally inherited**

The proband is 3 years 1 month old, the first child of non-consanguineous Caucasian parents.

He was born at term+14 days after an uncomplicated pregnancy. Labor was induced but he was delivered by Emergency lower segment caesarian section for fetal bradycardia. He was born in good condition and weighed 3848g (82^nd^ centile). He fed well and there were no concerns in the first year of life. At a year of age, when he started nursery, it was noted he appeared not to be taking notice of his surroundings and he did not point or wave goodbye. He walked at 2 years and at 3, he is very active, uses 3 words inconsistently and uses gaze to indicate what he wants. He engages others in play by passing them objects and prefers cause and effect toys and boisterous play.

He had a single focal seizure causing rhythmic jerking of the right arm and leg which lasted approximately 2 minutes. EEG showed central spikes more prominent on the left and brain MRI was normal.

Chromosome microarray was non diagnostic. A next generation sequencing panel of 104 genes associated with early onset epilepsy identified a hemizygous missense variant NM_001830.4(*CLCN4*):c.1025G>A; p.(Gly342Glu). The same variant was detected at a low level on Sanger sequencing in his unaffected mother.

**Family A24: NM_001830.4(*CLCN4*): c.1078C>A; p.(Arg360Ser) maternally inherited**

The proband is a 18-year-old male born to non-consanguineous Iraqi parents. He was delivered at term with birth measurements weight 4078g (85^th^ centile), length 48cm (15^th^ centile), head circumference 33cm (15^th^ centile).

He presented with a decrease in social responsiveness and interaction from age 18 months. He did not speak until age 3 and was diagnosed with moderate developmental delay at age 5, at which time he was speaking in sentences and drawing. He had significant development regression at age 7 years and lost the ability to write his name, lost speech and purposeful hand functions gradually over months. He has not recovered these skills subsequently.

He was toilet-trained at age 3 but regressed to incontinence at age 7. FSIQ was 55 at age 5 prior to regression and the boy now has a severe ID with nonverbal status. He has some understanding of basic commands and attends special school. He was formally diagnosed with autism spectrum disorder at age 8. Development has plateaued since then.

He has a diagnosis of ADHD (managed with Clonidine) and sensory processing disorder. He was diagnosed with bipolar disorder at age 13 and can be extremely hyperactive (manic/hypomanic) for weeks, associated with poor concentration and no sleep for 48 hours, followed by weeks of low mood and lethargy. He was started on Tegretol and is now maintained on sodium valproate as well as olanzapine.

Other issues include pica and constipation with fecal loading. He has poor appetite and limited diet, requiring previously an iron infusion when 14 due to severe iron deficiency anemia.

He also has progressive thoracolumbar kyphosis and mild scoliosis with leg length discrepancy. He has significant genu recurvatum and anterior propulsion of the hip joints when walking. The kyphosis has worsened over the last 5 years since the onset of puberty. There is not a plan for surgical intervention and no vertebral segmentation anomalies on X-rays.

MRI brain showed non-specific bilateral small punctate frontal white matter hyperintensities, mildly prominent Virchow-Robin spaces. The corpus callosum appeared slightly bulky at age 11.5 years. Serial EEGs have been normal.

On examination at age 16 height was 175cm (50^th^-75^th^ centile), weight 43kg (<3^rd^ centile) and head circumference 54.5cm (10^th^-50^th^ centile). He had an elongated face, long nose, mildly anteverted ears, prominent chin, and a pre-auricular pit at base of left helix. His chin has become more squared with age. There was 5^th^ finger proximal interphalangeal joint camptodactyly, mild pectus excavatum, severe thoracolumbar kyphosis with leg length discrepancy, very thin habitus (lacking subcutaneous fat) with long arms and legs.

Trio exome sequencing identified a maternally inherited variant: NM_001830.4*(CLCN4*):c.1078C>A; p.(Arg360Ser). Mother’s X inactivation was consistent with random skewing of the X chromosome. She is asymptomatic with a normal cognition and no mental health diagnoses. Studies were done to determine whether this variant was present in other relatives. The proband’s intellectually normal brother (age 8), maternal grandmother and his maternal aunt do not have the variant. Given his maternal aunt does not have the gene variant, it is very unlikely that his late maternal grandfather had the variant, but germline mosaicism in the sperm cannot be ruled out. This indicates that the variant most likely occurred for the first time in his mother’s DNA at the time of conception. The family studies and pedigree also show that no intellectually normal male in the proband’s family is likely to have the same gene variant.

**Family A28: NM_001830.4(*CLCN4*):c.1465C>A; p.(Gln489Lys) *de novo***

The proband is a 32-year-old female, the first child to non-consanguineous Caucasian parents, with a history of specific learning disabilities, obsessive-compulsive disorder, depression, anxiety, gastrointestinal dysmotility and migraines. She also has joint laxity and joint pain, and symptoms of dysautonomia and skin fragility consistent with an additional clinical diagnosis of hypermobile Ehlers-Danlos syndrome.

She was not noted to be a flexible or hypotonic baby. The proband attended a mainstream school and has a full-scale IQ in the very superior range: WAIS-IV aged 21 was consistent with a full-scale IQ of 131. Academic testing revealed specific learning difficulties in reading, numerical operations, and mathematics reasoning. She has had difficulties with peer interaction but has not received a diagnosis on the autism spectrum. She was first diagnosed with mental health conditions in her early teens, treated with multiple medications including citalopram, diazepam, venlafaxine, haloperidol, and psychotherapy. She has required hospital admissions for her mental health concerns. She developed symptoms of tardive dyskinesia aged 28. She ceased mainstream schooling when 15 years and has had intermittent education and employment since then, complicated by multiple hospital admissions and operations.

Neurologic findings included migraines with posterior headaches, numbness and tingling of extremities, tremors, muscle twitches, and alterations in handwriting. She received a tentative diagnosis of epilepsy when she was 17 years old: with seizure semiology considered consistent with possible focal aware and focal impaired awareness events. On sleep deprived EEG she had rare poorly formed generalized sharp waves. She was trialed on multiple antiepileptics including topiramate and vagal nerve stimulator. However, the diagnosis of epilepsy was later doubted due to absence of clear epileptiform activity on EEG, and her antiepileptic was able to be weaned without recrudescence of events.

She was aware that she was ‘double-jointed’ as a child, and her ability to participate in yoga, dancing, art and music were curtailed by pain, injury, and/or fatigue. By age 11 she was having joint pain with need for braces, pain medications, and muscle relaxants. As an adult her joints ‘pop’ with movement and she has had overall joint and body pain that prompted the diagnosis of fibromyalgia. Her joints often sublux such that she needs help to replace them and has mild scoliosis and spinal disc problems along with temporomandibular joint pain. By age 25 she required a walker to help with mobility. She has a clinical diagnosis of hypermobile Ehlers-Danlos syndrome.

She had difficulties establishing breastfeeding and was switched to soy formula. She has significant gastrointestinal dysmotility with symptoms of severe gastroesophageal reflux and constipation and confirmed delayed gastric emptying and small bowel transit time on nuclear medicine studies. She has required Nissen fundoplications, gastrostomy and jejunostomy.

Due to chronic abdominal pain and headaches, she is on numerous analgesics and gabapentin. Other conditions include severe asthma, persistent rhinosinusitis, chronic sinusitis and otitis, endometriosis, hypothyroidism. She has needed glasses since the age of 7 and orthodontics with extractions for dental crowding by age 14. Surgeries have included an attempted ablation for SVT treatment with inability to pinpoint an arrhythmogenic focus, two esophageal dilatations, sinus surgery, thyroidectomy, and subtotal hysterectomy with bilateral salpingo-oophorectomy.

MRI brain, conducted due to raised prolactin levels, showed a possible pituitary microadenoma, pituitary hyperplasia or Rathke cleft cyst – appearances were stable over time, and otherwise neuroimaging has been normal. Other testing showed thyroiditis with consequent hypothyroidism, low vitamin B12 levels responding to supplementation, and an episode of adrenal insufficiency.

On examination aged 22 her weight was 68kg (75^th^-90^th^ centile) and height 160cm (25^th^-50^th^ centile). She is not noted to have any dysmorphic features. She has a long face, soft, lucent skin with mild elasticity and no unusual scars, normal fingers with sufficient hypermobility to perform the Walker-Murdoch and Steinberg maneuvers, lordosis but minimal scoliosis on exam, and hypermobility with a Beighton score of 7/9. There are no focal features on neurological examination, but she did have poor balance compatible with her need to use a walker.

Exome sequencing done to investigate her connective tissue symptoms detected the *CLCN4* variant NM_001830.4(*CLCN4*):c.1465C>A; p.(Gln489Lys). Segregation in both parents revealed this was *de novo*. Mitochondrial testing revealed a novel homoplastic MT-RNR2 m.3110C>T variant in a non-conserved 16S rRNA loop region that may affect its secondary structure/function. The MT-RNR2 variant was also homoplastic in her mother who has shared symptoms of hypermobile Ehlers Danlos syndrome.

**Family A29: NM_001830.4(*CLCN4*):** **c.1576G>A; p.(Gly526Ser) maternally inherited**

The proband is a 17-year-old Portuguese male with mild intellectual disability and dyscalculia. He has a short attention span and impulsiveness. He had febrile seizures starting at the age of 1 year, which progressed to multiple seizure types including focal onset seizures with secondary generalized seizures and myoclonic seizures. The seizures are treated with valproic acid. He also has neutropenia and anemia.

He has a brother and maternal uncle with learning difficulties.

He has severe feeding difficulties and has a head circumference in the 2^nd^ centile and weight and heigh less than the 5^th^ centile. He has hypoplastic upper maxilla and dental malocclusion.

He has a normal karyotype, MLPA for subtelomeric regions, and screening of *FMR1, AFF2, ARS* and *MED12*. Exome sequencing identified a maternally inherited NM_001830.4(*CLCN4*):c.1576G>A; (p.Gly526Ser) variant.

**Family A31: NM_001830.4(*CLCN4*): c.1597G>A; p.(Val533Met) maternally inherited**

The proband is a 15-year-old Portuguese male with moderate intellectual disability (IQ 45), short attention span and difficulties with socialization. He has had focal onset seizures from the age of 5 years, treated with valproic acid and topiramate.

His weight and height are in the normal range.

He has had normal screening of *FMR1* and *SCN1A*. Exome sequencing identified a NM_001830.4(*CLCN4*):c.1597G>A; (p.Val533Met) variant inherited from his mother.

**Family A40:** **NM_001830.4(*CLCN4*):c.1904C>G; p.(Pro635Arg) maternally inherited**

This is a Caucasian family with 2 affected female heterozygotes, a female child, and her mother.

The proband is an 8 year, 7-month-old girl with moderate intellectual disability. Her mother has mild intellectual disability, anxiety and depression.

The child has moderate intellectual disability as well as autistic traits, ADHD, hyperkinesis and short attention span. She has some challenging behaviors, sleep disturbance and anxiety.

She has epilepsy with first seizures at the age of 2 years: focal and generalized tonic clonic seizures. She is currently managed on monotherapy with lamotrigine (8mg/kg/day). Previously sodium valproate was used. EEG was normal at presentation. Brain MRI was abnormal with underdevelopment of the sulci in the left frontal region suggestive of an isolated congenital anomaly. Subsequent MRI at 4 years showed persistent widening of the CSF space overlying the left frontal region.

Examination at age 7 years 6 months showed a head circumference of 54.5cm (50^th^-75^th^ centile), weight 29.2kg (90^th^ centile) and height 127cm (50-75^th^ centile). She has hypertelorism with epicanthal folds and full cheeks.

Investigation through the 100,000 Genome project identified a maternally inherited variant NM_001830.4(*CLCN4*):c.1904C>G; p.(Pro635Arg).

No other likely pathogenic variants have been identified. With regards to family history there is a maternal uncle with severe intellectual disability: so far this individual has not been able to be tested. The variant is not present in the maternal grandmother.

**Family A41: NM_001830.4(*CLCN4*):c. 1906G>A; p.(Val636Met) *Maternally inherited***

The proband a 4-year-old girl, is one of three children born to a French couple. She has two sisters, 5 and 1 years old. She was born at term (37 weeks gestation) with a history of intrauterine growth retardation. Her birth weight was 2.15 kg (3^rd^ centile), height 42 cm (3r^d^ centile), and head circumference: 31.5 cm (3^rd^-10^th^ centile).

She presented with both motor and language delays. She sat at 9-10 months, stood at 1 year, walked at 23 months. She only has a few words at four years. She is looked after at home by her father. She goes to school for one hour in the morning and for one hour in the afternoon. She receives speech therapy, physiotherapy therapy, and psychiatric therapy. She is waiting for specialized education. There has been no regression in her skills.

She was referred to us during her mother's new pregnancy. She has a moderate global development with autistic features (hyperactivity, hyperkinesia, short attention span). There is no sleep disorder. On examination, she has skin lesions of scratching and biting. There is no history of seizures, ataxia or hypotonia. She has mild feeding difficulties and selects mixed and smooth foods.

At age 3 years and 8 months her height was 0.86 m (- 2SD), weight 12.4 kg (-2 SD), head circumference -3 SD). On examination she has down slanting palpebral fissures, telecanthus, small and widely spaced teeth. She has bilateral clinodactyly of the 5th fingers and slight oedema of the dorsum of the feet.

Her two sisters do not have developmental delay or autistic features. Her mother has no history of learning difficulties at school and has a postgraduate language diploma 2 years. Neither of her parents have developmental or learning difficulties. There are no learning difficulties in the paternal family.

Singleton exome sequencing was done in the proband which found the following variant NM_001830.4(*CLCN4*):c.1906G>A; p.(Val636Met). On segregation this was found to be maternally inherited. Her two sisters and maternal grandparents have not been tested. She has a paternally inherited chromosomal deletion: deletion of exons 16 to 23 in *TNRC6B*: arr[GRCh37] 22q13.1(40731210_40864621)x1. Other normal investigations include screening of *FMR1* and methylation studies for Angelman region.

**Family A42: NM_001830.4(CLCN4):c.2152C>T; p.(Arg718Trp) *de novo***

This is a 6-year-old female with global developmental delay, now consistent with intellectual disability, autism spectrum disorder, localization related epilepsy well controlled on Oxcarbazepine and sleep disorder. She was conceived naturally to a 33-year-old primigravid mother and 37-year-old father. The pregnancy was uncomplicated. There were no maternal fevers, infections, spotting, bleeding, antibiotic use, trauma during the pregnancy. Her mother was on no medications during pregnancy and there was no prenatal exposure to alcohol, tobacco, illicit drugs. There was regular prenatal care. Prenatal ultrasound imaging was normal.

The proband was born full term via normal spontaneous vaginal delivery with unremarkable labor. The birth weight was 3.94 kg (50^th^-75^th^ centile). She was in the newborn nursery and discharged home with her mother at the expected age. There were no medical issues or concerns in the newborn period. She was described as an “easy” baby with typical first year of life, though difficulty with sleep was reported. At 12 months of age her parents were concerned that she was not yet babbling. By 15 months of age, she qualified for physiotherapy due to concerns with her gross motor milestones, and then by 18 months of age for speech and language therapy and occupational therapy due to global developmental concerns. She walked at 18 months of age. At age 6 she makes sounds and has echolalia but is very limited in her spontaneous expressive speech. She attends specialist education with speech and language therapy 5 times a week, physiotherapy twice a week and occupational therapy three times a week. She has 1 on 1 support. There is current concern with regression of skills. She can identify letters of the alphabet and point to and identify pictures.

She has a formal diagnosis of autism spectrum disorder. There are the following difficulties identified on last review aged 6. She has deficits in abnormal approach, back and forth conversation, sharing of interests or emotions and failure to initiate or respond to social interactions She has difficulties with poorly integrated verbal and nonverbal communication, poor eye contact and body language. She has difficulties in sharing imaginative play and absence of interest in peers. She has difficulties with stereotyped or repetitive motor movements, use of objects, and speech: with the presence of echolalia. She has extreme distress at small changes and difficulties with transitions. She has a strong attachment to or preoccupation with unusual objects. She has excessive smelling or touching of objects and visual fascination with lights or movement. There are also concerns for inattention, hyperactivity, and impulsivity. She was recently started on clonidine which has helped with her attention, but she is still quite active. It has helped slightly with her sleeping, but this still significantly impaired at this time. She is not aggressive, but she does not recognize dangerous situations and there is concern for elopement.

She does have anxiety. She is still not full toilet trained. She can feed herself with utensils. She is dependent on others. She has extremely poor sleep treated with melatonin and clonidine. She has difficulty falling asleep and wakes up most nights as well. She has been waking up in the middle of the night giggling. She has multiple stereotypies like pacing, spinning, bouncing.

She has a diagnosis of epilepsy with seizures first recognized at the age of 2 years. The seizure semiology is of blank stares, cyanosis, and emesis followed by sleep for about 20-30 minutes. The events often seemed to have been triggered by motion. EEG have shown mild generalized slowing with no epileptiform activity. She is treated with oxcarbazepine which has had a good effect.

She is a picky eater. Her hearing and vision have been normal by parent report.

On recent examination aged 6 years her height was 1.25m (90^th^ centile), weight 31.8kg (>97^th^ centile), and head circumference 50.5cm (50^th^ centile). She was noted to have inconsistent eye contact, her speech was mainly sounds and echolalia. Her cranial nerve examination was normal. She has normal tone and strength, sensation intact to light touch. She was noted to be constantly in movement. She can walk alone without assistance and run and hop but has difficulties with heel, toe, and tandem walking.

Targeted MPS gene panel identified a *de novo* variant NM_001830.4(*CLCN4*):c.2152C>T; p.(Arg718Trp).

**Family A51: NM_001830.3 (*CLCN4*): c.2192G>T; p.Gly731Val maternally inherited**

This is a 11-year-old boy born to Belgian parents. His mother is clinically unaffected, and the parents have an unaffected 8-year-old daughter. His father went to a special needs school because of learning difficulties.

The pregnancy was uncomplicated and the proband was born at 41 weeks of gestation. There were no neonatal concerns. Neonatal adaptation was normal, other than being noted to have a large head circumference. His birth weight was 3.5 kg (50^th^ centile) and length 52cm (85^th^ centile). He walked at 13 months and there were no speech delays. At 4 years of age, when attending school, he was diagnosed with fine motor difficulties and limited attention. His verbal IQ aged 5 years was 97, performance IQ 87 and his processing speed was 100. In primary school he attended a class for children with learning difficulties and required special educational provisions, such as the use of a tablet. He was treated with methylphenidate which improved his attention. His expressive language is mildly delayed. He has diagnoses of dyslexia, dyspraxia, and dyscalculia. His gross motor development is age appropriate. At the age of 10 years old, he can count to 30 and has syllabic reading. He has temper tantrums and prefers routines. He has had 2 years of physiotherapy and occupational therapy and now attends a special needs school and has speech and physiotherapy. He has had no developmental regression.

He is generally healthy. He is described as a picky eater.

He has never had an epileptic seizure: a 24 hour EEG did not show epileptic activity but demonstrated an excess of slow waves. Brain MRI at 18 months was normal.

Aged 10 years old his head circumference was 58.5 cm (98^th^ centile), height 154.2 cm (98^th^ centile) and weight 50.5 kg (98^th^ centile). He has mild dysmorphic features including synophrys, straight eyebrows, a high nasal bridge and enophthalmia.

Chromosomal microarray was normal. Trio clinical exome analysis identified a maternally inherited missense variant NM_001830.4(*CLCN4*):c.2192C>T; p.Gly731Val. His mother has normal X-inactivation (39/61) and is unaffected.

## Group B: (Likely) pathogenic: rare missense variants with functional studies in *Xenopus* oocytes consistent with a gain-of-function, and no clear-cut alternative genetic diagnosis.

**Family B1: NM_001830.3 (*CLCN4*): c.265G>A; p.(Asp89Asn) *de novo***

This is a 13-year-old girl. She has non-affected non-consanguineous Caucasian parents. The pregnancy and delivery were uneventful.

She was referred for a clinical genetics consultation due to concerns with microcephaly, with delayed psychomotor development, particularly affecting her speech. She sat at 9 months of age and walked at 15 months. She had marked speech delay: only 5 words at 3 years of age. At ten years she could speak simple sentences but with pronunciation difficulties. She attends a specialist school, with speech and language and physiotherapy input. She is treated with risperidone for anxiety and has attention deficit disorder

She achieved day and night continence at 6 years of age. She has ataxia and frequent falls. It has been queried whether she has absence seizures, but EEG was normal.

She has some feeding difficulties: finding it difficult to chew solid food. She has gastro-esophageal reflux. She has relative microcephaly, and subtle dysmorphic features including a broad nasal root, and high palate, fifth finger clinodactyly. She has a pectus carinatum.

She has a normal chromosomal microarray and karyotype. Exome sequencing identified a de novo variant NM_001830.3 (*CLCN4*):c.265G>A; p.(Asp89Asn).

**Family B3: NM_001830.4(*CLCN4*):c.804T>G p.(Phe268Leu) *de novo***

This is an 8-year-old American female, with a diagnosis on the autism spectrum, mild intellectual disability, and dysgenesis of the corpus callosum.

She was delivered at term via planned caesarian section with a birth weight of 8 pounds 5 ounces. She did not require any special medical care at birth. At 6 months of age, it was noted that her head circumference was small for her age and in view of this and delayed developmental milestones she was referred for developmental assessment. She was noted to have delayed fine motor skills, low tone and was less engaged in play. A provision diagnosis of sensory processing disorder was made and speech and language therapy, occupational therapy and physical therapy commenced. She continued to make good progress with additional support, and her first independent steps were at 2 years of age. She required ankle foot orthoses until she was 3 ½ years old.

She had significant feeding difficulties as an infant, with difficulties in chewing and swallowing and was diagnosed with severe gastroesophageal reflux. She had suboptimal weight gain and was prescribed Neocate formula, which she continued for two years. She transitioned to pureed food at 9 months and then more solid food at 18 months but had ongoing difficulties with eating.

At the age of 8-9 months, a frenulotomy was completed for tongue tie. On audiology assessment (Auditory Brainstem Response) she was found to have mild hearing impairment and has eustachian tubes inserted at 12 months of age. She continues to have recurrent ear infections, although heading and vision are reported to be within normal limits. She has disordered sleep and finds it difficult to self soothe if she wakes from sleep. She is prescribed melatonin to help with sleep initiation and Trazodone to help with sleep maintenance.

Due to concerns with her development, she attended a preschool special education classroom and had a comprehensive developmental assessment aged 5 years, at which point she was diagnosed as being on the autism spectrum. She received special education, speech and language therapy, occupational therapy, and physical therapy. She currently attends a special education (autism support) program at a mainstream school and receives both life skills and autistic support as well as allied health support: occupational, physio and speech and language therapies. A full-scale Wechsler Intelligence Scale for Children, Fifth Edition (WISC-V) was unable to be completed fully but her composite score was 55. Testing on specific subtests on the Wechsler Individual Achievement Test-Third Edition (WIAT-III) demonstrated challenges with listening comprehension early reading and math, with relative skills in receptive vocabulary and expressive communication.

Despite her diagnosis on the autism spectrum, she is highly sociable, and enjoys imaginative play. She has strengths in copying others and responds well to targeted therapies and direct feedback. She can be easily over stimulated. She seeks sensory input and is soothed by pressure therapies, riding in a car, and listening to music. She prefers routine, and likes lining up objects, placing objects in specific positions and has preferences for certain patterns.

MRI brain demonstrated a lipoma and dysgenesis of the corpus callosum. Due to concerns regarding possible seizures, she had sleep studies and EEG- she did not have a confirmed diagnosis of epilepsy. Previous normal investigations include metabolic screening including CSF studies and screening for congenital disorders of glycosylation. Chromosomal microarray was normal. Exome sequencing detected a *de novo* rare missense variant in *CLCN4,* NM_001830.4(*CLCN4*):c.804T>G; p.(Phe268Leu).

**Family B4:** **NM_001830.4(*CLCN4*):c.928C>T p.(Pro310Ser) *de novo***

The proband is a 7-year-old Caucasian (American) who has unaffected parents and full sister. As an infant she had hypotonia and global developmental delay. Since infancy her development has continued to be delayed but steady. She walked at age two years at which time she said her first words. She now has limited speech (about 20 words) and has just been toilet trained. Seizures first occurred at age 4 year and initially were treated with levitriacetam after which she developed extreme aggression and behavior issues. She then was switched to zonisamide with improvement in her behavior. However, she still pulls hair, and hits and bites others. She does not read or write but does have some self-help skills include feeding and dressing. She also does interact with other children, attends dance classes, and plays with her sibling.

On exam at age 7 years, her height was 112.0cm (3^rd^ centile), weight 22.3kg (4^th^ centile) and occipitofrontal circumference of 48.0 cm (-2.1 SD). Other findings included mild microcephaly with ridged metopic suture, strabismus and astigmatism that have worsened with age, left-sided ptosis, slight ocular telecanthus, lateral fullness of the nose, mild bilateral limited elbow extension, a transverse palmer crease, mild hypotonia, hand tremor and good strength.

An EEG at the age of 4 years showed slow waves. A head MRI at age 5 years was normal as was a chromosomal microarray analysis. Her *CLCN4* variant was found on an autism/intellectual disability gene sequencing panel, a *de novo* variant NM_001830.4(*CLCN4*):c928C>T; p.(Pro310Ser).

**Family B5: NM_001830.4(*CLCN4*):c.949G>A; p.(Val317Ile) *de novo***

The proband is a 5.5-year-old French male, with severe global developmental delay, hypotonia, broad based gait and partial agenesis of the corpus callosum.

Delivery was at 38 weeks with birth weight 4.300kg (>>90^th^ centile), length 49cm (50^th^ - 90^th^ centile) and head circumference 35cm (50^th^ - 90^th^ centile). He had infantile hypotonia.

He had delays in all areas of development: walking at 5 years and remaining nonverbal at 5 ½ years of age. He walks with a wide based gait. There are concerns with his vision in low light conditions. He has a formal diagnosis of autism spectrum disorder and can be very anxious if there are strangers in a room. He has had no seizures.

He has established oral feeding, although there are feeding difficulties as well as constipation and gastro-esophageal reflux. He also has scalp psoriasis and polyarthralgia.

At 3.5 years his height is 91cm (2^nd^ centile), weight 13kg (3^rd^ - 10^th^ centile), and head circumference 48cm (2^nd^ - 50^th^ centile). He has mild dysmorphic features with a mildly flat face, everted lower lip, anteverted nares.

Brain MRI at age 1 years showed a partial corpus callosum agenesis (posterior part of the corpus and splenium) with colpocephaly, and mild third ventricle dilation.

He has a normal chromosomal microarray and *FMR1* repeats are in the normal range. Exome sequencing identified a *de novo* variant NM_001830.4(*CLCN4*):c.949G>A; p.(Val317Ile).

**Family** **B6: NM_001830.4(*CLCN4*):c.949G>A;** **p.(Val317Ile)** **maternally inherited** **(mosaic in mother)**

This is a 13-year-old male, born to non-consanguineous parents of Mexican and Guatemalan/ Mexican parents. He has three unaffected sisters and one unaffected brother. His phenotypic features include moderate intellectual disability, optic hypoplasia, and dysplasia of the corpus callosum.

The pregnancy was complicated by diet controlled gestational diabetes. Delivery occurred at 38 weeks with a birth weight 2.75kg (10^th^ - 50^th^ centile) and length 45.7cm (10^th^ centile).

He had global developmental delay: walking did not occur until 3.5 years and speech was delayed until age 2. At 12 months of age, he was noted to have head lag and mild hypotonia with ventral suspension. There is ongoing speech therapy and assistance is needed with toileting. There has been a formal diagnosis of moderate intellectual disability at early school age. Schooling is in a special education class. In 8th grade his performance was consistent with 1st-2nd grade ability. He has behavioral and attention issues (diagnosed around age 9-10 years) requiring use of guanfacine medication and applied behavioral analysis therapy.

Past medical history is of recurrent otitis media with ventilation tubes placed at age 20 months He has bilateral optic nerve hypoplasia, myopia, and astigmatism. He had strabismus surgery at 3 years and 9 years. He has severe constipation with encopresis; a period of unexplained weight loss resolved, and his BMI is ~50% currently.

At 13 years 7 months his height is 152.9 cm (25^th^ - 50^th^ centile), weight 43.1 kg (25^th^ - 50^th^ centile) and head circumference 56cm (50^th^ - 98^th^ centile). He has elevated finger pads and fifth finger clinodactyly. There was a right preauricular skin tag.

MRIs around the age of 1 year demonstrated small hyperintense focal areas in the left prefrontal cortex, and then partial agenesis of corpus callosum with atrophic rostrum and blunting of the genu. MRI aged 9 showed a mildly small optic chiasm and optic nerves bilaterally suggestive of optic hypoplasia as well as a dysplastic corpus callosum. MRI at age 13 years showed resolution of the left prefrontal cortex, abnormal signal, and stable dysplasia of the corpus callosum. The hypoplastic optic nerves and chiasm were less well depicted. EEG age 9 years revealed slow background rhythms for age. A repeat EEG age 13 years showed no electrographic or electroclinical seizures. Video EEG to investigate spells showed findings consistent with focal onset frontal lobe hypermotoric epilepsy. Treatment with Oxcarbazepine was started with improvement.

Exome sequencing identified a maternally inherited variant in *CLCN4* (NM_001830.4):c.949G>A; p.(Val317Ile). He also has a normal chromosomal microarray and a variant of uncertain significance on mitochondrial DNA (m.6347 C>A; p.(Phe148Leu)).

The proband’s mother is 42 years old and has gastro-esophageal reflux disease and left sided aural atresia with absent hearing on that side. She has myopia and astigmatism. She was delivered at term; her birth measurements are unknown. Schooling was via mainstream education. However, her school performance was below average, and schooling was ceased in the 10th grade. As an adult she has worked in factory settings until the birth of the proband 13 years ago. Her height is 156cm (10-25th centile), weight 77 kg (90-97th centile), head circumference 56cm (50th-98th centile). Segregation identified that she carries the same variant as her son, but in mosaic form NM_001830.4(*CLCN4*):c.949G>A; p.(Val317Ile).

**Family B7: NM_001830.4(*CLCN4*):c.949G>A; p.(Val317Ile) *de novo***

The proband is an 18-year-old male with severe intellectual disability. He has a history of infantile hypotonia and global developmental delay, with concerns first raised with his development at the age of 8-9 months. He is the first child of non-consanguineous parents, and there was no significant family history. He sat at 18 months, walked at age 3 years, and had his first words at age 4 years. He has not had any developmental regression. He has autistic traits, appears to have a high pain threshold (he never cries), and his behavior can be challenging.

He has bilateral optic atrophy and myopia, and there are no concerns with his hearing. He had one seizure in the context of a febrile illness but has not had any seizures subsequently. He is described as a ‘picky’ eater, has had suboptimal weight gain, and has a gastrostomy. He has disordered sleep, which has not improved with melatonin.

At age 4 years, his head circumference was on the 10^th^ centile, weight between the 9^th^ and 25^th^ centile and height on the 2^nd^ centile. As a young child he was noted to have a myopathic face, with bilateral ptosis, widely spaced teeth, and slightly simple ears. As an adult, he has malar flatness with a long face and pointed chin.

His genetic investigations include a normal chromosomal microarray, normal Angelman syndrome methylation studies, and a negative screen for Fragile X syndrome and myotonic dystrophy type 1. His brain MRI scan at age 3 noted prominent subarachnoid spaces, and his MRI scan at age 8 noted hypoplasia of the corpus callosum. Whole genome sequencing identified a *de novo* variant NM_001830.4(*CLCN4*):c.949G>A; p.(Val317Ile).

**Family B9: NM_001830.4(*CLCN4*):c.1185C>G; p.(Ser395Arg) *de novo***

This is a 13-year-old female whose non-consanguineous Caucasian parents were 35 at her conception.

There were no prenatal complications, and she was delivered at 40 weeks gestation. The APGAR Scores were 10 at 1 and 5 minutes. Birth length was 50.5cm (50^th^ -90^th^ centile), weight 3300g (10 - 50^th^ centile) and head circumference 33.5cm (50^th^ - 90^th^ centile). In the immediate postnatal period, there was hypocalcemia.

In infancy there was gross motor delay with sitting at 14 months and walking at 24 months. Her first words were spoken at 30 months. She can walk independently and speak in sentences. There has not been any regression of skills. She has a diagnosis of a mild intellectual disability. Schooling has been in a special unit since the age of 4 years. She can dress herself, feed herself and is independent with her toileting. She has no history of epilepsy. She is hyperkinetic, and has a short attention span, impulsiveness, obsessiveness, rigidity, anger outbursts and anxiety. She receives psychotherapy from a psychologist and occupational therapy.

She is orally fed and has no gastrointestinal symptoms.

At age 11 her height was 149.5cm (75^th^ centile), weight 39kg (50^th^ - 75^th^ centile), head circumference 50cm (2^nd^ centile).

Investigations include a normal brain MRI at age 4 years. On repeat at age 6 an arachnoid cyst was identified. Karyotype and chromosome microarray were normal. Exome sequencing identified a *de novo* *CLCN4* variant NM_001830.4(*CLCN4*):c.1185C>G; p.(Ser395Arg).

**Family B10:** **NM_001830.4(*CLCN4*):c.1646T>A p.(Ile549Asn) *de novo***

This is a 7-year-old girl born at term after an uneventful pregnancy with low birth weight and microcephaly. She had early onset faltering growth. Regression was first noticed at 4-6 months, when she stopped social eye contact and had reduced social interaction. She developed stereotypic midline hand movements and her purposeful hand function was impaired. She also had regression of motor and speech development.

She made subsequent progress in her motor and speech development from the age of 11-12 months, however she remained severely delayed. Her first words were at 24 months. She was able to walk independently at 5 years. She currently attends a specialist school. Her speech is limited to single words. She can use nonverbal communication. She needs help for all activities of daily living, including eating and dressing.

She has never had seizures. She had feeding difficulties from the start and only tolerates pureed or soft food and currently needs hypercaloric food supplement.

On examination at 14 months, she was noted to have microcephaly, short palpebral fissures, and a prominent lower lip. At her most recent examination, aged 2 years, her length was 81.5cm (7^th^ centile; Z score -1.49) weight 6.6 kg (<<3^rd^ centile; Z score -7.9) and height head circumference 40.5 cm (<<3rd centile; Z score -8.0).

Other negative investigations included chromosome microarray and sequencing of *MECP2 and FOXG1*. Brain MRI at 8 months showed delayed myelination and considerable white matter deficiency with enlarged lateral ventricles and a thin corpus callosum.

Exome sequencing identified a *de novo* variant NM_001830.4(*CLCN4*):c.1646T>A; p.(Ile549Asn).

**Family B11: NM_001830.4(*CLCN4*) c.1648G>C; p.(Val550Leu) *de novo***

The proband is a 20-year-old woman, born to a non-consanguineous Belgian couple, at 41+3 weeks gestation. She was delivered after an uncomplicated pregnancy with a birth weight of 3.2 kg (10^th^ - 25^th^ centile) and a birth length of 47.5 cm (<3^rd^ percentile). There were no postnatal complications.

She had delayed motor milestones (rolling over at 8 months, sitting at 12 months, walking at 18 months). Her initial language development was not delayed. She was diagnosed with a mixed receptive-expressive language disorder, delays in visual, gross, and fine motor skills, and a mild intellectual disability at the age of 5 years 2 months: IQ score of 57 (SON-R 2½-7, harmonic profile). Her skills can fluctuate but she has had no consistent regression of skills. She has not met formal requirements for a diagnosis of autism spectrum disorder (ASD) but was noted to have high anxiety and some features on the autistic spectrum. At the age of 6 years and 10 months psychometric testing (WPPSI-R) was consistent with a total IQ of 66 (verbal IQ 61 and performance IQ 77).

Currently she has basic conversational communication skills. More recent psychometric testing has been consistent with a formal diagnosis of moderate intellectual disability (ID) with a total IQ of 49; her verbal comprehension and processing speed indices are consistent with a moderate intellectual disability level; working memory and perceptual organization indices are consistent with a mild intellectual disability level. She can read and write. She is employed in a supportive workplace setting. She is highly anxious in new situations. She has bowel and urinary continence in times of high stress.

She has not had any seizures. An EEG at the age of 4 years and 8 months was normal. She has had recurrent urinary tract infections and pyelonephritis, and vesicoureteral reflux, several allergies and dry skin with recurrent eczema.

On examination, her height was 160.3 cm (10^th^ centile), weight 84.5 kg (99^th^ centile), and head circumference 56 cm (70^th^ centile). She was noted to have minor dysmorphic features including a round face, deep-set eyes, bulbous nose with small nares, partial cutaneous syndactyly of the 2nd and 3rd toes. She has joint hypermobility.

She has two younger brothers (17 and 11 years of age). One has attention deficit disorder which is treated with medication. Neither of her parents have developmental or learning difficulties, both having obtained university degrees.

A customized panel of neurodevelopmental genes off a trio clinical exome sequencing backbone detected following variant NM_001830.4:(*CLCN4*):c.1648G>C; p.(Val550Leu) *de novo*. Other investigations were normal: (chromosomal microarray and *FMR1* repeat analysis).

**Family B12: (previously reported but now with updated clinical information NM_001830.4(*CLCN4*):c.1664C>T; p.(Ala555Val) *de novo***

The proband is a now a 9-year-old girl. This patient was previously included in the Palmer *et al*., (2018) publication (1), but due to this variant being recurrent in three other females, the clinician was recontacted and provided this updated information.

The proband is of mixed African American and Mexican heritage. She as delivered at 40 weeks and 3 days gestation by emergency caesarian section, due to concerns during labor with fetal distress and bradycardia. Her birth weight was 9lb 4 oz (4.2kg; +2 SD). There were no immediate postnatal complications.

She had global developmental delay: sitting at 9 months. She is non ambulatory and mobilizes via a wheelchair. She can scoot around on her bottom and turn from side to side when sitting. She is non-verbal There has been no regression of skills. She follows special education schooling and accesses physiotherapy twice a week, and occupational and speech therapy once a week. She has no diagnosis of autism, or ADHD.

There have been ongoing concerns with her feeding and weight gain, and she is currently fed via a gastrostomy tube. She has significant constipation. Her most recent growth measurements aged 9 years 1 month are: weight 26kg (25-50^th^ centile); head circumference 48cm (<2^nd^ centile); height 127.6 cm (3^rd^-10^th^ centile).

Brain MRI demonstrated diffuse cortical volume loss with mild lateral and third ventricular enlargement. She had a normal EEG aged 4 months of age.

The *de novo* NM_001830.4(*CLCN4*):c.1664C>T; p.(Ala555Val) variant was detected on trio whole exome sequencing.

**Family B13: NM_001830.4(*CLCN4*):c.1664C>T; p.(Ala555Val) *de novo***

This a 3-year-old female proband of mixed-African American and Mexican ethnicity with moderate global developmental delay and a marked short stature, low weight, and microcephaly. She also has spastic cerebral palsy and ichthyosis.

She was delivered at 40 weeks by emergency caesarian section due to fetal distress and bradycardia during labor. Her birth weight was 2.74kg (<10^th^ centile).

She is symmetrically small: at 3.5 years her height was 83.4 cm (<<1^st^ centile) and at 4 years 3 months 89 cm (0.1^st^ centile, Z= -3.08). Her weight at 3.5 years was 9.74kg (<0.1^st^ centile; Z score -5.50) and at 4 years 3 months 10.1 kg (<0.1^st^ centile, Z= -4.72). She also has severe microcephaly: at 3.5years 39.5 cm (<2^nd^ centile) and at 4 years 3 months 39.5 cm (<0.1^st^ centile, Z= -6.43).

She had milk protein intolerance, allergic colitis, oropharyngeal dysphagia, constipation, and gastro-esophageal reflux disease. She is currently fed via gastrostomy tube.

At the age of 4 years, she can sit independently, but is not yet walking or crawling; she scoots with purpose and rolls to where she wants to go. She can coo but has no specific words. There is no regression noted. Allied health intervention includes physiotherapy, occupational therapy, speech therapy, aquatic therapy, and nutrition support. There has been no formal developmental evaluation.

On examination findings she has positional plagiocephaly, deep-set and wide-spaced eyes, a broad bulbous nose, large ears, a small jaw, and high palate.

Cerebral MRI showed agenesis of the corpus callosum and anterior commissure (complete commissural agenesis) and abnormal orientation of the hippocampi. No other migrational anomaly was seen. EEG around age 1year, showed atypical background features including slowing.

Trio MPS panel testing (GeneDx) identified a *de novo* *CLCN4* variant: NM_001830.4(*CLCN4*):c.1664C>T; p.(Ala555Val).

**Family B14: NM_001830.4(*CLCN4*):c.1664C>T; p.(Ala555Val) *de novo***

This 4-year 5-month-old female patient is the second child of healthy non-consanguineous Caucasian/Singapore-Chinese parents.

She was detected antenatally to have microcephaly at the 20-week anomaly scan. Pregnancy was otherwise uncomplicated. She was born at 39 weeks gestation at which time her head circumference was recorded as being on the first centile. Brain MRI at 10 days of age revealed microcephaly, small pons, and immature myelination.

She had early feeding difficulty and severe gastroesophageal reflux requiring multiple anti-reflux medications (omeprazole, Gaviscon and azithromycin). By 4-6 months of age there were increasing concerns about emerging global developmental delay and inconsistent visual behavior. By 6 months of age her head circumference was 37cm (-4SD), physical examination did not reveal any dysmorphic features. By 9 months of age, she was rolling and almost crawling at 13 months. There were concerns about choreoathetoid movement of her hands around the age of 7 months, but these subsequently settled: she was reviewed subsequently in a movement disorder clinic and felt not to have a definite movement disorder.

Ophthalmology assessment revealed delayed visual maturation. There were frequent reported staring episodes, but no concurrent epileptiform discharges noted on EEG. At 12 months of age MRI scan showed the ventricles are prominent for age with loss of white matter but preserved myelination. The pons was slightly small relative to the midbrain.

At 18 months she presents as a globally delayed child making forward progress. She sleeps well. She enjoys playing with books and listening to music. She is crawling and can sit with a straight back and pull to stand. Speech is limited to throaty sounds. Hearing is reportedly normal. She received movement therapy, speech and language therapy, occupational therapy, and physiotherapy.

The most recent growth at 4 years 5 months showed weight: 10.5kg (<<2^nd^ centile; Z-4.4), height: 86.5 cm (<<2^nd^ centile; Z-4.36). Her head circumference at 3years 4 months was 42cm (<<2^nd^ centile; Z= -4.83). Respiratory, cardiac, and abdominal examinations are normal. She has brisk reflexes.

Chromosomal microarray and karyotype were not diagnostic. Massively parallel sequencing panel (Fulgent) identified a *de novo* variant NM_001830.4(*CLCN4*):c.1664C>T; p.(Ala555Val).

**Family B15: NM_001830.4(*CLCN4*):c.1664C>T; p.(Ala555Val) *de novo***

This is a 10-year-old female referred to Genetics Clinic at age five for an evaluation of short stature, developmental delay, failure to thrive, and microcephaly. Family history was overall noncontributory with English, Mexican, and French ancestry.

She was born full term by spontaneous vaginal delivery. Birth weight was 2.89 kg (15^th^ centile) and length measured at 48.26 cm (50^th^ centile).

She was noted to have mild global developmental delay. Her first independent steps occurred at 18 months of age, and she spoke her first words at around 12 months. At age five she was speaking in short two-to-three-word phrases. As she aged, speech was about 40% understandable by strangers. Toilet training was achieved at age nine. Cognitive delays have also been noted requiring special education services in school.

Her growth has followed the third percentile for height and second for weight. Head circumference has remained below the third percentile.

A brain MRI and EEG were completed due to concern for possible seizure activity. Brain MRI was normal with no seizure focus identified. The EEG was mildly abnormal secondary to photo-paroxysmal response at 26 and 28 Hz.

At her most recent physical evaluation she was noted to have posteriorly rotated ears, slightly arched eyebrows, slightly depressed nasal bridge, decreased muscle bulk, prominent columella, fifth finger clinodactyly, mild to moderate microcephaly, proportionate small stature, and mild hypotonia were noted.

Exome sequencing identified a *de novo* variant NM_001830.4(*CLCN4*):c.1664C>T; p.(Ala555Val).

## Group C: Rare missense variant resulting in a loss-of-function on functional studies, with an additional confirmed or likely genetic diagnosis (blended phenotype)

**Family C1: NM_001830.4(*CLCN4*):c.100G>A; p.(Asp34Asn) maternally inherited**

The proband is a 9-year-old male with a confirmed diagnosis of the autosomal recessive skeletal dysplasia syndrome Desbuquois Dysplasia (*XYLT1*-related) characterized by short stature, joint laxity, and advanced carpal ossification. He was further investigated for an additional genetic condition due to a formal diagnosis of intellectual disability, epilepsy, and features of autism spectrum disorder, considered more severe than typical than what is known for *XYLT1*-related Desbuquois Dysplasia.

He was delivered at 40+6 weeks gestation. Prenatally he had a raised nuchal translucency detected in the first trimester and short long bones at 21 weeks gestation. Birth weight was 2.9kg (2^nd^-9^th^ centile); head circumference 35 cm (50^th^ centile) and height (<<0/4^th^ centile). There were no immediate post-natal complications. In infancy he was noted to have mild gross motor delay, walking independently at 18 months. His first words were at 18 months, and he received speech and language therapy.

Currently, aged 12 he is independently mobile and can talk in sentences.

He has had no regression of skills. He receives speech, physio, and occupational therapies.

He has a formal diagnosis of intellectual disability and although currently attends mainstream schooling, is likely to require specialist secondary education. He has features on the autism spectrum. He tends to have obsessive behaviors.

He has epilepsy with seizures first diagnosed at 3 months of age. Seizure types include focal, atonic, and febrile seizures. Seizures are not yet controlled on therapy with two antiepileptics, including sodium valproate. His brain MRI was normal.

He has had recurrent otitis media and requires hearing aids. He is overweight. He feeds orally. He has constipation.

On examination he has distinctive features including almond shaped eyes, pointed teeth and a short, upturned nose. He has rhizomelic limb shortening consistent with Desbuquois Dysplasia. At the age of 9 how height is <<0/4^th^ centile and head circumference between the 0.4th and 2nd centile.

He was identified as having a maternally inherited variant in *CLCN4* on trio exome sequencing (later confirmed by sanger sequencing) NM_001830.4(*CLCN4*):c.100G>A; p.(Asp34Asn), which was classified by the laboratory as a variant of uncertain clinical significance.

The variant was segregated further in the family and his sister, who also has a diagnosis of Desbuquois dysplasia, also has the *CLCN4* variant. She is currently 6 years old. She has no history of developmental delay and attends a mainstream school with additional support due to her short stature. She has no features of autism spectrum disorder. She has gastrointestinal reflux, constipation, and diarrhea.

Their mother has normal learning and no history of mental health diagnoses.

**Family C2: NM_001830.4(*CLCN4*):c.206C>T; p.(Ser69Leu) maternally inherited and a *de novo* novel missense variant in *SOX11.***

This is a 5-year-old child born to non-consanguineous Lebanese parents. He was the first-born child to the couple after two miscarriages. There was no family history of intellectual disability.

There were no prenatal complications, and he was delivered at term with a birth weight of 3.8 kg (50^th^ - 90^th^ centile), length 50 cm (50^th^ centile). There were no immediate postnatal complications.

He had delayed fine motor and speech development. He only has rudimentary speech at the age of 5 years. Sitting was achieved at seven months with walking at 3 years. He can eat independently. He does not receive any allied health support and has not had a full-scale psychometric (IQ) assessment but has an informal diagnosis of severe intellectual disability. He attends a regular school with support. He has impulsiveness and anger outbursts.

Other medical issues include a seventh cranial nerve palsy, severe myopia, mild ataxia, hypospadias, and recurrent buccal mycoses.

Neuroimaging (brain MRI) has been normal. He has no history of seizures and has had a normal EEG.

The last examination was at 5 years was consistent with a height of 104cm (10^th^ percentile) and a head circumference of 49 cm (12^th^ percentile). He has distinctive features including long eyelashes, a synophrys, thick eyebrows, low set ears, short but normal fifth digit phalanges in hands and feet, a second toe longer than the hallux on the left side. Clinical photographs were previously published in a short report (2).

Exome sequencing identified a maternally inherited variant in *CLCN4*: NM_001830.4(*CLCN4*):c.206C>T; p.(Ser69Leu). His mother was unaffected. He also was detected to have a *de novo* novel missense variant in *SOX11*; c.146T>A; p.(Ile49Asn). This variant was absent from gnomAD and predicted to be deleterious by SIFT (score = 0), probably damaging by PolyPhen2 (score = 0.998), disease-causing by Mutation Taster (score = 194), deleterious by PROVEAN (score = −6.469) and deleterious by CADD (score = 34). The affected residue was shown to be conserved across several species (Wakim et al., 2021). No functional studies have been performed to date to further assess the pathogenicity of this variant. However, the residue affected lay within the HMG domain, which is a DNA-binding domain essential for SOX11’s role as a transcriptional regulator (3) and four other *de novo* variants lying in this domain have been demonstrated to affect downstream transcriptional activity in in vitro studies (4, 5). Compared to the other 7 individuals reported with *de novo* variants in *SOX11,* this boy lacks the distinctive hypoplasia of the 5^th^ fingernail and toenail or clinodactyly of the 5^th^ finger reported

in all other individuals. Moreover, this boy lacks the microcephaly under the 3^rd^ centile reported in other individuals. The degree of intellectual disability for others with *SOX11* variants was mild for 5 out of the 7, but certainly severe intellectual disability and challenging behaviors/ autistic features were reported for some of the SOX11 cohort, despite mild intellectual disability is more typical for the group of SWI/SNF-related intellectual disability disorders as a group, into which *SOX11*-related neurodevelopmental disorders is suggested to fall (6).

It is thus difficult to disentangle the relevant importance of the *CLCN4* and *SOX11* variants in this individual, particularly as both are non-recurrent, and functional studies have yet to be completed on the *SOX11* variant. However, it is plausible that this individual has a blended phenotype with the *CLCN4* variant influencing the degree of intellectual disability in this individual.

**Family C3: NM_001830.4(*CLCN4*):c.1106C>T; (p.Pro369Leu) *de novo***

The proband is a 13-year-old male of mixed European/Caucasian heritage. There was no parental consanguinity, and the family history was otherwise non-contributory.

He was delivered via Caesarean section at 38 weeks gestation due to failure to progress. His birth weight was 3095g. His APGAR scores were 3 and 7 at 1 and 5 minutes, respectively. He was intubated immediately after birth because of a congenital diaphragmatic hernia (CDH) associated with left lung hypoplasia. He required extracorporeal membrane oxygenation (ECMO) for more than 30 days. Following surgical correction of the CDH, he had a cerebral venous thrombus and subsequent hydrocephalus requiring a ventriculoperitoneal shunt.

There was global developmental delay that was initially attributed to the post-operative complications and prolonged ECMO. He was later diagnosed with moderate ID at age 7 years. His current highest level of academic achievement is at elementary school level, and he has an individualized education plan. He walked independently for 4 years. He is currently, aged 13, able to speak in simple sentences, although is not always comprehensible to a stranger, and is able to follow one-step commands. He requires assistance with most activities of daily living: he can feed himself with a utensil and has a pincer grasp. He is seen by numerous allied health professionals including speech pathology, occupational therapy, and physiotherapy. There has not been any regression of skills.

He was diagnosed with autism spectrum disorder (ASD) at 8 years and has occasional anger outbursts and rigidity, for which there is behavioral support provided at school. He has been seen by a mental health professional and has not been diagnosed with other mental health disorders.

Seizures were diagnosed prior to the age of 1 years based on abnormal posturing. However, since infancy there have been no proven clinical seizures. The most recent EEG at 7 years of age years showed abundant epileptiform discharges over the left posterior quadrant with accompanied slowing and significant sleep activation. He was initially on levetiracetam, but this was changed to clobazam as there were concerns it may have contributed to his aggressive behavior. He remains stable on clobazam.

He currently has a wide-based but not frankly ataxic gait. He has tight calves bilaterally and wears ankle foot orthoses. He has bilateral sensorineural hearing loss for which hearing aids are needed. He has optic nerve hypoplasia and bilateral cryptorchidism. He requires feeding via gastrostomy tube and has severe gastroesophageal reflux disease.

Dysmorphic features include full cheeks, a long philtrum, micrognathia, wide nasal bridge, anteverted nares, frontal bossing, exaggerated cupid’s bow, downturned corners of mouth, high anterior hairline, a prominent forehead, lagophthalmos, hypertelorism, a preauricular pit, and posteriorly rotated ears.

In infancy, his karyotype was normal and chromosomal microarray analysis identified a ~172 kb 6q24 deletion of uncertain significance, which was subsequently found to be paternally inherited and considered non-diagnostic. Singleton exome sequencing at age 8 years identified a heterozygous paternally inherited VOUS in *LRP2* [NM_004525:c.11288A>T; p.(Glu3763Val)], the gene associated with Donnai-Barrow syndrome, for which he shares many of the characteristic phenotypic features. Trio genome sequencing performed on a research basis (study ID CMC_24 reported by Costain et al., 2020 (7)) identified the *de novo* variant in *CLCN4*: NM_001830.4*(CLCN4*):c.1106C>T; p.(Pro369Leu), and no second variant in *LRP2*. The *CLCN4* variant was subsequently found to have been detected by the prior clinical exome sequencing test but not reported out.

## Group D Phenotype and *in silico* features suggestive of *CLCN4* encephalopathy but unable to confirm a functional impact.

**Family D1:** **NM_001830.4(CLCN4):c.87C>G; p.(Asp29Glu) maternally inherited**

The proband is a 10-year-old male with moderate to severe intellectual disability. He has a 2 year 3-month-old brother with infantile spasms and global developmental delay. The brothers have an older maternal half-brother who is 19 with profound learning difficulties and challenging behavior, in whom segregation for the *CLCN4* variant has not yet been possible. Their mother has a mild intellectual disability. She was able to finish college and was a support worker for adults with mental health disorders. She cannot write and has difficulties with reading. There are two other maternal siblings with normal learning. Both affected males were born at term following quick deliveries. They did not require admission to the Special Care Baby unit.

The proband started sitting independently at 3 years. There are no concerns regarding his hearing. He wears glasses for a refractive error. At the age of 10 years, he presents with severe learning difficulties, hypotonia, slow weight gain and constipation. He has difficulty gaining weight, but his weight centiles have been slightly improving. He is on high calorie supplements. He is on medication for constipation. He attends a special school. He can stand supported in a frame and is able to take a few steps in the walking frame which is used in school. They have reported that he can manage up to 50 meters. He tends to finger feed and finds it difficult coordinating a fork and spoon. He can hold his cup. He has four meaningful words. He can shake his head for “no” but does not follow instructions though he shows some situational understanding. Most of his communication is through picture exchange system (PECS) and he occasionally points. He has anxiety and occasional anger outbursts. He has hyperkinesis and short attention span. He has support from speech, occupational and physiotherapy.

He has had no seizures. MRI brain showed multiple dilated perivascular spaces, delayed myelination, and a suggestion of grey matter heterotopia.

On examination, he sits with a curved back and has truncal and peripheral hypotonia with evidence of hyper-extensible joints. There is no excessive skin elasticity. Cranial nerve examination was normal. Reflexes are difficult to elicit. He has reasonable power for weight and hypotonia.

His younger brother manifested infantile spasms at the age of 6 months which had an effect on his developmental progress. These progressed to myoclonic/ tonic seizures. EEG demonstrated polyspike/spike and slow wave bursts some followed by decrement, correlating with clinical spasms. Seizures were initially 2-3 times per day in 15-minute clusters.

He was initially treated with prednisolone (UKISS protocol) and vigabatrin and then weaned down from the prednisolone and was put on to levetiracetam. He is still treated with levetiracetam (15 mls twice per day) and has been seizure free for at least the last 6 months. He has had an MRI scan that was reported as normal. He passed his hearing test and in under review for convergent strabismus and the possibility of nystagmus. At the age of 24 months, he is not sitting up unaided. He rolled at 10 months, he sometimes reaches for things, but not always. He babbles. He frequently claps and covers his ears. He has good eye contact. He is generally hypotonic and sits with a rounded back. I could not elicit lower limb reflexes. He has a moderate level of global developmental delay. He receives speech, occupational and physiotherapy. On examination both probands have overlapping dysmorphic facial features: a triangular/trapezoidal face shape, high anterior hairline and prominent forehead, deep-set eyes with straight eyebrows, long/large mouth with thin lips, square, widely spaced teeth. They also have a few mannerisms/involuntary movements: hand regard, hand, and arm flapping, clapping, smiling, and laughing most of the time. The 10-year-old proband is more obviously hyperkinetic whilst his younger brother has much better eye contact and interaction. Aged 10 the older brother had a head circumference of 53.5cm (50-98^th^ centile), weighed 19.55kg (<3^rd^ centile) and was 123 cm tall (just under 1^st^ centile). The younger brother at 2 years 3 months had a head circumference of 49cm (50^th^ centile), weighed 10kg (just under 3^rd^ centile) and was 85.2cm tall (50^th^ centile).

Both males have a maternally inherited VUS in NM_001830.4(*CLCN4*):c.87C>G; p.(Asp29Glu). No alternative possible genetic explanations have been identified to date. Normal investigations include creatinine kinase, thyroid function test, chromosomal microarray, urine for amino acids, organic acids, mucopolysaccharides and oligosaccharides. Plasma lactate, urate, white cell enzymes, very long chain fatty acids, common mitochondrial DNA mutations, phytanic acid, transferrin isoelectric focusing, common deletions of *SMN* gene (for spinal muscular atrophy), and testing of expansion of the genetic region for myotonic dystrophy type I.

**Family D5: NM_001830.4(*CLCN4*):c.944G>A; p.(Arg315His) *de novo***

This is a 3-year-old daughter to non-consanguineous parents of Uzbekistan heritage. Maternal age at conception was 23 and father 30 years old. There were prenatal concerns with intrauterine growth restriction and the delivery was induced at 36 weeks due to concerns regarding placental insufficiency and reduced fetal movements. Birth weight was 2.6kg (3^rd^ centile) and length 47 cm (15^th^ centile). She was discharged home after one week.

There were early concerns with developmental delay, with delayed motor (gross and fine) and speech delay. Aged 3 years she is not yet talking. She sat independently at >11 months. She can walk independently from 21 months. She can feed herself with her fingers. She receives early intervention including physiotherapy (from 10 months) and occupational and speech therapy. She has not yet had formal psychometric assessment but developmental assessment at 20 months is consistent with a moderate global developmental delay; Vinelands adaptive behavior scale - extremely functionally compromised. She also has an autism (Level 2) diagnosis.

She has only had one brief febrile seizure at 34 months. A postictal EEG (awake) was normal. She has never had neuroimaging. Her parents are trailing cannabinoid oil.

She feeds orally and has constipation. Current growth parameters: height 87cm (40^th^ centile); at 27 months; weight 13.4kg (70^th^ centile), head circumference 47.5 cm (50^th^ centile).

She has had no significant copy number abnormality on chromosomal microarray, no abnormality on urine metabolic screen, a normal *FMR1* gene test. Trio exome sequencing identified a *de novo* *CLCN4* variant NM_001830.4(*CLCN4*):c.944G>A; p.(Arg315His).

**Family D8: NM_001830.4(*CLCN4*):c.1090A>G; p.(Arg364Gly)**

This is a 27-year-old male of mixed white/ French Canadian heritage. There is a family history of his mother having intellectual disability and epilepsy. His mother had three maternal uncles and one maternal cousin with epilepsy. His mother had uncontrolled epilepsy during the pregnancy (6 seizures) despite therapy with ethosuximide, Phenytoin and Clonazepam. He was delivered at 39 weeks by caesarian section for fetal heart decelerations. Birth Apgar scores were 9 at 1,9 at 5 and 10 and 10 minutes. His birth weight was 3930g. He had immediate postnatal complication which were thought to be secondary to withdrawal from maternal antiepileptics.

He had normal gross motor milestones, walking at 16 months. He could tie his shoelaces at a normal age and was toilet trained by 3 ½ years. He had a formal expressive language delay. And a diagnosis of opposition defiant disorder as a child (resolved). His level of intellectual disability is borderline to mild. He is fully independent.

He has had epilepsy since the age of 6 year – absence seizures, focal seizures, and occasionally secondary generalized seizures 1-2 times a month, despite therapy with valproate and levetiracetam. EEG shows 2 episodes of spike-wave discharges associated with blinking and generalized cortical irritability with dysrhythmia.

He is otherwise generally well. He has mild asthma. Height is 179cm and weight 71.6 kg.

Chromosomal microarray is normal. He has a VUS identified on epilepsy panel NM_000626.2 (NAGA) c.973G>A, p.(Glu325Lys) in addition to the *CLCN4* variant.

**Family D11: NM_001830.4(*CLCN4*):c.1886C>T; p.(Thr629Ile) maternally inherited**

This is 14-year-old Caucasian female with developmental delay, intellectual disability, hypotonia and cyclic vomiting. Patient has some minor dysmorphic features including a long face and arched eyebrows. There is a strong family history of migraines.

She was born as the 3^rd^ daughter to a 34-year-old father and 35-year-old mother. Her mother had one prior miscarriage in the first trimester. The pregnancy with this child was uncomplicated other than the detection of a 2-vessel umbilical cord on antenatal ultrasound. The mother reported less fetal movements compared to her other pregnancies. The child was born via uncomplicated vaginal delivery after 38 weeks of pregnancy.

The birth weight was 2.8kg. There were no postnatal complications and she breast-fed. Her parents noticed developmental delay at the age of 1 year. She sat at 18 months of age, walked at the age of 3 years, and started to speak at the age of 5 years. At age of 7 years, she can walk and run, climb stairs but she is clumsy and falls a lot. She can now speak 200 words and use short sentences. Her receptive communication is good. She can follow short commands. She can initiate eye contact, but her attention span is short. She loves to play with other children and there is no aggression or other behavioural issues. She "wants to please" according to her parents.

She underwent two brain MRI scans (aged 3 and 5 years) which showed a small remote lacunar infarct and dilated perivascular space, but no major abnormalities were found. She also had who head CT scans that demonstrated no abnormalities. Aged 6, she underwent extensive blood tests during a vomiting episode: serum amino acids profile was normal, acylcarnitine profile was normal, urine amino and organic acids were normal except for high Alanine (was 74, normal is below 25). She was also tested for CDG (congenital diseases of glycosylation), and this was non diagnostic. GAMT was normal. Her CK and thyroid function tests were normal. She has no history of high blood lactate. She has been under general anaesthesia 3 times in the past (ear tubes, endoscopy, adenoidectomy) with no special events during or after procedures. She had an ophthalmological exam which was normal except for pseudo-strabismus. She has mild conductive hearing loss that was attributed to recurrent ears infection, ear tubes and imperforated ear drums. Hearing screen at the age of 13 months was normal.

As a newborn and infant, she had gastro-oesophageal reflux but out-grew it. She has constipation and requires disimpactions intermittently. She underwent Upper GI test at the age of 7 months and endoscopy at 3.5 years of age. These tests were normal. Chromosomal microarray was reported as normal. *MECP2* sequencing and deletion/duplication analysis was reported as normal. Whole exome sequencing identified a maternally inherited NM_001830.3(*CLCN4*):c.1886C>T; (p.Thr629Ile) which was classified as a variant of uncertain significance. No other candidate variants were identified.

## Group E: Truncating variants

**Family E1: NM_001830.4(*CLCN4*): c.925_928del; p.(Asn309fs) maternally inherited**

The proband is a 52-year-old Caucasian male. In childhood there was speech delay and he received speech therapy. There is a formal diagnosis of mild-moderate intellectual disability (Wechsler Adult Intelligence Scale full scale IQ 52). He can write his own name, but otherwise cannot read or write, tell the time, or make financial transactions. He left school at age 20 after having completed special education. He now works in supported employment.

He suffered from a psychotic episode and was noted to have sexual disinhibition. The age of diagnosis is not clear but was established by his late 20s. He is treated with risperidone.

Other medical issues include multiple naevi and verrucae seborroica with a few angioma. He has pes planovalgus. There is mild left-sided hearing loss with deterioration over higher frequencies: this has been attributed to presbycusis.

On most recent examination, aged 49, his height was 195 cm (>97^th^ centile; +1,7 SD), weight 90 kg (90-97^th^ centile; +1,5 SD) and head circumference 59.5 cm (>97^th^ centile; +1 SD). He has subtle facial dysmorphic features including prominent ears with simple helices and a prominent nose.

A targeted gene panel of an exome sequencing backbone for intellectual disability was performed. The following maternally inherited variant was identified: NM_001830.4(*CLCN4*):c.925_928del; p.(Asn309fs). His mother does not have symptoms. Mother’s father had Menière’s disease and had hemiplegia. Mother’s paternal grandmother also had Menière’s disease and was hospitalized in a mental institution.

**Family E2: NM_001830.4(*CLCN4*):c.1987_1990del; p.(Gln663Glyfs) maternally inherited**

The proband is an 18-year-old Caucasian male with mild to moderate intellectual disability, ADHD, and epilepsy.

He was delivered at term with no prenatal or perinatal complications. Birth measurements are unavailable. He presented with developmental delay in infancy with no history of hypotonia. He walked around 12 months and spoke his first words at 24 months. At age 18, he speaks in sentences, counts from 1-20 and follows simple one-step commands, but doesn’t understand time or money. He requires supervision and assistance with all activities of daily living, including toileting. He does not meet the criteria for ASD and has no mental health diagnoses. He attends a special education class (‘IM’) in a mainstream school.

He developed generalized seizures at 10.5 months. These have been well controlled for the past four years on a combination of lamotrigine 250mg mane and 150mg nocte; topiramate 25mgs BD and sodium valproate 200mg BD.

He is otherwise healthy with no gastrointestinal symptoms. He continues to have disturbed sleep, waking several times each night.

At age 13, growth measurements were: height 150cm (25^th^ centile) and head circumference 51.3cm (<2^nd^ centile). He has a long face with a prominent chin, straight eyebrows, and thin lips.

He was found to have a maternally inherited variant NM_001830.4(*CLCN4*):c.1987_1990del; p.(Gln663Glyfs) on whole genome sequencing. His mother has normal (75%) X inactivation and is clinically unaffected.

**Family E3: NM_001830.4(*CLCN4*):c.2025C>A: p.(Tyr675*) *de novo***

This is a 13-year-old male child born to healthy, non-consanguineous Indian-Surinamese parents. He has an older sister, who had febrile seizures. Otherwise, family history is unremarkable.

He was born at term after an uncomplicated pregnancy with normal birth parameters.

Before the age of 1-year febrile seizures started, shortly thereafter followed by tonic-clonic seizures. Under medication seizures continued till the age of 7 years, currently he is still seizure-free with low dose anti-epileptic drug.

His motor milestones were reached late, he walked independently at 18 months of age. His speech development was severely delayed. At the age of 13 years, he speaks a few words, and uses the same words for different things. Only people that know him well can understand him.

At the age of 9 years his cognitive developmental level was at 2 years. He has been diagnosed with a severe intellectual disability. He is a very lively, playful, enthusiastic, and friendly boy, however when frustrated his mood changes within seconds and he may get very aggressive. He is restless and hyperactive. He cannot stay indoors on his own, he is very impulsive and does not see danger. He can only go outside under strict supervision with people familiar with his habits and behavior.

He has sleeping problems, he needs someone nearby when he falls asleep and during the night, to avoid anxiety.

On clinical examination at the age of 9 years and 6 months he had a generalized hypotonia. Growth parameters were within normal range. He had no dysmorphisms.

Brain MRI showed bilateral mesial temporal sclerosis, asymmetric, with the right side more affected.

Exome sequencing (trio analysis) detected a *de novo* nonsense variant in *CLCN4*: NM_001830.4:c.2025C>A; p.(Tyr675*).

# Supplementary Figure 1: Summary of electrophysiological results for all tested variants.

D29E

D34N

K62R

S69L

G78S

D89N

V92M

S105C

T203I

V212G

P226L

F238L

F268L

G269D

I272V

V275M

V275L

L276F

S278R

L279V

E280D

S283N

N309S

P310S

R315H

V317F

V317I

F319S

R360S

R364G

G342E

P369L

I374T

S395R

R432Q

G484R

Q489K

G526S

V533M

V536M

G544R

G545D

G545S

I549N

I549L

A555V

K560E

V550L

D621G

T629I

P635R

V636M

I646T

R652T

I655V

R718W

For each variant, average IVs as in Figure 4B, and the ratio of mutant and WT currents as in Figure 4C are shown. For several variants that showed significant current levels, in addition the relative size of inward currents at pH 5.3 was determined as in Figure 4G. For each variant at least three batches of oocytes were injected and for each batch at least six oocytes were measured as described in Methods.

R718Q

G731V

G731R

# Supplementary Figure 2: The novel single nucleotide variant present in proband and his mother from family A52 very likely does not affect splicing of *CLCN4* transcripts.


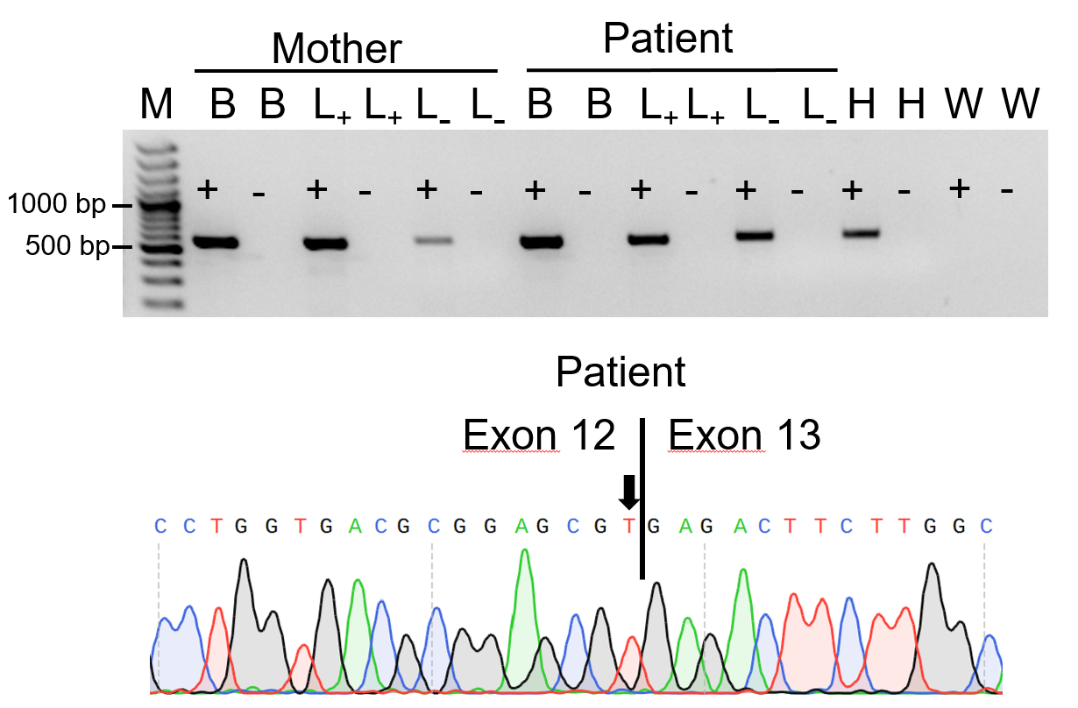


The single nucleotide variant *CLCN4* (NM_001830.4):c.2192G>T from family A42 altered the last base of exon 12 (indicated by the arrow in Sanger sequencing chromatogram) with controversial *in silico* predictions on potential splicing defect (e.g., from Varsome: ADA Boost score = 1 is greater than 0.708, SpliceAI ≤ 0.2).

We have investigated if this variant could have an effect on splicing in blood (B) and lymphoblastoid cell lines (L) from the affected male and his mother. Cell lines were established using a standard protocol and treated with puromycin for six hours (L_+_) for blocking nonsense mediated mRNA decay and untreated (L_-_). Automated total RNA extraction was performed using Total RNA Kit Maxwell® 16 Cell LEV Total RNA Purification kit (Promega) as recommended by the supplier. The cDNA synthesis was performed using SuperScript III reverse transcriptase (Invitrogen). In addition, human wildtype RNA extracted from HEKT cells (H) was used as positive control. To amplify the *CLCN4* mRNA between exon 11 and exon 13, the following primer pair was used: CLCN4_ex11_RT forward 5`-GAGGCCCACATCCACTTAAA-3` and CLCN4_ex13_RT reverse 5`-GATTCGGGGTCCTGGTTT-3`. RT-PCR products were separated in 2% agarose gels in TAE buffer extracted from the gel slices and Sanger sequenced using the same primers. Both proband and his mother had only one specific *CLCN4* RT-PCR product of 561 bp corresponding to wild-type *CLCN4*, suggesting that the single nucleotide variant likely has no effect on splicing.

+ = reverse transcriptase added, - = mock reaction without reverse transcriptase, M = size marker, W = negative control.

# Supplementary Figure 3: Clinical photographs of one affected male and one brother and sister from Group C with blended phenotype likely caused by another monogenic condition in addition to *CLCN4* missense change.


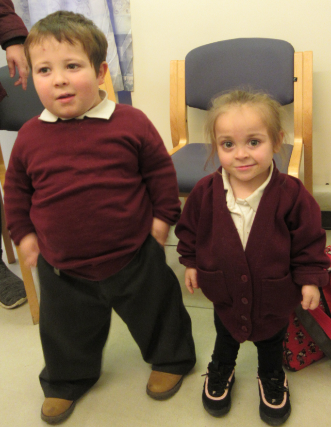
**
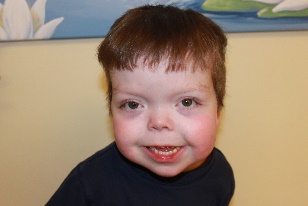

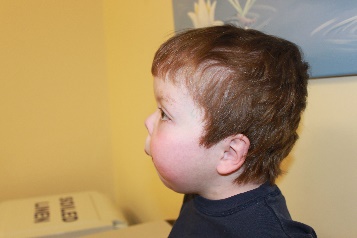
**
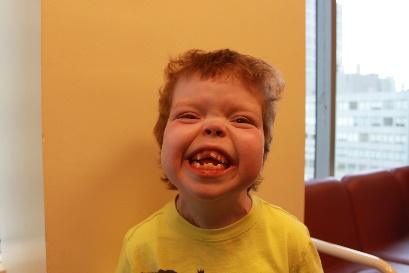

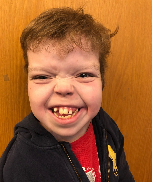


Family C1: p.(Asp34Asn); LOF, brother aged 9 years, sister aged 6 years. Confirmed additional diagnosis Desbuquois dysplasia (*XYLT1*-related).

Family C3: p.(Pro369Leu); LOF, aged 4 years, 7 years and 11 years. Possible additional diagnosis of Donnai-Barrow syndrome.

# REFERENCES

1. Palmer EE, Stuhlmann T, Weinert S, Haan E, Van Esch H, Holvoet M, et al. De novo and inherited mutations in the X-linked gene CLCN4 are associated with syndromic intellectual disability and behavior and seizure disorders in males and females. Molecular Psychiatry. 2018;23(2):222-30.

2. Wakim V, Nair P, Delague V, Bizzari S, Al-Ali MT, Castro C, et al. SOX11-related syndrome: report on a new case and review. Clin Dysmorphol. 2021;30(1):44-9.

3. Dodonova SO, Zhu F, Dienemann C, Taipale J, Cramer P. Nucleosome-bound SOX2 and SOX11 structures elucidate pioneer factor function. Nature. 2020;580(7805):669-72.

4. Hempel A, Pagnamenta AT, Blyth M, Mansour S, McConnell V, Kou I, et al. Deletions and de novo mutations of SOX11 are associated with a neurodevelopmental disorder with features of Coffin-Siris syndrome. J Med Genet. 2016;53(3):152-62.

5. Tsurusaki Y, Koshimizu E, Ohashi H, Phadke S, Kou I, Shiina M, et al. De novo SOX11 mutations cause Coffin-Siris syndrome. Nat Commun. 2014;5:4011.

6. Bogershausen N, Wollnik B. Mutational Landscapes and Phenotypic Spectrum of SWI/SNF-Related Intellectual Disability Disorders. Front Mol Neurosci. 2018;11:252.

7. Costain G, Walker S, Marano M, Veenma D, Snell M, Curtis M, et al. Genome Sequencing as a Diagnostic Test in Children With Unexplained Medical Complexity. JAMA Netw Open. 2020;3(9):e2018109.
